# LPGAT1/LPLAT7 regulates acyl chain profiles at the *sn*-1 position of phospholipids in murine skeletal muscles

**DOI:** 10.1016/j.jbc.2023.104848

**Published:** 2023-05-20

**Authors:** Tomoki Sato, Shuhei Umebayashi, Nanami Senoo, Takumi Akahori, Hiyori Ichida, Noriyuki Miyoshi, Takuya Yoshida, Yuki Sugiura, Naoko Goto-Inoue, Hiroki Kawana, Hideo Shindou, Takashi Baba, Yuki Maemoto, Yasutomi Kamei, Takao Shimizu, Junken Aoki, Shinji Miura

**Affiliations:** 1 Laboratory of Nutritional Biochemistry, Graduate School of Nutritional and Environmental Sciences, University of Shizuoka, Shizuoka, Japan; 2 Laboratory of Biochemistry, Graduate School of Nutritional and Environmental Sciences, University of Shizuoka, Shizuoka, Japan; 3 Laboratory of Clinical Nutrition, Graduate School of Environmental and Symbiotic Sciences, Prefectural University of Kumamoto, Kumamoto, Japan; 4 Department of Biochemistry, Keio University School of Medicine, Tokyo, Japan; 5 Department of Marine Science and Resources, College of Bioresource Sciences, Nihon University, Fujisawa, Japan; 6 Department of Health Chemistry, Graduate School of Pharmaceutical Sciences, The University of Tokyo, Tokyo, Japan; 7 Advanced Research & Development Programs for Medical Innovation (AMED-LEAP), Tokyo, Japan; 8 Department of Lipid Life Science, National Center for Global Health and Medicine, Tokyo, Japan; 9 Department of Lipid Medical Science, Graduate School of Medicine, The University of Tokyo, Tokyo, Japan; 10 Laboratory of Molecular Cell Biology, School of Life Sciences, Tokyo University of Pharmacy and Life Sciences, Hachioji, Japan; 11 Laboratory of Molecular Nutrition, Graduate School of Environmental and Life Science, Kyoto Prefectural University, Kyoto, Japan; 12 Department of Lipid Signaling, National Center for Global Health and Medicine, Tokyo, Japan; 13 Institute of Microbial Chemistry, Tokyo, Japan

**Keywords:** glycerophospholipid, phospholipid, phospholipid metabolism, phospholipid turnover, skeletal muscle, slow-twitch, fast-twitch, acyltransferase, transcription factor, peroxisome proliferator-activated receptor γ coactivator-1α

## Abstract

Skeletal muscle consists of both fast- and slow-twitch fibers. Phospholipids are important structural components of cellular membranes, and the diversity of their fatty acid composition affects membrane characteristics. Although some studies have shown that acyl chain species in phospholipids differ among various muscle fiber types, the mechanisms underlying these differences are unclear. To investigate this, we analyzed phosphatidylcholine (PC) and phosphatidylethanolamine (PE) molecules in the murine extensor digitorum longus (EDL; fast-twitch) and soleus (slow-twitch) muscles. In the EDL muscle, the vast majority (93.6%) of PC molecules was palmitate-containing PC (16:0-PC), whereas in the soleus muscle, in addition to 16:0-PC, 27.9% of PC molecules was stearate-containing PC (18:0-PC). Most palmitate and stearate were bound at the *sn*-1 position of 16:0- and 18:0-PC, respectively, and 18:0-PC was found in type I and IIa fibers. The amount of 18:0-PE was higher in the soleus than in the EDL muscle. Peroxisome proliferator-activated receptor γ coactivator-1α (*PGC-1α*) increased the amount of 18:0-PC in the EDL. Lysophosphatidylglycerol acyltransferase 1 (*LPGAT1*) was highly expressed in the soleus compared with that in the EDL muscle and was upregulated by PGC-1α. *LPGAT1* knockout decreased the incorporation of stearate into PC and PE *in vitro* and *ex vivo* and the amount of 18:0-PC and 18:0-PE in murine skeletal muscle with an increase in the level of 16:0-PC and 16:0-PE. Moreover, knocking out *LPGAT1* decreased the amount of stearate-containing phosphatidylserine (18:0-PS), suggesting that LPGAT1 regulated the acyl chain profiles of phospholipids, namely, PC, PE, and PS, in the skeletal muscle.

Skeletal muscle consists of both fast-twitch fibers (type II fibers) and slow-twitch fibers (type I fibers), which differ in their contractile and metabolic characteristics and are composed of a mosaic of different fiber types. Type I fibers are red because they are rich in myoglobin and possess mitochondria, their metabolism is aerobic, and they maintain relatively sustained contractions. Type II fibers are classified into type IIa, IIx, and IIb fibers. Type IIx and IIb fibers, which contain few mitochondria, are white; they derive their energy mainly from anaerobic glycolysis and exhibit relatively short durations of contraction. Although type IIa fibers are fast-twitch, they have a high aerobic capacity and properties similar to those of type I fibers. Endurance exercise training promotes fiber-type switching from fast- to slow-twitch muscles to acquire endurance exercise capacity. Peroxisome proliferator-activated receptor γ coactivator-1α (*PGC-1α*) plays a key role in this adaptation. PGC-1α, identified as a nuclear receptor coactivator, is expressed in brown adipose tissue, skeletal muscle, heart, kidney, and brain and is markedly upregulated in brown adipose tissue and skeletal muscle after acute exposure to cold stress (1). *PGC-1α* expression is induced in skeletal muscle by exercise (2, 3, 4) and controls many physical activity adaptations, such as fiber-type switching, mitochondrial biogenesis, fatty acid oxidation, and angiogenesis (5, 6, 7). Additionally, skeletal muscle overexpressing *PGC-1α* exhibited improved Ca^2+^ regulation following contractions (8).

In skeletal muscles, fatty acid profiles of glycerophospholipids (phospholipid profiles) change with adaptational responses induced by exercise training (9, 10, 11, 12), diet composition (13, 14), or atrophic changes due to denervation or fasting (15, 16). Thus, changes in phospholipid profiles are involved in functional alterations in skeletal muscles. We had previously revealed that overexpression of *PGC-1α* increases the amount of stearoyl-docosahexaenoyl-phosphatidylcholine [PC (18:0–22:6)] and stearoyl-docosahexaenoyl-phosphatidylethanolamine [PE (18:0–22:6)] in skeletal muscle (12). Furthermore, we clarified that voluntary exercise training increases the amounts of PC (18:0–22:6) and PE (18:0–22:6) in skeletal muscle *via* the expression of *PGC-1α* (12). Recently, a study on the relationship between muscle fiber types and specific lipid molecules was performed using imaging mass spectrometry (Imaging-MS) and thin-layer chromatography (TLC)-blot-matrix-assisted laser desorption/ionization (MALDI)-Imaging-MS (17). The study showed that the amount of PC (16:0–22:6) was lower, and the amount of PC (18:0–22:6) was higher in the soleus than in the extensor digitorum longus (EDL) muscles. On using the skeletal muscles of rats, palmitic acid (16:0) in the phospholipid fraction was lower in the soleus than in the EDL muscles, and stearic acid (18:0) was higher in the soleus than in the EDL muscles (18). Furthermore, in human skeletal muscles, type I fiber distribution was negatively correlated with the amount of 16:0 and positively correlated with 18:0/16:0 in the phospholipids (10). As over 70% of the total phospholipids in mammalian skeletal muscle are PC or PE (19), the alterations in the fatty acid species in the phospholipid fraction may be attributed to the fatty acid species bound to PC and PE. Although some studies have shown that differences in the fatty acid species that bind to phospholipids are related to skeletal muscle fiber type, the composition of intact phospholipid molecules and the mechanisms behind these differences are still not fully understood.

Therefore, in this study, to reveal the mechanism for the production of stearic acid-containing PC and PE molecules (18:0-PC and 18:0-PE, respectively) in murine skeletal muscle, we compared the phospholipid profiles between EDL and soleus using liquid chromatography-MS (LC-MS). In addition, we used mice overexpressing *PGC-1α* specifically in the skeletal muscles (*PGC-1α* Tg mice) as a model of acquired slow-twitch muscle. Moreover, we investigated acyltransferase as a candidate for the production of 18:0-PC and 18:0-PE in the skeletal muscle by analyzing genetically modified mice.

## Results

### Muscle fiber type-specific differences and PGC-1α-mediated changes in acyl chain profiles in muscular PC and PE

**Figure 1:**
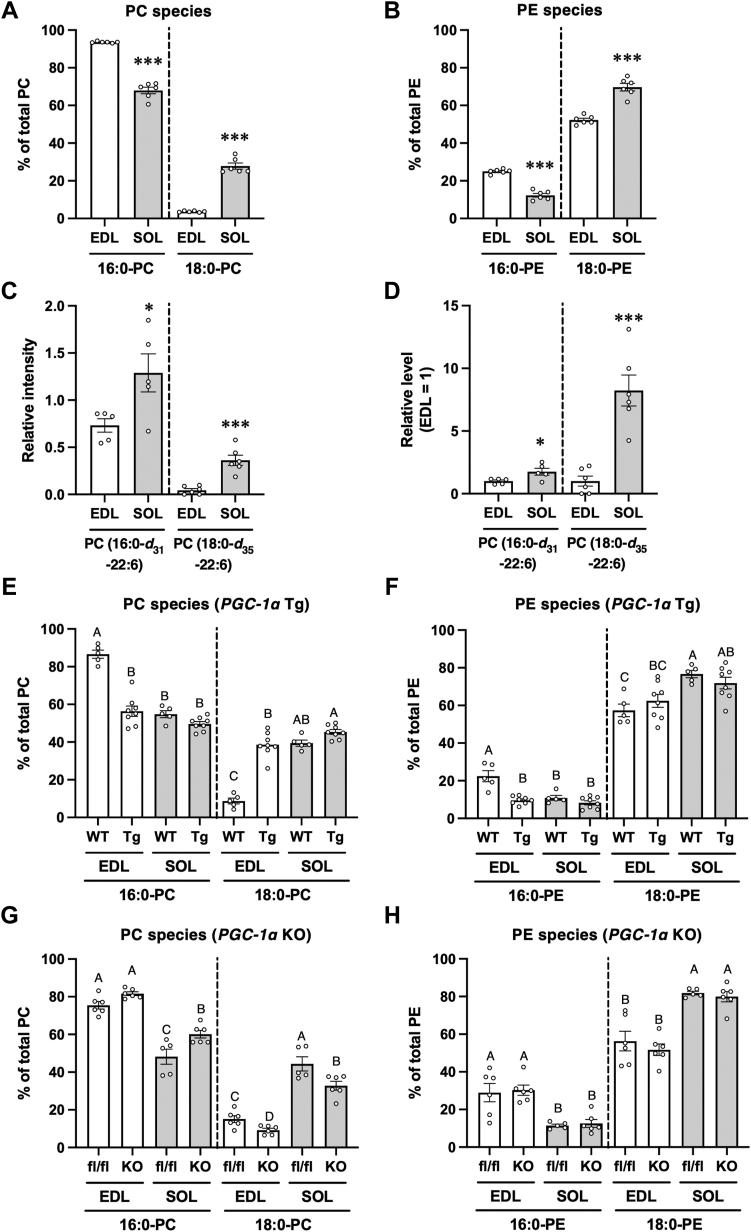
**Muscle fiber type-specific differences in the acyl chain profiles of phospholipids and involvement of PGC-1α.***A* and *B*, amounts of 16:0 and 18:0-PC (*A*) and PE (*B*) in the extensor digitorum longus (EDL) and soleus (SOL) muscles of C57BL/6J mice. Values are represented as the mean ± standard error of the mean (SEM) (n = 6). ∗∗∗*p* < 0.001 (*versus* EDL). *C* and *D*, EDL and SOL were treated with 0.625 mM of palmitic-*d*_31_ acid (16:0-*d*_31_) or stearic-*d*_35_ acid (18:0-*d*_35_) in the presence of 0.625 mM of non-labeled stearic acid or palmitic acid, respectively. The levels of PC (16:0-*d*_31_-22:6) and PC (18:0-*d*_35_-22:6) were measured using LC-MS/MS. The ion intensities of each PC species were normalized against the ion intensities of the internal standard and presented as “Relative intensity” (*C*). The relative PC levels of PC (16:0-*d*_31_-22:6) and PC (18:0-*d*_35_-22:6) when the value of EDL is “1” in the respective PC species are plotted (*D*). Values are represented as the mean ± SEM (n = 5–6). ∗*p* < 0.05; ∗∗∗*p* < 0.001 (*versus* EDL). *E* and *F*, amounts of 16:0 and 18:0-PC (*E*) and PE (*F*) in the EDL and SOL muscles of wild-type mice (WT) and *PGC-1α* transgenic (*PGC-1α* Tg) mice (Tg). Values are represented as the mean ± SEM (n = 5–8). Means without a common letter differ significantly (*p* < 0.05). *G* and *H*, amounts of 16:0 and 18:0-PC (*G*) and PE (*H*) in the EDL and SOL muscles of *PGC-1α*^flox/flox^ mice (fl/fl) and *PGC-1α* knockout (*PGC-1α* KO) mice (KO). Values are represented as the mean ± SEM (n = 6). Means without a common letter differ significantly (*p* < 0.05).

To examine the differences in the acyl chain profiles of PC and PE between fast- and slow-twitch muscles, namely, EDL and soleus, respectively, PC and PE molecular species were analyzed using LC-MS with precursor ion and neutral loss scanning mode, respectively. The amounts of PC molecules detected in the EDL and soleus are listed in Table S1. The amounts of 16:0-PC, such as PC (16:0–16:1), PC (16:0–18:2), PC (16:0–20:4), and PC (16:0–22:6), were higher in the EDL than in the soleus. The amounts of 18:0-PC, such as PC (18:0–18:2), PC (18:0–18:1), PC (18:0–20:4), and PC (18:0–22:6), were higher in the soleus than in the EDL. Figure 1*A* shows the differences in the fatty acid profiles of PC between the EDL and soleus. In the EDL, 93.58 ± 0.20% of PC was 16:0-PC; however, only 3.67 ± 0.15% of PC was 18:0-PC. In the soleus, 67.98 ± 1.91% of PC was 16:0-PC, and 27.85 ± 1.63% of PC was 18:0-PC. Thus, EDL contained mainly 16:0-PC species, whereas soleus contained not only 16:0-PC but also 18:0-PC species. Similar PC profiles were also obtained in rat skeletal muscle (Fig. S1). The amounts of PE molecules detected in the EDL and soleus are listed in Table S2. Figure 1*B* shows the differences in the fatty acid profiles of PE between the EDL and soleus. PE profiles of the EDL and soleus also differed similar to the differences between their PC profiles, while the amount of 16:0-PE, such as PE (16:0–22:6), and the amount of 18:0-PE, such as PE (18:0–22:6), were higher in the soleus than in the EDL. In contrast to PC molecules, the amount of 18:0-PE was much higher than that of 16:0-PE in both EDL and soleus. In contrast to the findings in the murine sample, no difference was observed in the amount of 18:0-PE between the EDL and soleus in rat skeletal muscle (Fig. S1).

To determine whether fast- and slow-twitch muscles incorporate different fatty acid species into PC, stable isotope tracer experiments were performed using palmitic-*d*_31_ acid (16:0-*d*_31_) and stearic-*d*_35_ acid (18:0-*d*_35_). After the EDL and soleus incubation with the deuterium fatty acid, the amounts of PC (16:0-*d*_31_-22:6) and PC (18:0-*d*_35_-22:6) were measured using LC-MS/MS because these species constituted the majority 16:0-PC and 18:0-PC species in the skeletal muscle. The amount of PC (16:0-*d*_31_-22:6) and PC (18:0-*d*_35_-22:6) were much higher in the soleus than in the EDL, suggesting that the rate of exogenous fatty acid incorporation into PC was higher in the soleus muscle (Fig. 1*C*). The greatest difference between EDL and soleus was found in the respective amounts of PC (18:0-*d*_35_-22:6), which was 8.2 times higher in the soleus than in the EDL muscle (Fig. 1*D*). By contrast, the amount of PC (16:0-*d*_31_-22:6) was only 1.8 times higher in the soleus than in the EDL.

As overexpression of *PGC-1α*-induced fiber-type switching from fast-to slow-twitch muscles (5, 6, 7), the same experiments were performed in the EDL and soleus muscles overexpressing *PGC-1α* to confirm whether acyl chain profiles in PC and PE are modified, concomitantly with fiber-type switching. The amounts of PC and PE molecules detected in skeletal muscles overexpressing *PGC-1α* are shown in Tables S3 and S4, respectively. The amounts of 18:0-PC, such as PC (18:0–18:2), PC (18:0–18:1), PC (18:0–20:4), and PC (18:0–22:6), were increased, and those of 16:0-PC, such as PC (16:0–16:1), PC (16:0–20:4), and PC (16:0–22:6), were decreased in the EDL muscle upon overexpression of *PGC-1α*. Figure 1*E* shows that overexpression of *PGC-1α* in the EDL caused an increase in the levels of 18:0-PC with a concomitant decrease in the amount of 16:0-PC. The PC profiles of *PGC-1α*-overexpressing EDL were similar to the corresponding profile of the soleus. Additionally, the amount of 16:0-PE, such as PE (16:0–22:6), was decreased in EDL *via PGC-1α* overexpression. However, no changes were observed in 18:0-PE upon *PGC-1α* overexpression (Fig. 1*F*). These results showed that the amount of 18:0-PC was significantly higher in the slow-twitch muscles, such as soleus, and in acquired slow-twitch muscles, such as *PGC-1α*-overexpressing EDL, than in fast-twitch muscles. Furthermore, we investigated whether the loss of *PGC-1α* could modulate the amounts of 18:0-PC and 18:0-PE (Fig. 1, *G* and *H*; Tables S5 and S6). The amount of 18:0-PC was significantly decreased in both *PGC-1α*-knockout EDL and soleus muscles compared with that in *PGC-1*α^flox/flox^ mice (Fig. 1*G*). However, no changes were observed in 18:0-PE by knocking out *PGC-1*α (Fig. 1*H*).

### Localization of phospholipid species in frozen sections of murine skeletal muscle and characterization of PC (16:0–22:6) and PC (18:0–22:6) in skeletal muscle

**Figure 2:**
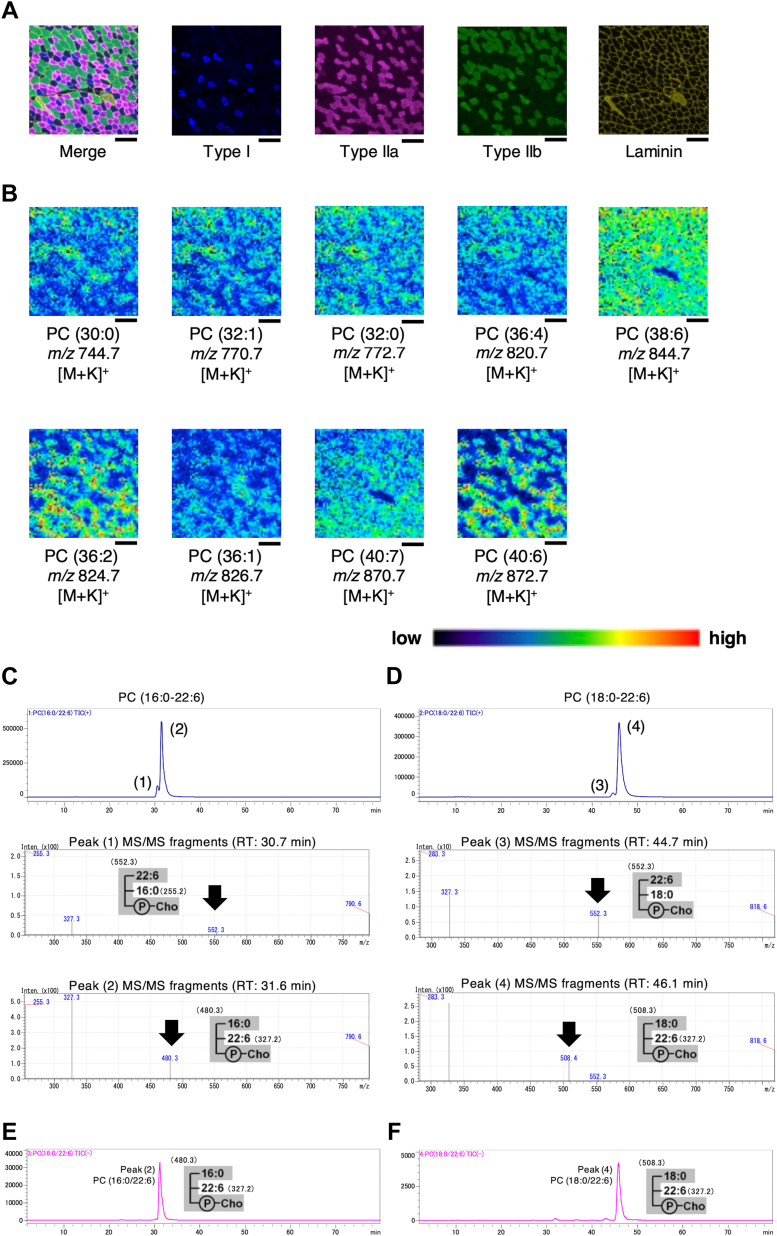
**Localization of PC species in the frozen sections of murine skeletal muscle and characterization of PC (16:0–22:6) and PC (18:0–22:6) in skeletal muscle.***A*, immunofluorescence images of myosin heavy chain (MHC) type I, IIa, and IIb, and laminin fibers using specific antibodies. Gastrocnemius section was analyzed. *B*, molecular images of PC species in a section of the gastrocnemius muscle. Scale bar: 200 μm. *C*, the mass chromatogram obtained using MRM (*m/z* 806.50 > 184.00) for synthetic PC (16:0/22:6) was separated into two peaks. Tandem mass spectrometry (MS/MS) fragments of each forward and backward peak are shown in (1, 2), respectively. *D*, the mass chromatogram obtained using MRM (*m/z* 834.50 > 184.00) for synthetic PC (18:0/22:6) was separated into two peaks. MS/MS fragments of each forward and backward peak are shown in (3, 4), respectively. *E* and *F*, analysis of the positional isomers of PC (16:0–22:6) (*E*) and PC (18:0–22:6) (*F*) in the lipid extracts from the gastrocnemius muscle.

To investigate whether the difference in acyl chain profiles of PC in the EDL and soleus muscles were dependent on the muscle fiber types, Imaging-MS and immunofluorescence imaging analyses were performed with serial sections of the gastrocnemius, which were composed of type I, IIa, IIx, and IIb fibers. First, we performed immunofluorescence imaging analysis using antibodies against types I, IIa, and IIb (Fig. 2*A*). Unstained fibers were classified as type IIx. Figure 2*B* shows the representative images from Imaging-MS analyses, showing the molecular image of the PC species. The molecular ions at *m/z* of 770.7 [PC (32:1)] and 820.7 [PC (36:4)] were mainly localized in fast-twitch fiber, such as type IIb (Fig. 2*B*). The molecular ions at *m/z* of 772.7 [PC (32:0)] and 844.7 [PC (38:6)] were found to be ubiquitous (Fig. 2*B*). The molecular ions at *m/z* 824.7 [PC (36:2)] and 872.7 [PC (40:6)] were mainly localized to slow-twitch fibers, such as type I and IIa fibers (Fig. 2*B*). Using the molecular weight data of PC species detected in the skeletal muscle using LC-MS/MS analysis as a reference (Table S1), PC (30:0), PC (32:1), PC (32:0), PC (36:4), PC (38:6), PC (36:2), PC (36:1), PC (40:7), and PC (40:6) were estimated to correspond to PC (14:0–16:0), PC (16:0–16:1), PC (16:0–16:0), PC (16:0–20:4), PC (16:0–22:6), PC (18:0–18:2), PC (18:0–18:1), PC (18:1–22:6), and PC (18:0–22:6), respectively.

Glycerophospholipids, such as PC and PE, vary according to the relative positions of the two acyl chains on the glycerol backbone (*sn*-positional isomers). To determine the position of the acyl chains, we characterized PC (16:0–22:6) and PC (18:0–22:6), which are the main molecules of 16:0-PC and 18:0-PC in murine skeletal muscle, using LC-MS. According to a previous report, 1-acyl-2-lyso-PC fragment ions produced by loss of the *sn*-2 moiety were detected as a peak higher than the one that 2-acyl-1-lyso-PC fragment ions produced by loss of the *sn*-1 moiety (20, 21). When the synthetic PC (16:0–22:6) was analyzed under multiple reaction monitoring (MRM) to capture PC-specific fragment ions at Q3, two peaks were detected at 30.7 [peak (1)] and 31.6 min [peak (2)] (Fig. 2*C*). Tandem mass spectrometry (MS/MS) analysis of acyl chain-derived fragments revealed that the fragments corresponding to 1-docosahexaenoyl-2-lysophospholipid or 1-palmitoyl-2-lysophospholipid were higher in peaks (1, 2) that corresponded to 1-docosahexaenoyl-2-palmitoyl PC and 1-palmitoyl-2-docosahexaenoyl PC, respectively. Similarly, Figure 2*D* shows that peak (3) (retention time: 44.7 min) and peak (4) (retention time: 46.1 min) corresponded to 1-docosahexaenoyl-2-stearoyl PC and 1-stearoyl-2-docosahexaenoyl PC, respectively. The PC (16:0–22:6) was analyzed in the gastrocnemius muscle, for which the peak was detected only at 31.6 min (Fig. 2*E*), showing that PC (16:0–22:6) in the gastrocnemius, the main molecule of 16:0-PC, was 1-palmitoyl-2-docosahexaenoyl PC. Using the same method, PC (18:0–22:6), the main molecule of 18:0-PC, was determined to be 1-stearoyl-2-docosahexaenoyl PC (Fig. 2*F*).

### Differences in gene expression involved in fatty acid profiles of PC and PE between fast- and slow-twitch muscles and the effect of PGC-1α

**Figure 3:**
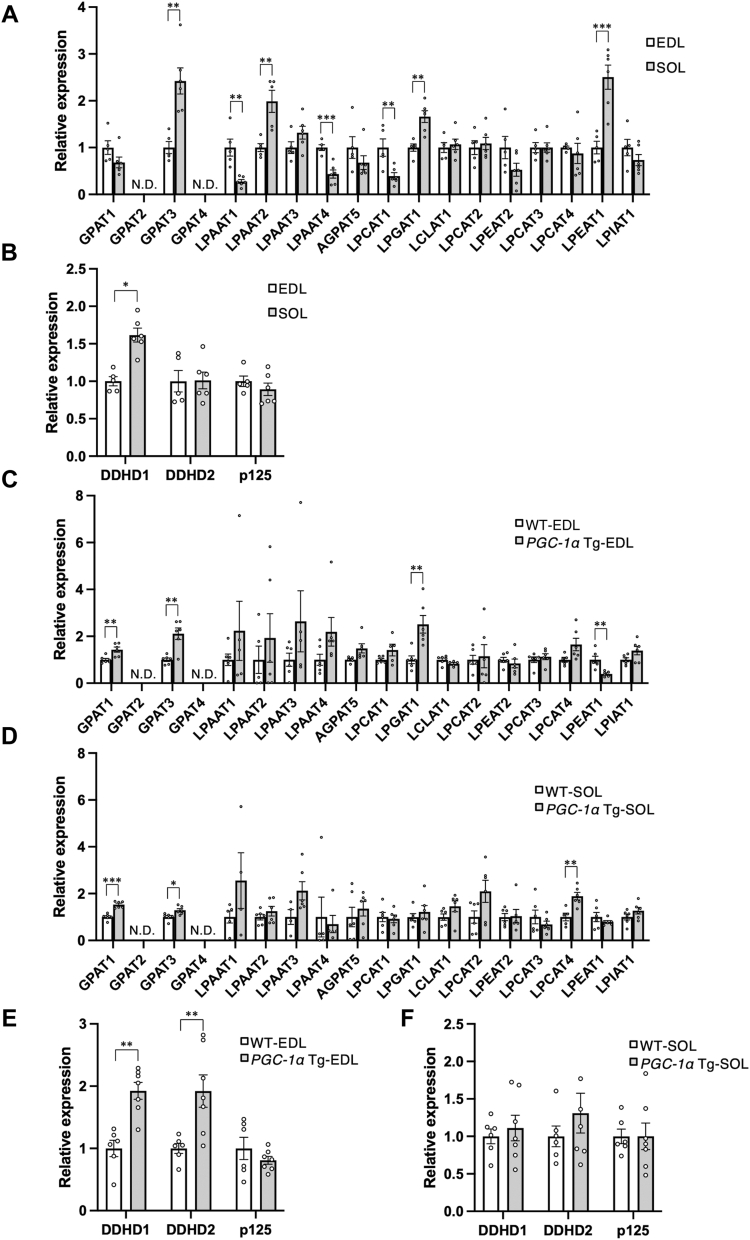
**Expression of genes related to phospholipid biosynthesis and remodeling in the EDL and soleus muscles, and PGC-1α-mediated changes.** Quantitative reverse transcription-polymerase chain reaction (qRT-PCR) analysis of acyltransferase (*A*) and phospholipase A_1_ (*B*) in the EDL and SOL muscles of C57BL/6J mice. Values are represented as the mean ± SEM (n = 5–6). ∗*p* < 0.05; ∗∗*p* < 0.01; ∗∗∗*p* < 0.001; N.D., not detected. *C*–*F*, results of qRT-PCR analysis of acyltransferases (*C* and *D*) and phospholipase A_1_ (*E* and *F*) in the EDL (*C* and *E*) and SOL (*D* and *F*) of WT and *PGC-1α* Tg mice. Values are represented as the mean ± SEM (n = 5–6). ∗*p* < 0.05; ∗∗*p* < 0.01; ∗∗∗*p* < 0.001; N.D., not detected.

Because glycerophospholipids are biosynthesized in the *de novo* pathway and change the acyl chain mainly *via* the remodeling pathway, the muscle fiber type–specific acyl chain profiles of phospholipids may be regulated by multiple factors, including the expression of acyltransferases and phospholipases in addition to acyl-CoA availability. To partly verify this possibility, we measured the mRNA levels of acyltransferases, such as glycerol-3-phosphate acyltransferase (*GPAT*)1 to 4, lysophosphatidic acid acyltransferase (*LPAAT*)1 to 4, acylglycerophosphate acyltransferase (*AGPAT*)5, lysophosphatidylcholine acyltransferase (*LPCAT1*)1 to 4, lysophosphatidylglycerol acyltransferase (*LPGAT*)1, lysocardiolipin acyltransferase (*LCLAT*)1, lysophosphatidylethanolamine acyltransferase (*LPEAT*)1, and lysophosphatidylinositol acyltransferase (*LPIAT*)1. We also focused on the levels of phospholipase A_1_ genes, such as DDHD domain-containing protein 1 (*DDHD*)*1*, *DDHD2*, and Sec23 interacting protein (*p125*) because the typical muscle fiber-specific differences of acyl chain were found in the *sn*-1 position of PC. The levels of *LPAAT1*, *LPAAT4*, and *LPCAT1* were higher in the EDL than in the soleus muscle (Fig. 3*A*). By contrast, the levels of *GPAT3*, *LPAAT2*, *LPGAT1*, and *LPEAT1* were higher in the soleus than in the EDL muscle (Fig. 3*A*). The expression of phospholipase A_1_ is shown in Figure 3*B*. *DDHD1* expression was higher in the soleus than in the EDL muscle. Figure 3, *C*−*F* shows the effect of PGC-1α on the expression of these enzymes in the skeletal muscle. In the EDL muscle, the levels of *GPAT1*, *GPAT3*, and *LPGAT1* were increased by the overexpression of *PGC-1α* (Fig. 3*C*), while the levels of *GPAT1*, *GPAT3*, and *LPCAT4* increased in the soleus (Fig. 3*D*). Figure 3, *E* and *F* shows the expression of phospholipase A_1_. In the EDL, the levels of *DDHD1* and *DDHD2* were increased by overexpressing *PGC-1α*, whereas they were not altered in the soleus muscles.

### Involvement of LPGAT1 in the production of 18:0-PC and 18:0-PE in C2C12 myotubes and murine skeletal muscle

**Figure 4:**
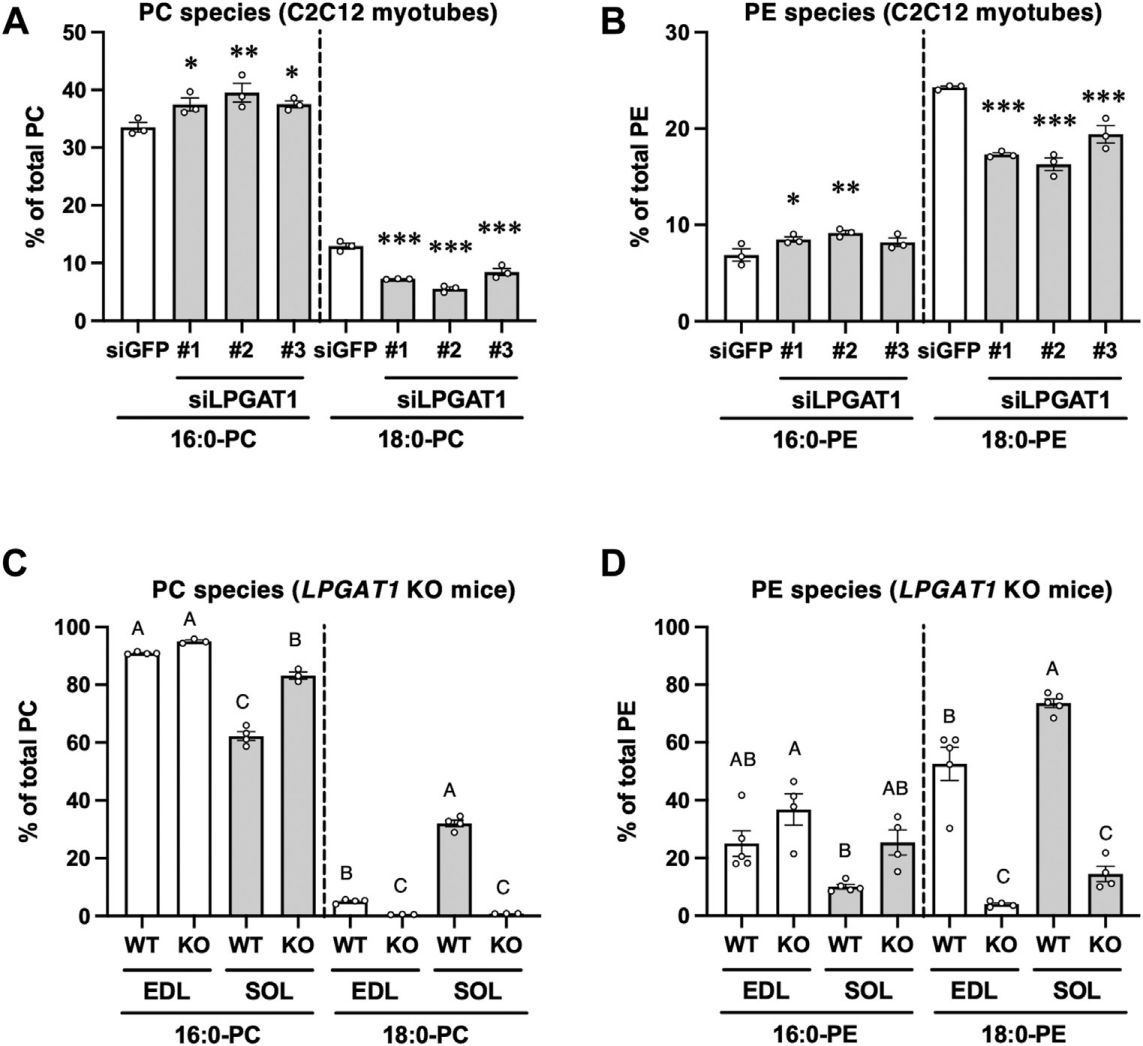
**Changes in the acyl chain profiles of PC and PE in C2C12 myotubes induced by the knockdown of lysophosphatidylglycerol acyltransferase 1 (LPGAT1) or in murine skeletal muscle following global knockout of LPGAT1.** Amounts of 16:0 and 18:0-PC (*A*) and PE (*B*) in siLPGAT1-transfected C2C12 myotubes. siGFP was used as the control. Values are represented as the mean ± SEM (n = 3). ∗*p* < 0.05; ∗∗*p* < 0.01; and ∗∗∗*p* < 0.001 (*versus* values in siGFP-transfected myotubes). Amounts of 16:0 and 18:0-PC (*C*) and PE (*D*) in LPGAT1-deficient (KO) EDL and SOL muscles. Values are represented as mean ± SEM (n = 3–5). Means without a common letter differ significantly (*p* < 0.05).

To reveal the mechanisms for the production of 18:0-PC and 18:0-PE, PC and PE molecular species were analyzed in C2C12 myotubes transfected with siGPAT3, siLPGAT1, and siDDHD1 because the expression levels of these genes were higher in the soleus and *PGC-1α* Tg-EDL than in the EDL and wild-type (WT)-EDL, respectively (Fig. 3). In addition, siLPCAT1 was also introduced because its expression levels were different in the EDL from that in the soleus, and acyltransferase activity and 16:0-CoA selectivity were observed in *LPCAT1*-transfected cells (22). Although siGPAT3 #1, #2, and #3 were effective in reducing *GPAT3* mRNA expression (Fig. S2*A*), the amounts of 16:0-PC and 18:0-PC were not changed by siGPAT3 (Fig. S3*A* and Table S7) and PE profiles were almost unchanged (Fig. S3*B* and Table S8). siLPCAT1 #1, #2, and #3 were effective in reducing *LPCAT1* mRNA expression (Fig. S2*B*). The amount of 16:0-PC was slightly decreased by siLPCAT1 #2, and that of 18:0-PC was slightly increased by siLPCAT1 #1 (Fig. S3*C* and Table S9). The PE profiles remained almost unchanged (Fig. S3*D* and Table S10). siLPGAT1 #1, #2, and #3 were effective in reducing *LPGAT1* mRNA expression (Fig. S2*C*). *LPGAT1* knockdown in C2C12 myotubes caused a significant decrease in the amount of 18:0-PC and an increase in that of 16:0-PC (Fig. 4*A*). The amount of each PC molecule is listed in Table S11. Significant decreases in the amounts of PC (18:0–18:1), PC (18:0–18:2), and PC (18:0–20:4) were observed in the cells treated with siLPGAT1. The PE profiles also changed, and 18:0-PE was decreased by *LPGAT1* knockdown (Fig. 4*B*). The amount of each PE molecule is listed in Table S12. Significant decreases in the amounts of PE (18:0–18:1), PE (18:0–18:2), and PE (18:0–22:6) were observed in the cells treated with siLPGAT1. The knockdown efficiencies of siDDHD1 #1 and #3 were higher than that of siDDHD #2 (Fig. S2*D*). Therefore, for the knockdown of C2C12 myotubes, we used siDDHD1 #1 and a mixture of siDDHD1 #1 and #3. These siDDHD1s reduced *DDHD1* mRNA expression in C2C12 myotubes (Fig. S2*E*). *DDHD1* knockdown caused a decrease in the amount of 18:0-PC in C2C12 myotubes (Fig. S3*E*). The amounts of PC molecules are listed in Table S13. The PE profiles remained almost unchanged (Fig. S3*F* and Table S14).

To investigate the acyltransferase involvement in the production of 18:0-PC and 18:0-PE in murine skeletal muscle, PC and PE molecular species were analyzed in LPCAT1, LPGAT1, and DDHD1-deficient murine skeletal muscle. In the LPCAT1-deficient EDL and soleus muscle, no significant changes were observed in the amounts of 16:0-PC and 18:0-PC compared with those in WT (Fig. S4*A*). The amounts of each PC molecule detected in the LPCAT1-deficient and WT skeletal muscles are shown in Table S15. In contrast, LPCAT1 deficiency caused a decrease in the amount of 16:0-PE and an increase in the amount of 18:0-PE in the EDL muscle (Fig. S4*B*). The amounts of each PE molecule detected in LPCAT1-deficient and WT skeletal muscles are shown in Table S16. The amount of PE (16:0–22:6) decreased in the LPCAT1-deficient EDL. However, the differences observed in LPCAT1-deficient muscles were small. The amounts of 18:0-PC and PE were significantly reduced in the LPGAT1-deficient-EDL and soleus compared with WT (Fig. 4, *C* and *D*). The amounts of each PC and PE molecule detected in the LPGAT1-deficient and WT skeletal muscles are shown in Tables S17 and S18, respectively. PC (18:0–20:4) and PE (18:0–22:6) were decreased in the LPGAT1-deficient EDL and soleus compared with those in the WT EDL and soleus. PC (18:0–18:2), PC (18:0–18:1), and PC (18:0–22:6) amounts decreased, whereas PC (16:0–16:0) and PC (16:0–20:4) amounts increased in the LPGAT1-deficient soleus. By contrast, obvious changes were not observed on knocking out *DDHD1* (Fig. S4, *C* and *D*). The amounts of PC and PE molecules detected in the DDHD1-deficient and WT skeletal muscles are shown in Tables S19 and S20, respectively. These results suggest that LPGAT1 contributes to the production of 18:0-PC and 18:0-PE in murine skeletal muscles.

### Changes in the stearate incorporation into phospholipids of mice lacking LPGAT1 in the skeletal muscle

**Figure 5:**
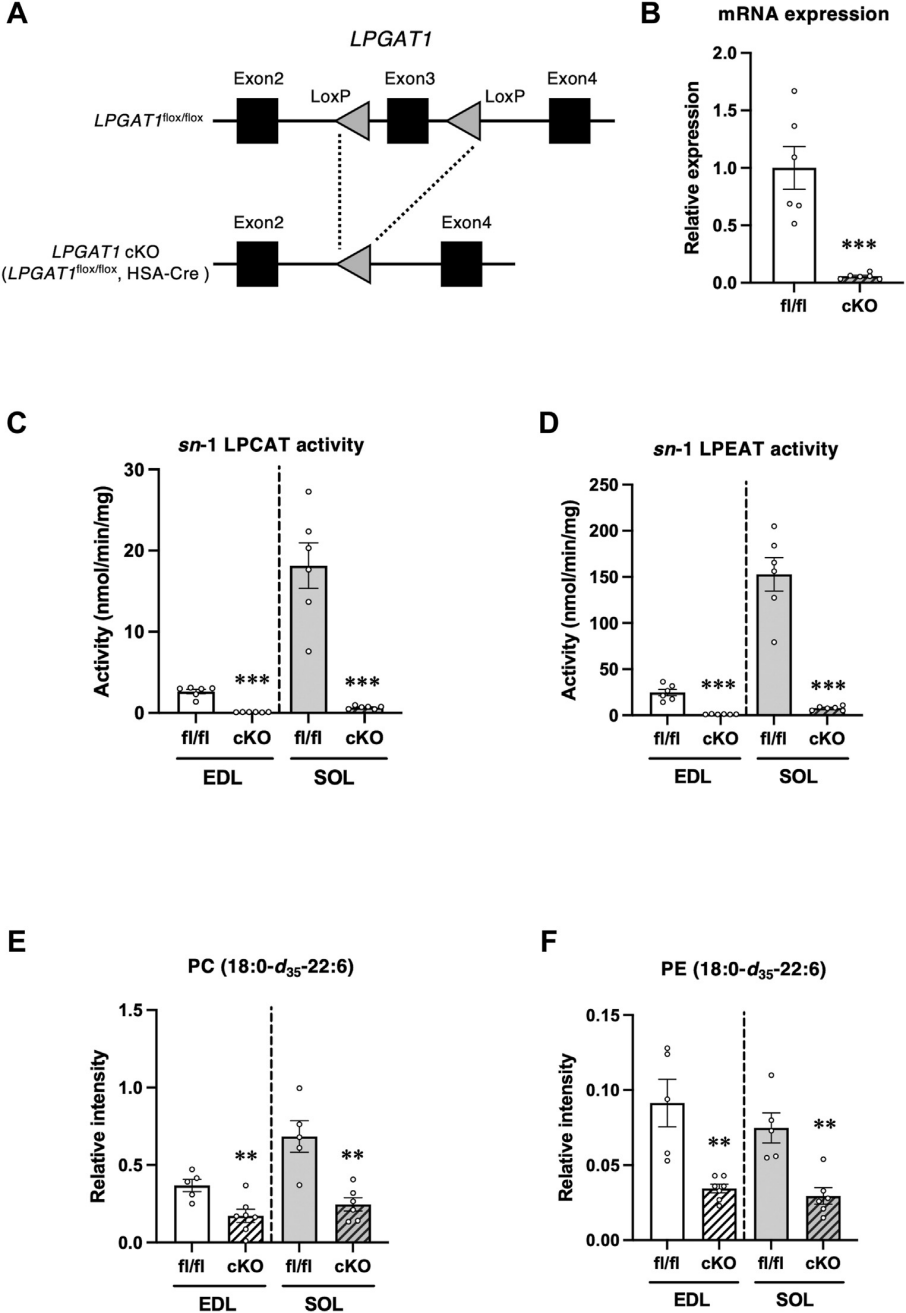

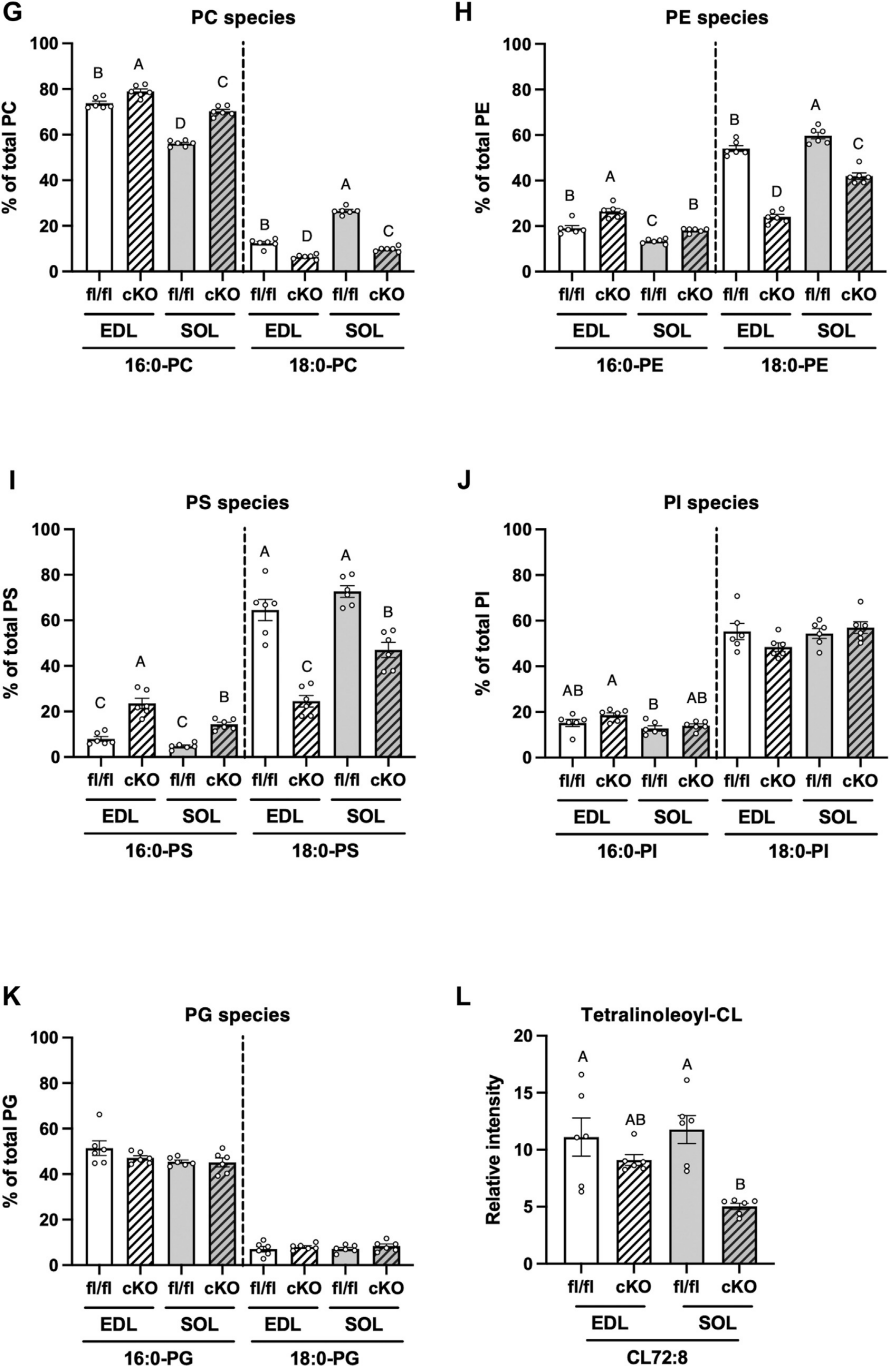
**Changes in acyl chain profiles of phospholipids in mice lacking LPGAT1 in the skeletal muscle.***A*, conditional knockouts use the gene-targeting approach to introduce modifications that include the insertion of loxP sites around Exon 3 sequences within the *LPGAT1*. Floxed mice are crossed with Cre transgenic mice under the control of the HSA promoter (HSA-Cre) to generate *LPGAT1* cKO. *B*, results of qRT-PCR analysis of LPGAT1 mRNA in the gastrocnemius of *LPGAT1*^flox/flox^ mice (fl/fl) and *LPGAT1* cKO mice (cKO). Primers for qRT-PCR targeted deleted regions in *LPGAT1* (Table S29, LPGAT1 Exon 3). Values are represented as the mean ± SEM (n = 6). ∗∗∗*p* < 0.001 (*versus* fl/fl). *C* and *D*, *sn*-1 LPCAT activity and *sn*-1 LPEAT activity in EDL and SOL. The assays were performed using sn-2-rich lyso-PC or lyso-PE as acyl acceptors, stearoyl CoA as acyl donors, and membrane fractions of EDL and SOL as enzyme sources. Values are represented as the mean ± SEM (n = 6). ∗∗∗*p* < 0.001 (*versus* fl/fl). *E* and *F*, EDL and SOL were treated with 1.25 mM of stearic-*d*_35_ acid (18:0-*d*_35_). The levels of PC (18:0-*d*_35_-22:6) (*E*) and PE (18:0-*d*_35_-22:6) (*F*) were measured using LC-MS/MS. The ion intensities of each PC species were normalized against the ion intensities of the internal standard and presented as relative intensity. Values are represented as the mean ± SEM (n = 5−7). ∗∗*p* < 0.01 (*versus* fl/fl). *G*–*K*, amounts of 16:0 and 18:0-PC, PE, PS, PI, and PG in the EDL and SOL muscles of fl/fl and cKO mice. *L*, amount of tetralinoleoyl-CL in the EDL and soleus muscles of fl/fl and cKO mice. Values are represented as the mean ± SEM (n = 6). ∗∗∗*p* < 0.001 (*versus* fl/fl). Means without a common letter differ significantly (*p* < 0.05).

As the productive rate of global *LPGAT1*-knockout (KO) mice was low (23), conditional *LPGAT1* KO (*LPGAT1* cKO) mice were created for further analysis by breeding mice carrying a floxed *LPGAT1* allele (*LPGAT1*^flox/flox^) with mice carrying a Cre recombinase transgene under the control of the human α-skeletal actin (HSA) promoter (HSA-Cre) (Fig. 5*A*). The expression of the exon 3 regions of *LPGAT1* mRNA in the gastrocnemius was reduced by 94.3% in *LPGAT1* cKO mice (Fig. 5*B*).

We investigated whether the skeletal muscle contained endogenous acyltransferase activity that could introduce stearate into lyso-PC and lyso-PE, and these activities were decreased by *LPGAT1*-knockout, *in vitro* and *ex vivo*. For the *in vitro* assay, we prepared lyso-PC and lyso-PE from dioleoyl PC and dioleoyl PE, respectively, using phospholipase A_1_, and stored these lysophospholipids avoiding spontaneous acyl-migration reaction. These prepared lysophospholipids contained a high proportion of *sn*-2 isomers (*sn*-2-rich preparations), such as >94% of 2-oleoyl lyso-PC and >86% of 2-oleoyl lyso-PE, as shown in Fig. S5. Using these *sn*-2-rich preparations as acyl acceptors, stearoyl-CoA as acyl donor, and membrane fraction prepared from skeletal muscle as enzyme sources, acyltransferase activities were measured *in vitro*. Lyso-PC acyltransferase activity, which introduced stearate into the *sn*-1 position of lyso-PC (*sn*-1 LPCAT activity), was higher in the soleus than in the EDL, and these enzyme activities were significantly abolished in *LPGAT1*-KO skeletal muscle (Fig. 5*C*). Lyso-PE acyltransferase activity, which introduced stearate into the *sn*-1 position of lyso-PE (*sn*-1 LPEAT activity), was also higher in the soleus than in the EDL, and these enzyme activities were significantly abolished in *LPGAT1*-KO (Fig. 5*D*). To evaluate the direct incorporation of stearate into PC and PE by LPGAT1, the EDL and soleus excised from *LPGAT1*^flox/flox^ and *LPGAT1*-KO mice were incubated with deuterium-labeled stearate (18:0-*d*_35_). Subsequently, the incorporation of 18:0-*d*_35_ into PC and PE was analyzed using LC-MS/MS. The formation of PC (18:0-*d*_35_-22:6) and PE (18:0-*d*_35_-22:6) was significantly reduced in the *LPGAT1*-KO skeletal muscle (Fig. 5, *E* and *F*).

To clarify whether *LPGAT1* deletion induces phospholipid changes in the EDL and soleus similar to those in global *LPGAT1* KO mice, we measured the levels of phospholipids such as PC and PE. The amount of 18:0-PC in the EDL and soleus decreased in *LPGAT1* cKO mice, whereas the amount of 16:0-PC increased (Fig. 5*G* and Table S21). The amount of 16:0 and 18:0-PE in the *LPGAT1* cKO mice changed similarly to that of 16:0 and 18:0-PC (Fig. 5*H* and Table S22). The amounts of phosphatidylserine (PS), phosphatidylserine (PI), and phosphatidylglycerol (PG) molecules detected in the LPGAT1-deficient skeletal muscles are shown in Tables S23–S25, respectively. The amounts of 16:0-PS and 18:0-PS in the EDL and soleus were also altered by knocking out *LPGAT1* (Fig. 5*I* and Table S23). The effect of muscle *LPGAT1* deletion on 16:0-PI and 18:0-PI was not observed in the EDL and soleus (Fig. 5*J* and Table S24). Although LPGAT1 has been reported to be involved in the remodeling of the acyl chains at the *sn*-2 position of PG and prefers palmitoyl-CoA, stearoyl-CoA, or oleoyl-CoA as acyl donors (24), the relative amount of 16:0-PG and 18:0-PG was not changed in the LPGAT1-deficient skeletal muscle compared with that in the control muscle (Fig. 5*K* and Table S25). Slight changes were observed in 18:1-PG and 18:2-PG in the soleus and EDL, respectively (Fig. S6 and Table S25). Tetralinoleoyl-cardiolipin (CL), a major CL molecule in the skeletal muscle, was decreased in the LPGAT1-deficient soleus; however, LPGAT1 deficiency-induced changes in tetralinoleoyl-CL were not observed in the EDL (Fig. 5*L*).

## Discussion

Phospholipids are important structural components of cellular membranes. Over 1000 molecular species of glycerophospholipids are speculated to exist in the body as a combination of acyl chain and polar heads. This diversity of phospholipids controls membrane fluidity and membrane protein function and affects cellular function (25). Recently, it has become clear that changes in phospholipid profiles due to diseases are closely associated with their pathology and function (22, 26). The skeletal muscle consists of both fast- and slow-twitch fibers, which differ in their contractile and metabolic characteristics. Although differences in the fatty acid species in phospholipid fractions are related to skeletal muscle fiber type (10, 17, 18), the differences in the intact phospholipid molecules and the mechanisms behind those differences are still not fully understood. Therefore, to elucidate the control mechanisms of myofiber type–specific acyl chain profiles of phospholipids, PC and PE molecular species were comprehensively analyzed in the EDL, soleus, and *PGC-1α*-overexpressing skeletal muscles that served as a model of acquired slow-twitch muscle. This study revealed that the acyl chain profiles of phospholipids vary greatly depending on the muscle fiber types and that fast-twitch muscles are mainly composed of 16:0-PC species; slow-twitch muscles are composed of not only 16:0-PC but also 18:0-PC species. These differences were characterized by the acyl chain species at the *sn*-1 position of phospholipids. Although these results agree with those from previous reports (10, 17, 18), the present study is the first to find that differences in acyl chain profiles of phospholipids among muscle fiber types are characterized by the acyl chain species at the *sn*-1 position of phospholipids. Furthermore, we investigated the control mechanisms of muscle fiber type-specific acyl chain profiles of phospholipids by analyzing cultured cells treated with small-interfering RNAs (siRNAs) and genetically modified murine skeletal muscles. In this study, it is suggested that muscle fiber type-specific phospholipid profiles are controlled by LPGAT1, an acyltransferase that re-incorporates acyl-CoA into phospholipid molecules *via* the remodeling pathway. As overexpression of *PGC-1α* increased the expression of *LPGAT1* mRNA and the amount of 18:0-PC in the EDL concomitant with fiber type switching from fast to slow-twitch muscle, LPGAT1-induced change of acyl chain profiles in muscular phospholipids might be involved in PGC-1α-mediated enhancement of endurance capacity.

### Remodeling of the acyl chain at the *sn*-1 position of phospholipids by LPGAT1

Replacement of the acyl chain at the *sn*-2 position of phospholipids occurs after hydrolysis by phospholipase A_2_. These replacement reactions are called “remodeling,” and lysophospholipid acyltransferases (LPLATs) control these reactions (27, 28, 29, 30, 31). It has recently become clear that remodeling of the acyl chain also occurs at the *sn*-1 position of phospholipids. For instance, murine LCLAT1 remodeled the acyl chain at the *sn*-1 position in *Caenorhabditis elegans*, mice, and yeast (32, 33, 34). DDHD1, identified as phospholipase A_1_, has been reported to be involved in producing 2-arachidonoyl-1-lyso-phospholipid (35). In the DDHD1-deficient brain, the amount of polyunsaturated fatty acid (PUFA)-containing lysophospholipids was greatly reduced, and the phospholipid profile changed drastically (36).

LPGAT1, which is now proposed to be termed LPLAT7 (37), was previously reported as an acyltransferase localized in the endoplasmic reticulum that incorporates long-chain fatty acyl-CoA, such as palmitoyl-CoA, stearoyl-CoA, and oleyl-CoA, into 1-acyl-2-lyso-PG *in vitro* (24). Loss of *LPGAT1* expression in the murine liver resulted in a significant decrease in the levels of PG (16:0–18:2), PG (18:2–18:2), PG (18:1–18:1), and CL (18:2)_4_ (38). In addition to PG and CL, Zhang *et al.* (38) showed that the livers from global *LPGAT1*-KO mice significantly altered their acyl chain compositions in PC, PE, PS, PA, and PI. They showed that compared with those in WT mice livers, the amounts of 18:0-PC, 18:0-PE, and 18:0-PS were decreased in the liver of *LPGAT1*-KO mice (38), as observed in the skeletal muscles of *LPGAT1*-cKO mice in the present study. These results suggest that LPGAT1 is involved in remodeling the acyl chain in PC, PE, and PS *in vivo*, in addition to the previously reported function. During the preparation of this article, three papers were published reporting that there is a significant decrease in the levels of 18:0-PC and 18:0-PE and an increase in the levels of 16:0-PC and 16:0-PE in *LPGAT1* mutant cells, zebrafish, and tissues from *LPGAT1*-KO mice (23, 39, 40). Furthermore, Kawana *et al.* (23) suggested that LPGAT1 was an *sn*-1-specific LPLAT for lyso-PC, lyso-PE, and lyso-PS and contributed to the production of 18:0-PC, PE, and PS by the acyl chain remodeling of these phospholipids. Our results were consistent with the conclusions from these recent studies, which indicated that LPGAT1 was involved in the production of 18:0-PC, PE, and PS in the skeletal muscle.

### Regulation of LPGAT1 expression by PGC-1α

The transcription factors that control the expression of *LPGAT1* remain unknown. In the present study, the expression of *LPGAT1* was higher in slow-twitch muscles than in fast-twitch muscles and increased by the expression of *PGC-1α*. Therefore, it has been suggested that the expression of *LPGAT1* is controlled by transcription factors activated by *PGC-1α*. Using the MotifMap online database (http://motifmap.ics.uci.edu/) (41), a simulated promoter analysis of *LPGAT1* was performed. It was shown that the binding sequence for myocyte enhancer factor 2A (MEF2A) existed 1.75 kb upstream from the transcription start site of *LPGAT1*. Because the transcriptional activity of the MEF2 family is activated by PGC-1α (42), transcription of *LPGAT1* was suggested to be promoted *via* MEF2A in skeletal muscles overexpressing *PGC-1α*. Furthermore, MEF2 activation has been reported to promote the formation of slow-twitch myofibers and enhance running endurance (43). The MEF2 family proteins enhance the expression of slow-twitch and oxidative fiber-specific genes, such as troponin I (slow), myoglobin (44), carnitine palmitoyltransferase-1β (45), and *PGC-1α* (42). The elevated expression of *LPGAT1* in slow-twitch muscle might be explained by MEF2 family-dependent gene expression. In future studies, to investigate whether the MEF2 family is involved in the transcription of *LPGAT1* and whether PGC-1α enhances its transcriptional activity, it is necessary to measure the transcriptional activity of the MEF2 family proteins in the expression of *LPGAT1* using a reporter gene assay.

### Biological functions of LPGAT1

Several reports have shown the biological functions of LPLATs. For instance, LPCAT1 controls respiratory function *via* the biosynthesis of PC (16:0–16:0) (22). LPCAT3 maintains intestinal function through the biosynthesis of 18:2 and 20:4-PC (27). LPIAT1 is involved in the biosynthesis of 20:4-PI and plays an important role in brain development (46). The physiological significance of LPGAT1 has also been reported. For instance, LPGAT1 is important in hepatic triacylglycerol secretion into circulation (47). Loss of whole-body LPGAT1 protects mice from diet-induced obesity but leads to hepatopathy, insulin resistance, and nonalcoholic fatty liver diseases due to oxidative stress, mitochondrial DNA depletion, and mitochondrial dysfunction (38). Single-nucleotide polymorphisms of LPGAT1 influence the body mass index in Native Americans (48) and are strongly correlated with hypo-HDL-cholesterolemia (49). These findings suggest that LPGAT1 is associated with lipid metabolism. As slow-twitch muscles and skeletal muscles overexpressing *PGC-1α* preferentially utilize lipids as a source of energy and have high lipid metabolic capacity (7, 50), changes in the phospholipid profiles *via* LPGAT1 may play an important role in the lipid metabolism of slow-twitch muscle.

### Acyl chain profiles of phospholipid and its physiological significance

Our findings suggest that 18:0-PC and 18:0-PE play important roles in slow-twitch muscle function. The thickness of the biological membrane is determined by the type of acyl chain present in the phospholipids. Longer acyl chains of saturated fatty acid (SFA) thicken the membrane, and fatty acids with more double bonds lead to thinner membranes (25, 51). For example, biological membranes that possess PUFA-containing phospholipids, such as 22:6, are thinner than those possessing SFA-containing phospholipids (52). Therefore, it is expected that in the case of phospholipid molecules that contain SFA at the *sn*-1 position and PUFA at the *sn*-2 position, which are the most common molecules in the skeletal muscle, the length of SFA at the *sn*-1 position determines the thickness of the biological membrane. In the skeletal muscle, most palmitate and stearate were bound to the *sn*-1 position of 16:0- and 18:0-PC, respectively, and 18:0-PC was found in type I and IIa fibers, suggesting that the biological membranes of the fast-twitch muscle are thin, while some parts of the membrane of the slow-twitch muscle are thick. The thickness of biological membranes has been reported to affect the function of membrane proteins. For instance, bovine heart mitochondrial cytochrome c oxidase (COX) activity incubated with 18:0-PC was approximately double that of 16:0-PC (53). It was assumed that 18:0-PC might enhance COX activity and ATP production in the mitochondria of slow-twitch muscle. Further studies are needed to fully explain the functional difference between 16:0- and 18:0-containing phospholipids in the skeletal muscle.

In conclusion, this study revealed that phospholipid profiles varied greatly depending on the muscle fiber type, and the difference was characterized by the acyl chain species at the *sn*-1 position of PC. Moreover, it was suggested that the differences in the muscle fiber type-specific acyl chain profiles of PC and PE were controlled by the expression of LPGAT1, an acyltransferase involved in the remodeling pathway. In this study, we clarified some of the control mechanisms of myofiber type-specific acyl chain profiles of phospholipids and showed the regulation of the acyl chain profiles of phospholipids by LPGAT1 in skeletal muscle.

The study, however, has a limitation. As it is well-recognized that a product ion mass spectrum of PUFA such as 22:6 acquired in the negative ion mode displays the characteristic fragment ion [M – H – 44]^−^ corresponding to the facile neutral loss of CO_2_ from the molecular ion (54), the amounts of PUFA-containing phospholipids quantified by MRM in negative ionization mode are possibly underestimated.

## Experimental procedures

### Experimental animals

Male C57BL/6J mice (9–10-week-old) obtained from Japan SLC Inc were used for all experiments. The methods for generating *PGC-1α* Tg mice have been previously described (6). The promoter for HSA provided by Drs E. C. Hardeman and K. L. Guven (Children’s Medical Research Institute) was used to express *PGC-1α-b* in skeletal muscle. Transgenic mice (heterozygotes, BDF one background) and WT C57BL/6J mice were crossed several times. Female 10 to 13-week-old offspring (heterozygote and WT, from the same litter) were used for the experiments. To generate mice with *PGC-1α* knocked out specifically in the skeletal muscles (*PGC-1α*-KO mice), we inactivated *PGC-1α* expression in skeletal muscles by crossing mice carrying a floxed *PGC-1α* allele with mice transgenic for the human α-skeletal actin promoter driven-Cre transgenic. *PGC-1α*^flox/flox^ mice were obtained from the Jackson Laboratory (12). Male *PGC-1α*-KO mice, 14 weeks old, were used for the experiments. Global *LPCAT1*-KO mice (9-week-old, male) and *DDHD1*-KO mice (7–11-week old, female) were prepared as previously described (22, 55). *LPGAT1*-KO mice (MMRRC Stock No: 42167-JAX) were obtained from Jackson Laboratory. Fourteen-week-old male *LPGAT1*-KO mice were used in each experiment. To generate *LPGAT1* cKO mice, *LPGAT1* expression was inactivated by crossing mice carrying a floxed *LPGAT1* allele with Cre transgenic mice under the control of the HSA promoter. *LPGAT1*^flox/flox^ mice were obtained from Cyagen Biosciences. To prepare an *LPGAT1* cKO mouse model using CRISPR/Cas-mediated genome engineering, exon 3 of *LPGAT1*, located on mouse chromosome 1, was selected as the conditional KO region.

Mice were maintained in a 12 h light/dark cycle at 22 °C and fed a normal chow diet (CE-2; Clea Japan) *ad libitum*. Mice were cared for in accordance with the National Institutes of Health Guide for the Care and Use of Laboratory Animals and our institutional guidelines. All animal experiments were conducted with the approval of the Institutional Animal Care and Use Committee of the University of Shizuoka (number 165122). The mice were sacrificed by cervical dislocation, and tissue samples were collected. The samples for measuring phospholipids and mRNA expression were snap-frozen in liquid nitrogen and stored at −80 °C until analysis.

### Analysis of PC and PE in skeletal muscles

Lipids were extracted from the EDL and soleus muscles. Frozen muscles were homogenized and powdered in liquid nitrogen. After adding the corresponding internal standards, total lipids were extracted overnight from the homogenates with 1 ml chloroform/methanol (2:1, v/v with 0.2 mg/ml butyl hydroxyl toluene). The extracts were evaporated to dryness under vacuum. Samples were reconstituted in an equal volume of acetonitrile/isopropanol/H_2_O (65:30:5, v/v/v). After ultrafiltration, the filtrate of the muscle sample was diluted ten times, and 10 μl of the sample was injected into the LC-MS system.

The PC and PE species were analyzed using an LCMS-8040 triple quadrupole mass spectrometer (Shimadzu) equipped with an electrospray source ionization probe, LC-30AD binary pump (Shimadzu), SIL-30AC auto sampler (Shimadzu), and CTO-20AC column oven (Shimadzu). Accucore RP-MS column (2.6 μm, 2.1 mm × 50 mm; Thermo Fisher Scientific) was used for high-performance liquid chromatography (HPLC) analysis. Mobile phase A consisted of H_2_O/acetonitrile (60:40, v/v), and mobile phase B consisted of isopropanol/acetonitrile (90:10, v/v). Mobile phases A and B were supplemented with 10 mM ammonium formate and 0.1% formic acid. The flow rate was 0.35 ml/min. The gradient was as follows: 40% B at 0 min, 40% B at 2 min, 52% B at 8 min, 60% B at 20 min, 100% B at 25 min, and 40% B at 30 min. For MS/MS analysis, the nebulizer gas flow was set to 3.0 l/min, the drying gas flow was set to 15.0 l/min, the desolvation line temperature was set to 250 °C, the heat-block temperature was set to 400 °C, and the collision-induced dissociation (CID) of gas was set to 230 kPa. Details of the scanning modes are listed in Table S26.

To identify the phospholipid species using the LIPID MAPS online MS tool, MS/MS data obtained for individual target peaks were searched against a database of glycerophospholipids (http://www.lipidmaps.org/tools/ms/GP_prod_search.html) precursor/product ions.

The obtained peak area of each individual species was normalized against the peak area of internal standard and muscle weight. The peak areas of each individual species were normalized against the sum of all peak areas within each phospholipid class to determine their abundance (expressed as a percentage of the total). The amount of PC (14:0–16:0) was not included in the amounts of PC molecules containing 16:0 (16:0-PC). The detected peaks were aligned according to the *m/z* value and the normalized retention time using Signpost MS (Reifycs).

### Incorporation of deuterium-labeled fatty acids into PC and PE in excised skeletal muscle

For the experiment whose results are shown in Figure 1, *C* and *D*, EDL and soleus muscles were placed immediately after dissection in a 20 ml glass reaction vial containing 2 ml of warmed (30 °C), pre-gassed (95% O_2_, 5% CO_2_, pH 7.4), modified Krebs–Henseleit buffer containing 4% fatty acid-free bovine serum albumin (013-25773; Wako Pure Chemical), and 5 mM glucose. Thereafter, these muscle samples were incubated with 0.625 mM deuterium-labeled palmitic acid (16:0-*d*_31_) or stearic acid (18:0-*d*_35_) (Cambridge Isotope Laboratories, Inc) in the presence of 0.625 mM non-labeled stearic acid (18:0) or palmitic acid (16:0) for 2 h, respectively. For the experiment whose results are shown in Figure 5, *E* and *F*, the EDL and soleus from *LPGAT1*^flox/flox^ or *LPGAT1* cKO mice were incubated in the modified Krebs–Henseleit buffer containing 1.25 mM deuterium-labeled stearic acid (18:0-*d*_35_). After incubation, these muscles were placed in liquid nitrogen and powdered using a pestle. Lipid extraction and LC-MS analysis were performed as described in the above section, “Analysis of PC and PE in skeletal muscles.” PC (17:0–17:0) and PE (12:0–12:0) (Avanti Polar Lipids) added to chloroform/methanol were used as the internal standard. The MRM conditions are listed in Table S27. The obtained peak area of PC (16:0-*d*_31_-22:6), PC (18:0-*d*_35_-22:6), and PE (18:0-*d*_35_-22:6) were normalized against the peak area of the internal standard and muscle weight.

### Imaging-MS

The tissue blocks (gastrocnemius) were rapidly frozen using isopentane cooled with liquid nitrogen. Transverse cross-sections of 10 μm were made using a cryostat (CM1860; Leica, Wetzlar, Germany) at −25 °C. Cryosections were attached to indium-tin-oxide (ITO)-coated glass slides (Bruker Daltonics). The prepared tissues were coated with 2,5-dihydroxybenzoic acid as the matrix (50 mg/ml dissolved in 80% ethanol) by manual spraying with an artistic brush (Procon Boy FWA Platinum; Mr Hobby). MALDI imaging was performed using an Ultraflextreme MALDI-time of flight (TOF) mass spectrometer (Bruker Daltonics). Data were acquired in the positive ion mode with raster scanning at a pitch distance of 10 μm. Each spectrum was the result of 2000 laser shots at each data point. In this analysis, signals between *m/z* 400 and 1200 were collected. Image reconstruction was performed using FlexImaging v.4.1 software (Bruker Daltonics).

### Immunofluorescence imaging

Acetone-fixed 10 μm cryosections of gastrocnemius were rinsed in phosphate-buffered saline with 1% Tween 20 (PBS-T). After washing with PBS-T, the tissue sections were blocked with blocking mouse IgG (Vector Laboratories) and 5% normal goat serum (Thermo Fisher Scientific). The sections were incubated with anti-myosin heavy chain (MHC) I (1:250), anti-MHC IIa (1:250), anti-MHC IIb (1:250) (BA-F8, SC-71, BF-F3; Developmental Studies Hybridoma Bank), and anti-laminin (1:500; Sigma Chemical) antibodies overnight at 4 °C. The following day, the sections were rinsed with PBS-T and incubated with the appropriate secondary antibody conjugated to Alexa Fluor 350, 488, 555, and 647 antibodies, respectively (A21140, A21042, A21127, A21244; Thermo Fisher Scientific). Slides were mounted with ProLong Gold Antifade Mountant (P36930; Thermo Fisher Scientific) and glass coverslips. Fluorescent images were captured using a DMi8 inverted microscope (Leica).

### Analytical conditions for identification of PC positional isomers

Lipids were extracted from the gastrocnemius muscle. Samples were prepared as described above. Synthetic PC (16:0/22:6) and PC (18:0/22:6) were purchased from Avanti Polar Lipids. PC positional isomers were analyzed using an LCMS-8040 triple quadrupole mass spectrometer (Shimadzu) with an electrospray source ionization probe. ACQUITY UPLC CSH C18 column (1.7 μm, 2.1 × 150 mm; Waters) was used for HPLC analysis. Mobile phase A consisted of acetonitrile/water (90:10, v/v), and mobile phase B consisted of acetonitrile/methanol/isopropanol/water (47.5:45:2.5:5, v/v/v/v). Both mobile phases were supplemented with 15 mM ammonium formate and adjusted to pH 7.4 by adding aqueous ammonium ions. The flow rate and the column temperature were set to 0.11 ml/min and 30 °C, respectively. The gradient was as follows: 0% B at 0 min, 100% B at 5 min, 100% B at 70 min, 0% B at 70.1 min, and 0% B at 85 min. For MS/MS analysis, the nebulizer gas flow was set to 3.0 l/min, the drying gas flow was set to 15.0 l/min, the desolvation line temperature was set to 250 °C, the heat-block temperature was set to 400 °C, and the CID gas was set to 230 kPa. MRM and scanning conditions are listed in Table S28.

### Quantitative reverse transcription PCR

Total RNA was extracted using RNAiso Plus (9108; Takara Bio Inc). Reverse transcription (RT) was performed with PrimeScript RT reagent Kit with gDNA Eraser (RR047A, Takara Bio Inc) conducted on 1 μg of RNA. Real-time PCR was performed using TB Green Premix Taq II (RR820S, Takara Bio Inc). RNA extraction, RT, and real-time PCR were performed according to the manufacturer’s protocol. Primer sequences are listed in Table S29. The expression of target genes was normalized against the expression of the housekeeping gene 36B4 using the standard curve method. mRNA expression in the *PGC-1α* Tg mice was re-evaluated using the cDNA reported in a previous paper (12) with the primers shown in Table S29. The amount of LPGAT1 mRNA in *LPGAT1* cKO mice was measured using LPGAT1 Exon 3 primers (Table S29) and normalized against the expression of the housekeeping gene 18S rRNA.

### C2C12 cell culture

C2C12 cells were cultured at 37 °C in a humidified atmosphere containing 5% CO_2_. C2C12 cells were passaged in a growth medium consisting of DMEM (high glucose, with L-glutamine and phenol red, 044-29765, Wako Pure Chemical) supplemented with 10% heat-inactivated (56 °C, 30 min) fetal bovine serum (SH30088.03; GE Healthcare UK Ltd). To induce differentiation, the cell medium was replaced with a differentiation medium consisting of DMEM containing 2% heat-inactivated horse serum (26050070; Thermo Fisher Scientific). The differentiation medium was replenished every other day for 6 days on 12-well Corning tissue culture plates, which had been coated with 0.1% gelatin. The differentiation medium was supplemented with a fatty acid mixture comprising 5 μM each of 18:2, 20:4, and 22:6.

### siRNA transfection of C2C12 cells

siRNAs targeting murine LPGAT1, LPCAT1, GPAT3, DDHD1, and control GFP were purchased from Japan Bio Services (Saitama, Japan). siRNAs were designed using siDirect siRNA design site (http://sidirect2.rnai.jp/), and the sequences are shown in Table S30. Undifferentiated C2C12 cells cultured to confluence were transfected using RNAiMAX transfection reagent (Thermo Fisher Scientific) in 12-well Corning tissue culture plates coated with 0.1% gelatin, and 3 μl RNAiMAX and 10 pmol siRNA were applied per well. siRNA transfection was performed thrice on days 0, 2, and 4 from the start of differentiation.

### Analysis of PC and PE in C2C12 myotubes and LPGAT1 cKO mice

Differentiated C2C12 myotubes were collected in PBS. The samples were centrifuged at 1200*g* for 10 min at 4 °C, and the supernatants were discarded. The pellets were sonicated in 0.5 ml PBS using SONIFIER 250 (Emerson Electric Co). Total lipids were extracted by adding 10 μl of 0.1 mg/ml PC (17:0/17:0), 200 μl of methanol with 0.2 mg/ml butyl hydroxyl toluene, and 100 μl of chloroform to 100 μl of the sample. After vortexing, 100 μl of chloroform and 100 μl of H_2_O were added. The lipophilic phase, which contains the total lipids extracted from C2C12 cells, was sampled. Lipid extraction from murine tissues and reconstitution were performed as described above. The sample (10 μl) was injected into the LC-MS/MS system. PC and PE were quantified using MRM in negative ionization mode, in which detection of fatty acid fragments was set for Q3. LC-MS/MS conditions were the same as described above. The gradient conditions were as follows: 10% B at 0 min, 10% B at 2 min, 100% B at 15 min, 10% B at 15.01 min, and 10% B at 20 min. The MRM conditions are listed in Tables S31 and S32. The amount of PC (16:1–18:0) was neither included in the amounts of 16:0-PC nor in those of 18:0-PC. The amount of PE (16:1–18:0) was not included either in the amounts of 16:0-PE or in those of 18:0-PE. The amounts of PC (16:0–18:0) and PE (16:0–18:0) was counted as 16:0-PC and 16:0-PE, respectively.

### Analysis of other glycerophospholipids in skeletal muscle

Lipid extraction and reconstitution were performed as described above. Twenty microliters of the sample were injected into the LC-MS/MS system. LC-MS/MS conditions were the same as described above. The gradient conditions for PS, PG, and PI analysis were as follows: 10% B at 0 min, 10% B at 2 min, 100% B at 15 min, 10% B at 15.01 min, and 10% B at 20 min. PS, PI, and PG were analyzed using MRM in negative ionization mode, in which detection of fatty acid fragments was set for Q3. The MRM conditions are listed in Tables S33–S35. The obtained peak area of each individual species was normalized against the peak area of the internal standard and muscle weight. The peak areas of each individual species were normalized against the sum of all peak areas within each phospholipid class to determine their abundance (expressed as a percentage of the total). The PS, PG, and PI containing (16:1–18:0) were not included in the amounts of 16:0-PS, PG, and PI, nor 18:0-PS, PG, and PI. The PS, PG, and PI containing (16:0–18:0) amounts were counted as 16:0-PS, PG, and PI, respectively.

CL was extracted overnight from homogenates with 1 ml chloroform/methanol (2:1, v/v with 0.2 mg/ml butyl hydroxyl toluene). Tetramyristoyl-CL (Avanti Polar Lipids) was added to chloroform/methanol as an internal standard. The extracts were evaporated to dryness under a vacuum. Samples were reconstituted in an equal volume of acetonitrile/isopropanol/H_2_O (29:55.8:15.2, v/v/v). After ultrafiltration, 40 μl of the filtrated sample was injected into the LC-MS system. The flow rate was 0.4 ml/min. The gradient condition for tetralinoleoyl-CL analysis was as follows: 40% B at 0 min, 40% B at 2 min, 100% B at 15 min, 100% B at 25 min, and 40% B at 30 min. Tetralinoleoyl-CL was quantified using MRM in negative ionization mode, in which detection of fatty acid fragments was set for Q3. The MRM conditions are listed in Table S36. The peak area of tetralinoleoyl-CL was normalized against the peak area of the internal standard and muscle weight.

### Preparation of membrane fractions for acyltransferase assay

The EDL and soleus were rinsed with ice-cold saline. Rinsed muscles were minced in ice-cold TSC buffer (20 mM Tris-HCl (pH 7.4), 300 mM sucrose, and protease inhibitor cocktail (Roche)). Minced mouse tissues (20–100 mg) were homogenized in ice-cold TSC buffer using a grass homogenizer (Tenbroeck Tissue Grinder), and homogenized tissues were sonicated for three cycles, four times for 10 s each using a probe sonicator (Microtec). After the homogenates were centrifuged at 800*g* for 10 min, the supernatants were centrifuged at 100,000*g* for 1 h. The resultant pellets were re-suspended in TSE buffer (20 mM Tris-HCl (pH 7.4), 300 mM sucrose, and 1 mM EDTA). After the BCA protein assay (Thermo Fisher Scientific), the resulting membrane fractions were stored at −80 °C until further use in the lyso-PC and lyso-PE acyltransferase assay.

### sn-1 LPCAT activity and sn-1 LPEAT activity assay

The acyltransferase activities were determined as previously described (23). Briefly, lyso-PC and lyso-PE solution, prepared from dioleoyl PC and dioleoyl PE using phospholipase A_1_, were dried up in a glass tube. The assay mixtures containing 100 mM Tris-HCl (pH 7.4), 0.03% Tween-20, and acyl-CoAs were added to the tube, and the components were suspended by vortexing and sonicating. The reaction was initiated by adding a membrane fraction prepared from the EDL or soleus and incubated at 37 °C for 10 min. Reactions were terminated by the addition of chloroform-methanol (1:2, v/v). After adding the corresponding internal standards, lipids were extracted using the Bligh and Dyer method. Extracted phospholipids were analyzed using LC-MS/MS.

### Statistical analysis

Data were analyzed using Student’s *t* test (for comparisons between two groups) and one-way ANOVA (for comparisons of three or more groups) followed by Tukey’s HSD test (JMP ver. 11). Values are presented as mean ± standard error of the mean.

## Data availability

Data included or supporting the findings in this article will be shared upon reasonable request by the corresponding author.

## Supporting information

This article contains supporting information.

## Conflict of interest

The authors declare that they have no known competing financial interests or personal relationships that could have appeared to influence the work reported in this paper.
