# Supplementary material for: LPGAT1/LPLAT7 regulates acyl chain profiles at the sn-1 position of phospholipids in murine skeletal muscles
Source: J Biol Chem. 2023 May 20;299(7):104848. doi: 10.1016/j.jbc.2023.104848 (PMC10285227; doi:10.1016/j.jbc.2023.104848)
Supplement: Supplemental Tables [file mmc1.docx]

**Supplementary Information**

**Supplementary Table S1. Phosphatidylcholine profiles of the extensor digitorum longus (EDL) and soleus (SOL) muscles**

|  |  |  | % of total PC | | | | | |
| --- | --- | --- | --- | --- | --- | --- | --- | --- |
| FA composition | Retention time (min) | *m/z* | EDL | | | SOL | | |
| 14:0-16:0 | 9.73 | 706.48 | 0.16 | ± | 0.02 | 0.05 | ± | 0.02** |
| 16:0-16:1 | 10.07 | 732.52 | 1.03 | ± | 0.03 | 0.26 | ± | 0.04*** |
| 16:0-16:0 | 11.74 | 734.55 | 1.48 | ± | 0.08 | 1.54 | ± | 0.14 |
| 16:0-18:2 | 10.49 | 758.52 | 4.52 | ± | 0.13 | 2.77 | ± | 0.21*** |
| 16:0-18:1 | 12.02 | 760.55 | 3.03 | ± | 0.12 | 2.46 | ± | 0.47 |
| 14:0-22:6 | 8.29 | 778.48 | 0.15 | ± | 0.03 | 0.10 | ± | 0.01 |
| 16:0-20:4 | 10.35 | 782.51 | 5.66 | ± | 0.67 | 1.12 | ± | 0.13*** |
| 36:3 | 10.92 | 784.54 | 0.24 | ± | 0.06 | 0.49 | ± | 0.10* |
| 18:0-18:2 | 12.64 | 786.55 | 0.59 | ± | 0.05 | 1.57 | ± | 0.13*** |
| 18:0-18:1 | 14.53 | 788.51 | 0.01 | ± | 0.01 | 0.21 | ± | 0.05** |
| 36:0 | 10.77 | 790.53 | 0.08 | ± | 0.02 | 0.43 | ± | 0.05*** |
| 16:1-22:6 | 8.55 | 804.51 | 0.43 | ± | 0.03 | 0.33 | ± | 0.05 |
| 16:0-22:6 | 9.89 | 806.51 | 77.87 | ± | 0.65 | 59.83 | ± | 2.32*** |
| 18:0-20:4 | 12.52 | 810.56 | 0.41 | ± | 0.04 | 0.70 | ± | 0.09** |
| 39:6 | 10.87 | 820.48 | 0.00 | ± | 0.00 | 0.10 | ± | 0.03* |
| 18:2-22:6 | 8.87 | 830.50 | 0.72 | ± | 0.02 | 1.49 | ± | 0.11*** |
| 18:1-22:6 | 10.18 | 832.51 | 0.98 | ± | 0.03 | 1.19 | ± | 0.14 |
| 18:0-22:6 | 11.97 | 834.55 | 2.65 | ± | 0.13 | 25.36 | ± | 1.71*** |
| 16:0-PC | | | 93.58 | ± | 0.20 | 67.98 | ± | 1.91*** |
| 18:0-PC | | | 3.67 | ± | 0.15 | 27.85 | ± | 1.63*** |

FA, fatty acid; PC, phosphatidylcholine.

Values are represented as mean ± SEM (n = 6). ****P* < 0.001. ***P* < 0.01. **P* < 0.05.

Precursor ion scan mode tandem mass spectrometry (MS/MS) was performed in the positive ionization mode.

**Supplementary Table S2. Phosphatidylethanolamine profiles of the extensor digitorum longus (EDL) and soleus (SOL) muscles**

|  |  |  | % of total PE | | | | | |
| --- | --- | --- | --- | --- | --- | --- | --- | --- |
| FA composition | Retention time (min) | *m/z* | EDL | | | SOL | | |
| 36:3 | 11.34 | 742.36 | 0.14 | ± | 0.10 | 0.41 | ± | 0.27 |
| 36:2 | 13.37 | 744.42 | 1.14 | ± | 0.48 | 1.64 | ± | 0.56 |
| 16:1-22:6 | 8.90 | 762.49 | 0.38 | ± | 0.22 | 0.07 | ± | 0.07 |
| 16:0-22:6 | 10.41 | 764.43 | 25.04 | ± | 0.54 | 12.33 | ± | 1.08*** |
| 38:5 | 11.28 | 766.39 | 0.56 | ± | 0.21 | 0.22 | ± | 0.11 |
| 18:0-20:4 | 13.14 | 768.43 | 1.26 | ± | 0.34 | 1.22 | ± | 0.23 |
| 17:0-22:6 | 11.35 | 778.43 | 0.44 | ± | 0.19 | 0.84 | ± | 0.56 |
| 18:2-22:6 | 9.31 | 788.45 | 4.95 | ± | 0.47 | 3.21 | ± | 0.41* |
| 18:1-22:6 | 10.67 | 790.38 | 13.27 | ± | 0.55 | 10.46 | ± | 1.49 |
| 18:0-22:6 | 12.55 | 792.44 | 51.01 | ± | 1.14 | 68.48 | ± | 2.25*** |
| 40:5 | 14.25 | 794.49 | 0.28 | ± | 0.10 | 1.11 | ± | 0.46 |
| 44:11 | 9.23 | 838.50 | 1.53 | ± | 0.29 | 0.00 | ± | 0.00*** |
| 16:0-PE | | | 25.04 | ± | 0.54 | 12.33 | ± | 1.08*** |
| 18:0-PE | | | 52.27 | ± | 1.01 | 69.70 | ± | 2.17*** |

FA, fatty acid; PE, phosphatidylethanolamine.

Values are represented as mean ± SEM (n = 6). ****P* < 0.001. **P* < 0.05.

Neutral loss scan mode tandem mass spectrometry (MS/MS) was performed in the positive ionization mode.

**Supplementary Table S3. Phosphatidylcholine profiles of the extensor digitorum longus (EDL) and soleus (SOL) muscles overexpressing peroxisome proliferator-activated receptor γ coactivator-1α (*PGC-1α*)**

|  |  |  | % of total PC | | | | | | | | | | | | | | | |
| --- | --- | --- | --- | --- | --- | --- | --- | --- | --- | --- | --- | --- | --- | --- | --- | --- | --- | --- |
| FA composition | Retention time (min) | *m/z* | WT-EDL | | | | *PGC-1α* Tg-EDL | | | | WT-SOL | | | | *PGC-1α* Tg-SOL | | | |
| 14:0-16:0 | 9.96 | 706.61 | 0.32 | ± | 0.07 | ^A^ | 0.13 | ± | 0.02 | ^B^ | 0.08 | ± | 0.03 | ^B^ | 0.05 | ± | 0.02 | ^B^ |
| 16:0-16:1 | 10.42 | 732.62 | 0.65 | ± | 0.09 | ^A^ | 0.33 | ± | 0.01 | ^B^ | 0.18 | ± | 0.01 | ^C^ | 0.13 | ± | 0.02 | ^C^ |
| 16:0-16:0 | 12.05 | 734.65 | 2.37 | ± | 0.61 |  | 1.94 | ± | 0.30 |  | 1.46 | ± | 0.19 |  | 1.53 | ± | 0.25 |  |
| 16:0-18:2 | 10.80 | 758.62 | 9.44 | ± | 0.98 | ^AB^ | 11.35 | ± | 1.34 | ^A^ | 5.30 | ± | 1.07 | ^BC^ | 3.64 | ± | 0.41 | ^C^ |
| 16:0-18:1 | 12.37 | 760.66 | 6.46 | ± | 1.28 | ^A^ | 4.81 | ± | 0.42 | ^AB^ | 5.88 | ± | 0.45 | ^A^ | 3.31 | ± | 0.54 | ^B^ |
| 14:0-22:6 | 8.54 | 778.61 | 0.30 | ± | 0.04 | ^A^ | 0.02 | ± | 0.01 | ^B^ | 0.22 | ± | 0.03 | ^A^ | 0.07 | ± | 0.02 | ^B^ |
| 16:0-20:4 | 10.65 | 782.65 | 12.49 | ± | 1.72 | ^A^ | 5.82 | ± | 0.60 | ^B^ | 3.73 | ± | 0.37 | ^BC^ | 2.77 | ± | 0.41 | ^C^ |
| 36:3 | 11.14 | 784.68 | 0.59 | ± | 0.27 | ^B^ | 1.48 | ± | 0.30 | ^A^ | 0.60 | ± | 0.12 | ^B^ | 0.37 | ± | 0.07 | ^B^ |
| 18:0-18:2 | 13.09 | 786.69 | 1.11 | ± | 0.18 | ^B^ | 9.48 | ± | 1.06 | ^A^ | 3.41 | ± | 0.60 | ^B^ | 3.32 | ± | 0.33 | ^B^ |
| 18:0-18:1 | 14.96 | 788.69 | 0.05 | ± | 0.03 | ^B^ | 0.41 | ± | 0.05 | ^A^ | 0.33 | ± | 0.08 | ^A^ | 0.36 | ± | 0.07 | ^A^ |
| 36:0 | 11.12 | 790.62 | 0.22 | ± | 0.03 | ^C^ | 0.17 | ± | 0.02 | ^C^ | 0.58 | ± | 0.05 | ^A^ | 0.42 | ± | 0.02 | ^B^ |
| 16:1-22:6 | 8.78 | 804.62 | 0.68 | ± | 0.08 | ^A^ | 0.14 | ± | 0.04 | ^C^ | 0.32 | ± | 0.05 | ^B^ | 0.20 | ± | 0.04 | ^BC^ |
| 16:0-22:6 | 10.20 | 806.61 | 55.20 | ± | 3.53 | ^A^ | 32.14 | ± | 3.05 | ^B^ | 38.29 | ± | 2.25 | ^B^ | 38.25 | ± | 1.44 | ^B^ |
| 18:0-20:4 | 12.95 | 810.68 | 0.98 | ± | 0.25 | ^C^ | 7.66 | ± | 0.45 | ^A^ | 2.45 | ± | 0.60 | ^BC^ | 3.53 | ± | 0.63 | ^B^ |
| 39:6 | 11.26 | 820.66 | 0.03 | ± | 0.02 | ^B^ | 0.10 | ± | 0.03 | ^AB^ | 0.11 | ± | 0.04 | ^AB^ | 0.18 | ± | 0.02 | ^A^ |
| 18:2-/22:6 | 9.17 | 830.60 | 1.33 | ± | 0.41 |  | 1.86 | ± | 0.36 |  | 2.02 | ± | 0.31 |  | 1.75 | ± | 0.15 |  |
| 18:1-22:6 | 10.53 | 832.63 | 1.17 | ± | 0.18 |  | 1.12 | ± | 0.07 |  | 1.78 | ± | 0.40 |  | 1.98 | ± | 0.34 |  |
| 18:0-22:6 | 12.38 | 834.64 | 6.61 | ± | 1.38 | ^C^ | 21.06 | ± | 1.86 | ^B^ | 33.27 | ± | 1.75 | ^A^ | 38.14 | ± | 1.78 | ^A^ |
| 16:0-PC | | | 86.61 | ± | 2.42 | ^A^ | 56.39 | ± | 2.95 | ^B^ | 54.83 | ± | 2.09 | ^B^ | 49.63 | ± | 1.36 | ^B^ |
| 18:0-PC | | | 8.75 | ± | 1.72 | ^C^ | 38.61 | ± | 2.45 | ^B^ | 39.45 | ± | 1.81 | ^AB^ | 45.34 | ± | 1.34 | ^A^ |

FA, fatty acid; PC, phosphatidylcholine; WT, wild-type.

Values are represented as mean ± SEM (n = 5–8). Means without a common letter differ significantly (*P* < 0.05).

Precursor ion scan mode tandem mass spectrometry (MS/MS) was performed in the positive ionization mode.

**Supplementary Table S4. Phosphatidylethanolamine profiles of the extensor digitorum longus (EDL) and soleus (SOL) muscles overexpressing peroxisome proliferator-activated receptor γ coactivator-1α (*PGC-1α*)**

|  |  |  | % of total PE | | | | | | | | | | | | | | | |
| --- | --- | --- | --- | --- | --- | --- | --- | --- | --- | --- | --- | --- | --- | --- | --- | --- | --- | --- |
| FA composition | Retention time (min) | *m/z* | WT-EDL | | | | *PGC-1α* Tg-EDL | | | | WT-SOL | | | | *PGC-1α* Tg-SOL | | | |
| 36:3 | 11.72 | 742.56 | 0.04 | ± | 0.03 | ^B^ | 0.83 | ± | 0.14 | ^A^ | 0.15 | ± | 0.06 | ^B^ | 0.12 | ± | 0.04 | ^B^ |
| 36:2 | 13.75 | 744.61 | 0.70 | ± | 0.26 | ^B^ | 2.30 | ± | 0.23 | ^A^ | 1.35 | ± | 0.16 | ^B^ | 1.00 | ± | 0.15 | ^B^ |
| 16:1-22:6 | 9.17 | 762.56 | 0.28 | ± | 0.09 | ^A^ | 0.07 | ± | 0.04 | ^B^ | 0.00 | ± | 0.00 | ^B^ | 0.00 | ± | 0.00 | ^B^ |
| 16:0-22:6 | 10.74 | 764.57 | 22.42 | ± | 3.27 | ^A^ | 9.65 | ± | 0.77 | ^B^ | 10.92 | ± | 1.40 | ^B^ | 8.32 | ± | 1.15 | ^B^ |
| 38:5 | 11.50 | 766.63 | 0.22 | ± | 0.13 |  | 0.16 | ± | 0.08 |  | 0.05 | ± | 0.03 |  | 0.01 | ± | 0.02 |  |
| 18:0-20:4 | 13.60 | 768.59 | 1.48 | ± | 0.24 | ^AB^ | 1.12 | ± | 0.15 | ^B^ | 1.97 | ± | 0.24 | ^A^ | 1.28 | ± | 0.15 | ^B^ |
| 17:0-22:6 | 11.81 | 778.52 | 0.09 | ± | 0.07 | ^AB^ | 0.00 | ± | 0.00 | ^B^ | 0.21 | ± | 0.06 | ^A^ | 0.05 | ± | 0.02 | ^B^ |
| 18:2-22:6 | 9.58 | 788.58 | 4.12 | ± | 0.51 |  | 4.07 | ± | 0.81 |  | 3.01 | ± | 0.35 |  | 2.82 | ± | 0.34 |  |
| 18:1-22:6 | 11.06 | 790.58 | 12.67 | ± | 0.58 | ^AB^ | 18.89 | ± | 1.94 | ^A^ | 7.28 | ± | 1.34 | ^B^ | 15.63 | ± | 1.85 | ^A^ |
| 18:0-22:6 | 13.02 | 792.58 | 55.92 | ± | 3.74 | ^C^ | 61.31 | ± | 3.75 | ^BC^ | 74.74 | ± | 2.25 | ^A^ | 70.59 | ± | 3.41 | ^AB^ |
| 40:5 | 13.75 | 794.65 | 0.29 | ± | 0.15 |  | 0.32 | ± | 0.11 |  | 0.33 | ± | 0.11 |  | 0.18 | ± | 0.07 |  |
| 44:11 | 9.51 | 838.57 | 1.77 | ± | 0.25 | ^A^ | 1.26 | ± | 0.19 | ^A^ | 0.00 | ± | 0.00 | ^B^ | 0.00 | ± | 0.00 | ^B^ |
| 16:0-PE | | | 22.42 | ± | 3.27 | ^A^ | 9.65 | ± | 0.77 | ^B^ | 10.92 | ± | 1.40 | ^B^ | 8.32 | ± | 1.15 | ^B^ |
| 18:0-PE | | | 57.40 | ± | 3.73 | ^C^ | 62.43 | ± | 3.67 | ^BC^ | 76.70 | ± | 2.19 | ^A^ | 71.87 | ± | 3.30 | ^AB^ |

FA, fatty acid; PE, phosphatidylethanolamine; WT, wild-type.

Values are represented as mean ± SEM (n = 5–8). Means without a common letter differ significantly (*P* < 0.05).

Neutral loss scan mode tandem mass spectrometry (MS/MS) was performed in the positive ionization mode.

**Supplementary Table S5. Phosphatidylcholine profiles in the peroxisome proliferator-activated receptor γ coactivator-1α (*PGC-1α*)-deficient (KO) extensor digitorum longus (EDL) and soleus (SOL) muscles**

|  |  |  | % of total PC | | | | | | | | | | | | | | | |
| --- | --- | --- | --- | --- | --- | --- | --- | --- | --- | --- | --- | --- | --- | --- | --- | --- | --- | --- |
| FA composition | Retention time (min) | *m/z* | *PGC-1α*^flox/flox^-EDL | | | | *PGC-1α* KO-EDL | | | | *PGC-1α*^flox/flox^-SOL | | | | *PGC-1α* KO-SOL | | | |
| 14:0-16:0 | 10.03 | 706.62 | 0.46 | ± | 0.03 | ^A^ | 0.47 | ± | 0.03 | ^A^ | 0.08 | ± | 0.01 | ^B^ | 0.13 | ± | 0.01 | ^B^ |
| 16:0-16:1 | 10.51 | 732.62 | 0.74 | ± | 0.06 | ^B^ | 1.15 | ± | 0.04 | ^A^ | 0.10 | ± | 0.03 | ^C^ | 0.15 | ± | 0.02 | ^C^ |
| 16:0-16:0 | 12.11 | 734.64 | 4.32 | ± | 0.53 |  | 3.46 | ± | 0.57 |  | 2.76 | ± | 0.31 |  | 3.65 | ± | 0.34 |  |
| 16:0-18:2 | 10.92 | 758.63 | 8.33 | ± | 0.88 | ^B^ | 12.73 | ± | 2.03 | ^A^ | 6.59 | ± | 0.88 | ^B^ | 9.20 | ± | 0.80 | ^B^ |
| 16:0-18:1 | 12.48 | 760.66 | 8.45 | ± | 0.79 | ^A^ | 10.06 | ± | 2.05 | ^A^ | 2.83 | ± | 0.51 | ^B^ | 5.18 | ± | 0.33 | ^B^ |
| 14:0-22:6 | 8.64 | 778.61 | 0.93 | ± | 0.12 | ^A^ | 0.89 | ± | 0.19 | ^AB^ | 0.47 | ± | 0.12 | ^C^ | 0.54 | ± | 0.06 | ^BC^ |
| 16:0-20:4 | 10.78 | 782.64 | 13.29 | ± | 0.92 | ^A^ | 12.70 | ± | 1.29 | ^A^ | 2.62 | ± | 0.24 | ^B^ | 3.71 | ± | 0.44 | ^B^ |
| 36:3 | 11.30 | 784.67 | 1.15 | ± | 0.42 | ^AB^ | 1.91 | ± | 0.35 | ^A^ | 0.59 | ± | 0.19 | ^B^ | 1.04 | ± | 0.19 | ^B^ |
| 18:0-18:2 | 13.21 | 786.69 | 2.89 | ± | 0.33 | ^B^ | 3.05 | ± | 0.49 | ^B^ | 6.01 | ± | 1.36 | ^A^ | 5.15 | ± | 0.33 | ^A^ |
| 18:0-18:1 | 15.02 | 788.71 | 0.25 | ± | 0.02 |  | 0.27 | ± | 0.04 |  | 0.32 | ± | 0.05 |  | 0.28 | ± | 0.03 |  |
| 36:0 | 11.27 | 790.63 | 0.37 | ± | 0.02 | ^B^ | 0.28 | ± | 0.03 | ^B^ | 0.96 | ± | 0.16 | ^A^ | 0.95 | ± | 0.05 | ^A^ |
| 16:1-22:6 | 8.86 | 804.62 | 2.52 | ± | 0.13 | ^A^ | 2.56 | ± | 0.09 | ^A^ | 0.56 | ± | 0.05 | ^B^ | 0.63 | ± | 0.04 | ^B^ |
| 16:0-22:6 | 10.24 | 806.60 | 40.31 | ± | 3.43 |  | 41.52 | ± | 4.20 |  | 33.32 | ± | 3.83 |  | 38.21 | ± | 1.81 |  |
| 18:0-20:4 | 13.10 | 810.68 | 1.28 | ± | 0.26 |  | 0.96 | ± | 0.16 |  | 1.06 | ± | 0.11 |  | 1.51 | ± | 0.30 |  |
| 39:6 | 11.41 | 820.66 | 0.18 | ± | 0.03 | ^B^ | 0.08 | ± | 0.01 | ^C^ | 0.26 | ± | 0.03 | ^BC^ | 0.14 | ± | 0.03 | ^A^ |
| 18:2-22:6 | 9.27 | 830.61 | 2.85 | ± | 0.18 | ^AB^ | 2.09 | ± | 0.05 | ^C^ | 3.38 | ± | 0.33 | ^A^ | 2.58 | ± | 0.32 | ^BC^ |
| 18:1-22:6 | 10.66 | 832.64 | 1.01 | ± | 0.13 |  | 0.88 | ± | 0.14 |  | 1.08 | ± | 0.06 |  | 1.09 | ± | 0.06 |  |
| 18:0-22:6 | 12.53 | 834.65 | 10.67 | ± | 1.64 | ^C^ | 4.94 | ± | 0.61 | ^C^ | 37.02 | ± | 4.23 | ^A^ | 25.84 | ± | 2.79 | ^B^ |
| 16:0-PC | | | 75.44 | ± | 2.17 | ^A^ | 81.62 | ± | 1.09 | ^A^ | 48.22 | ± | 4.50 | ^C^ | 60.10 | ± | 2.19 | ^B^ |
| 18:0-PC | | | 15.09 | ± | 1.97 | ^C^ | 9.22 | ± | 1.03 | ^D^ | 44.41 | ± | 4.22 | ^A^ | 32.79 | ± | 2.58 | ^B^ |

FA, fatty acid; KO, knockout; PC, phosphatidylcholine.

Values are represented as mean ± SEM (n = 5–8). Means without a common letter differ significantly (*P* < 0.05).

Precursor ion scan mode tandem mass spectrometry (MS/MS) was performed in the positive ionization mode.

**Supplementary Table S6. Phosphatidylethanolamine profiles in the peroxisome proliferator-activated receptor γ coactivator-1α (*PGC-1α*)-deficient (KO) extensor digitorum longus (EDL) and soleus (SOL) muscles**

|  |  |  | % of total PE | | | | | | | | | | | | | | | |
| --- | --- | --- | --- | --- | --- | --- | --- | --- | --- | --- | --- | --- | --- | --- | --- | --- | --- | --- |
| FA composition | Retention time (min) | *m/z* | *PGC-1α*^flox/flox^-EDL | | | | *PGC-1α* KO-EDL | | | | *PGC-1α*^flox/flox^-SOL | | | | *PGC-1α* KO-SOL | | | |
| 36:3 | 11.84 | 742.55 | 0.03 | ± | 0.02 | ^B^ | 0.12 | ± | 0.04 | ^A^ | 0.00 | ± | 0.00 | ^B^ | 0.03 | ± | 0.02 | ^B^ |
| 36:2 | 13.88 | 744.59 | 0.76 | ± | 0.08 |  | 0.80 | ± | 0.18 |  | 0.71 | ± | 0.09 |  | 1.00 | ± | 0.16 |  |
| 16:1-22:6 | 9.29 | 762.55 | 0.14 | ± | 0.03 | ^B^ | 0.24 | ± | 0.05 | ^A^ | 0.00 | ± | 0.00 | ^C^ | 0.00 | ± | 0.00 | ^C^ |
| 16:0-22:6 | 10.88 | 764.56 | 28.96 | ± | 5.30 | ^A^ | 30.27 | ± | 2.98 | ^A^ | 11.46 | ± | 0.84 | ^B^ | 12.61 | ± | 2.27 | ^B^ |
| 38:5 | 11.53 | 766.62 | 0.15 | ± | 0.06 | ^AB^ | 0.31 | ± | 0.11 | ^A^ | 0.02 | ± | 0.02 | ^B^ | 0.03 | ± | 0.02 | ^B^ |
| 18:0-20:4 | 13.72 | 768.61 | 0.60 | ± | 0.04 | ^C^ | 0.68 | ± | 0.09 | ^BC^ | 0.88 | ± | 0.11 | ^AB^ | 1.09 | ± | 0.13 | ^A^ |
| 17:0-22:6 | 11.99 | 778.58 | 0.07 | ± | 0.02 |  | 0.06 | ± | 0.03 |  | 0.10 | ± | 0.03 |  | 0.08 | ± | 0.05 |  |
| 18:2-22:6 | 9.72 | 788.57 | 3.96 | ± | 0.72 | ^A^ | 3.98 | ± | 0.84 | ^A^ | 1.77 | ± | 0.18 | ^B^ | 1.67 | ± | 0.23 | ^B^ |
| 18:1-22:6 | 11.21 | 790.58 | 8.18 | ± | 1.02 | ^B^ | 11.44 | ± | 1.42 | ^A^ | 3.99 | ± | 0.16 | ^C^ | 4.46 | ± | 0.81 | ^C^ |
| 18:0-22:6 | 13.20 | 792.59 | 55.73 | ± | 5.71 | ^B^ | 51.06 | ± | 3.35 | ^B^ | 80.99 | ± | 1.14 | ^A^ | 78.84 | ± | 3.07 | ^A^ |
| 40:5 | 13.87 | 794.65 | 0.30 | ± | 0.20 |  | 0.39 | ± | 0.12 |  | 0.09 | ± | 0.02 |  | 0.20 | ± | 0.10 |  |
| 44:11 | 9.66 | 838.55 | 1.13 | ± | 0.06 | ^A^ | 0.63 | ± | 0.13 | ^B^ | 0.00 | ± | 0.00 | ^C^ | 0.00 | ± | 0.00 | ^C^ |
| 16:0-PE | | | 28.96 | ± | 5.30 | ^A^ | 30.27 | ± | 2.98 | ^A^ | 11.46 | ± | 0.84 | ^B^ | 12.61 | ± | 2.27 | ^B^ |
| 18:0-PE | | | 56.32 | ± | 5.70 | ^B^ | 51.75 | ± | 3.33 | ^B^ | 81.87 | ± | 1.07 | ^A^ | 79.93 | ± | 2.95 | ^A^ |

FA, fatty acid; KO, knockout; PE, phosphatidylethanolamine.

Values are represented as mean ± SEM (n = 5–8). Means without a common letter differ significantly (*P* < 0.05).

Neutral loss scan mode tandem mass spectrometry (MS/MS) was performed in the positive ionization mode.

**Supplemental Table S7. Phosphatidylcholine profiles of C2C12 myotubes knocked down for glycerol-3-phosphate acyltransferase (GPAT)3**

|  | % of total PC | | | | | | | | | | | | | | | |
| --- | --- | --- | --- | --- | --- | --- | --- | --- | --- | --- | --- | --- | --- | --- | --- | --- |
| Species | siGFP | | | | siGPAT3 #1 | | | | siGPAT3 #2 | | | | siGPAT3 #3 | | | |
| PC (16:0-16:0) | 5.46 | ± | 0.15 |  | 5.63 | ± | 0.26 |  | 6.32 | ± | 0.15 | * | 5.99 | ± | 0.19 |  |
| PC (16:0-18:0) | 1.19 | ± | 0.14 |  | 1.25 | ± | 0.10 |  | 0.74 | ± | 0.11 | * | 1.47 | ± | 0.07 |  |
| PC (18:0-18:0) | 0.27 | ± | 0.06 |  | 0.32 | ± | 0.04 |  | 0.26 | ± | 0.01 |  | 0.16 | ± | 0.06 |  |
| PC (16:0-16:1) | 9.21 | ± | 0.37 |  | 9.40 | ± | 0.37 |  | 9.72 | ± | 0.26 |  | 9.28 | ± | 0.41 |  |
| PC (16:0-18:1) | 12.27 | ± | 0.19 |  | 11.72 | ± | 0.31 |  | 12.26 | ± | 0.15 |  | 12.27 | ± | 0.33 |  |
| PC (16:1-16:1) | 7.25 | ± | 0.17 |  | 7.65 | ± | 0.61 |  | 7.16 | ± | 0.22 |  | 6.57 | ± | 0.48 |  |
| PC (16:1-18:0) | 2.11 | ± | 0.29 |  | 2.35 | ± | 0.05 |  | 2.09 | ± | 0.18 |  | 2.17 | ± | 0.16 |  |
| PC (16:1-18:1) | 4.38 | ± | 0.12 |  | 4.95 | ± | 0.39 |  | 4.65 | ± | 0.45 |  | 4.06 | ± | 0.30 |  |
| PC (18:0-18:1) | 5.85 | ± | 0.17 |  | 5.39 | ± | 0.31 |  | 5.79 | ± | 0.08 |  | 5.09 | ± | 0.52 |  |
| PC (18:1-18:1) | 14.78 | ± | 0.22 |  | 16.16 | ± | 0.50 |  | 15.29 | ± | 0.47 |  | 15.02 | ± | 0.09 |  |
| PC (16:0-18:2) | 9.87 | ± | 0.39 |  | 9.44 | ± | 0.49 |  | 9.25 | ± | 0.27 |  | 10.02 | ± | 0.48 |  |
| PC (16:1-18:2) | 2.87 | ± | 0.21 |  | 2.71 | ± | 0.27 |  | 2.71 | ± | 0.17 |  | 2.52 | ± | 0.20 |  |
| PC (18:0-18:2) | 11.24 | ± | 0.29 |  | 10.87 | ± | 0.50 |  | 10.63 | ± | 0.31 |  | 12.09 | ± | 0.51 |  |
| PC (18:1-18:2) | 5.96 | ± | 0.11 |  | 6.03 | ± | 0.42 |  | 5.67 | ± | 0.24 |  | 5.41 | ± | 0.39 |  |
| PC (18:2-18:2) | 0.59 | ± | 0.05 |  | 0.76 | ± | 0.06 |  | 0.61 | ± | 0.05 |  | 0.59 | ± | 0.10 |  |
| PC (16:0-20:4) | 1.66 | ± | 0.16 |  | 1.31 | ± | 0.22 |  | 1.77 | ± | 0.25 |  | 1.88 | ± | 0.17 |  |
| PC (16:1-20:4) | 0.41 | ± | 0.05 |  | 0.38 | ± | 0.01 |  | 0.46 | ± | 0.03 |  | 0.45 | ± | 0.01 |  |
| PC (18:0-20:4) | 1.45 | ± | 0.09 |  | 1.14 | ± | 0.09 |  | 1.42 | ± | 0.15 |  | 1.74 | ± | 0.21 |  |
| PC (18:1-20:4) | 0.97 | ± | 0.10 |  | 0.83 | ± | 0.09 |  | 1.11 | ± | 0.24 |  | 1.13 | ± | 0.13 |  |
| PC (18:2-20:4) | 0.15 | ± | 0.02 |  | 0.10 | ± | 0.01 |  | 0.14 | ± | 0.04 |  | 0.16 | ± | 0.00 |  |
| PC (16:0-20:5) | 0.54 | ± | 0.07 |  | 0.50 | ± | 0.01 |  | 0.57 | ± | 0.07 |  | 0.48 | ± | 0.04 |  |
| PC (16:1-20:5) | 0.12 | ± | 0.01 |  | 0.10 | ± | 0.00 |  | 0.09 | ± | 0.01 |  | 0.11 | ± | 0.03 |  |
| PC (18:0-20:5) | 0.26 | ± | 0.04 |  | 0.22 | ± | 0.03 |  | 0.26 | ± | 0.06 |  | 0.27 | ± | 0.06 |  |
| PC (18:1-20:5) | 0.29 | ± | 0.04 |  | 0.22 | ± | 0.03 |  | 0.34 | ± | 0.03 |  | 0.27 | ± | 0.06 |  |
| PC (18:2-20:5) | 0.02 | ± | 0.01 |  | 0.01 | ± | 0.00 |  | 0.02 | ± | 0.01 |  | 0.01 | ± | 0.00 |  |
| PC (16:0-22:6) | 0.29 | ± | 0.04 |  | 0.17 | ± | 0.01 |  | 0.24 | ± | 0.06 |  | 0.30 | ± | 0.03 |  |
| PC (16:1-22:6) | 0.10 | ± | 0.01 |  | 0.07 | ± | 0.01 |  | 0.09 | ± | 0.01 |  | 0.09 | ± | 0.01 |  |
| PC (18:0-22:6) | 0.18 | ± | 0.03 |  | 0.09 | ± | 0.01 | * | 0.19 | ± | 0.02 |  | 0.16 | ± | 0.02 |  |
| PC (18:1-22:6) | 0.20 | ± | 0.03 |  | 0.16 | ± | 0.01 |  | 0.12 | ± | 0.00 |  | 0.16 | ± | 0.04 |  |
| PC (18:2-22:6) | 0.07 | ± | 0.00 |  | 0.06 | ± | 0.00 |  | 0.07 | ± | 0.02 |  | 0.08 | ± | 0.03 |  |
| 16:0-PC | 40.49 | ± | 0.18 |  | 39.42 | ± | 0.19 |  | 40.86 | ± | 0.12 |  | 41.69 | ± | 0.63 |  |
| 18:0-PC | 19.25 | ± | 0.28 |  | 18.04 | ± | 0.75 |  | 18.54 | ± | 0.54 |  | 19.51 | ± | 0.40 |  |

PC, phosphatidylcholine.

Values are represented as means ± SEM (n = 3). **P* < 0.05 (vs. siGFP).

Tandem mass spectrometry (MS/MS) analyses were performed using multiple reaction monitoring (MRM)in negative ionization mode.

**Supplemental Table S8. Phosphatidylethanolamine profiles of C2C12 myotubes knocked down for glycerol-3-phosphate acyltransferase (GPAT)3**

|  | % of total PE | | | | | | | | | | | | | | | |
| --- | --- | --- | --- | --- | --- | --- | --- | --- | --- | --- | --- | --- | --- | --- | --- | --- |
| Species | siGFP | | | | siGPAT3 #1 | | | | siGPAT3 #2 | | | | siGPAT3 #3 | | | |
| PE (16:0-16:0) | 0.24 | ± | 0.04 |  | 0.31 | ± | 0.02 |  | 0.26 | ± | 0.01 |  | 0.30 | ± | 0.02 |  |
| PE (16:0-18:0) | 0.59 | ± | 0.08 |  | 0.49 | ± | 0.05 |  | 0.59 | ± | 0.01 |  | 0.47 | ± | 0.06 |  |
| PE (18:0-18:0) | 0.38 | ± | 0.03 |  | 0.42 | ± | 0.08 |  | 0.40 | ± | 0.04 |  | 0.31 | ± | 0.03 |  |
| PE (16:0-16:1) | 0.23 | ± | 0.09 |  | 0.32 | ± | 0.05 |  | 0.44 | ± | 0.05 |  | 0.42 | ± | 0.01 |  |
| PE (16:0-18:1) | 2.89 | ± | 0.17 |  | 3.16 | ± | 0.25 |  | 2.76 | ± | 0.29 |  | 2.26 | ± | 0.36 |  |
| PE (16:1-16:1) | 0.80 | ± | 0.09 |  | 0.76 | ± | 0.10 |  | 0.86 | ± | 0.08 |  | 0.55 | ± | 0.04 |  |
| PE (16:1-18:0) | 2.81 | ± | 0.12 |  | 3.41 | ± | 0.03 |  | 2.87 | ± | 0.20 |  | 2.77 | ± | 0.30 |  |
| PE (16:1-18:1) | 0.89 | ± | 0.04 |  | 1.15 | ± | 0.06 | * | 1.02 | ± | 0.05 |  | 1.13 | ± | 0.02 | * |
| PE (18:0-18:1) | 9.95 | ± | 0.52 |  | 10.94 | ± | 0.51 |  | 9.58 | ± | 0.48 |  | 9.07 | ± | 1.11 |  |
| PE (18:1-18:1) | 62.17 | ± | 0.50 |  | 60.64 | ± | 0.73 |  | 64.90 | ± | 2.04 |  | 66.41 | ± | 2.38 |  |
| PE (16:0-18:2) | 0.85 | ± | 0.02 |  | 0.73 | ± | 0.04 |  | 0.68 | ± | 0.08 |  | 0.56 | ± | 0.04 | * |
| PE (16:1-18:2) | 0.29 | ± | 0.02 |  | 0.30 | ± | 0.06 |  | 0.23 | ± | 0.05 |  | 0.18 | ± | 0.03 |  |
| PE (18:0-18:2) | 7.32 | ± | 0.31 |  | 7.77 | ± | 0.29 |  | 5.98 | ± | 0.42 | * | 6.42 | ± | 0.13 |  |
| PE (18:1-18:2) | 1.09 | ± | 0.10 |  | 1.15 | ± | 0.04 |  | 1.03 | ± | 0.17 |  | 0.99 | ± | 0.04 |  |
| PE (18:2-18:2) | 0.04 | ± | 0.01 |  | 0.04 | ± | 0.00 |  | 0.05 | ± | 0.02 |  | 0.04 | ± | 0.00 |  |
| PE (16:0-20:4) | 0.51 | ± | 0.05 |  | 0.47 | ± | 0.04 |  | 0.50 | ± | 0.05 |  | 0.37 | ± | 0.03 |  |
| PE (16:1-20:4) | 0.18 | ± | 0.03 |  | 0.19 | ± | 0.04 |  | 0.18 | ± | 0.06 |  | 0.15 | ± | 0.05 |  |
| PE (18:0-20:4) | 5.40 | ± | 0.29 |  | 4.79 | ± | 0.12 |  | 4.62 | ± | 0.20 |  | 4.71 | ± | 0.24 |  |
| PE (18:1-20:4) | 0.68 | ± | 0.04 |  | 0.63 | ± | 0.07 |  | 0.72 | ± | 0.04 |  | 0.64 | ± | 0.07 |  |
| PE (18:2-20:4) | 0.03 | ± | 0.01 |  | 0.01 | ± | 0.01 |  | 0.03 | ± | 0.01 |  | 0.01 | ± | 0.00 |  |
| PE (16:0-20:5) | 0.08 | ± | 0.03 |  | 0.08 | ± | 0.02 |  | 0.10 | ± | 0.02 |  | 0.04 | ± | 0.00 |  |
| PE (16:1-20:5) | 0.02 | ± | 0.01 |  | 0.03 | ± | 0.00 |  | 0.01 | ± | 0.00 |  | 0.02 | ± | 0.01 |  |
| PE (18:0-20:5) | 0.16 | ± | 0.01 |  | 0.21 | ± | 0.03 |  | 0.11 | ± | 0.01 |  | 0.12 | ± | 0.01 |  |
| PE (18:1-20:5) | 0.06 | ± | 0.02 |  | 0.06 | ± | 0.01 |  | 0.06 | ± | 0.02 |  | 0.05 | ± | 0.02 |  |
| PE (18:2-20:5) | 0.01 | ± | 0.01 |  | 0.00 | ± | 0.00 |  | 0.00 | ± | 0.00 |  | 0.00 | ± | 0.00 |  |
| PE (16:0-22:6) | 0.28 | ± | 0.06 |  | 0.25 | ± | 0.01 |  | 0.28 | ± | 0.03 |  | 0.27 | ± | 0.03 |  |
| PE (16:1-22:6) | 0.35 | ± | 0.04 |  | 0.30 | ± | 0.04 |  | 0.34 | ± | 0.08 |  | 0.22 | ± | 0.03 |  |
| PE (18:0-22:6) | 1.21 | ± | 0.11 |  | 0.95 | ± | 0.09 |  | 0.87 | ± | 0.04 | * | 1.07 | ± | 0.08 |  |
| PE (18:1-22:6) | 0.42 | ± | 0.03 |  | 0.39 | ± | 0.02 |  | 0.45 | ± | 0.00 |  | 0.38 | ± | 0.06 |  |
| PE (18:2-22:6) | 0.08 | ± | 0.01 |  | 0.05 | ± | 0.01 |  | 0.07 | ± | 0.02 |  | 0.07 | ± | 0.02 |  |
| 16:0-PE | 5.67 | ± | 0.24 |  | 5.80 | ± | 0.37 |  | 5.62 | ± | 0.47 |  | 4.69 | ± | 0.46 |  |
| 18:0-PE | 24.41 | ± | 0.64 |  | 25.09 | ± | 0.59 |  | 21.54 | ± | 0.89 |  | 21.70 | ± | 1.41 |  |

PE, phosphatidylethanolamine.

Values are represented as means ± SEM (n = 3). **P* < 0.05 (vs. siGFP).

Tandem mass spectrometry (MS/MS) analyses were performed usingmultiple reaction monitoring (MRM) in negative ionization mode.

**Supplemental Table S9. Phosphatidylcholine profiles of C2C12 myotubes knocked down for lysophosphatidylcholine acyltransferase (LPCAT)1**

|  | % of total PC | | | | | | | | | | | | | | | |
| --- | --- | --- | --- | --- | --- | --- | --- | --- | --- | --- | --- | --- | --- | --- | --- | --- |
| Species | siGFP | | | | siLPCAT1 #1 | | | | siLPCAT1 #2 | | | | siLPCAT1 #3 | | | |
| PC (16:0-16:0) | 5.46 | ± | 0.15 |  | 4.68 | ± | 0.08 | * | 4.84 | ± | 0.19 | * | 4.87 | ± | 0.11 | * |
| PC (16:0-18:0) | 1.19 | ± | 0.14 |  | 1.59 | ± | 0.02 | * | 1.28 | ± | 0.02 |  | 1.69 | ± | 0.03 | ** |
| PC (18:0-18:0) | 0.27 | ± | 0.06 |  | 0.29 | ± | 0.08 |  | 0.54 | ± | 0.10 |  | 0.24 | ± | 0.04 |  |
| PC (16:0-16:1) | 9.21 | ± | 0.37 |  | 7.92 | ± | 0.37 | * | 8.10 | ± | 0.12 |  | 7.73 | ± | 0.33 | * |
| PC (16:0-18:1) | 12.27 | ± | 0.19 |  | 13.78 | ± | 0.64 |  | 12.66 | ± | 0.34 |  | 13.14 | ± | 0.47 |  |
| PC (16:1-16:1) | 7.25 | ± | 0.17 |  | 5.26 | ± | 0.64 | * | 6.50 | ± | 0.27 |  | 4.93 | ± | 0.10 | ** |
| PC (16:1-18:0) | 2.11 | ± | 0.29 |  | 1.88 | ± | 0.14 |  | 2.41 | ± | 0.17 |  | 2.09 | ± | 0.11 |  |
| PC (16:1-18:1) | 4.38 | ± | 0.12 |  | 4.04 | ± | 0.26 |  | 4.86 | ± | 0.34 |  | 4.25 | ± | 0.39 |  |
| PC (18:0-18:1) | 5.85 | ± | 0.17 |  | 5.84 | ± | 0.16 |  | 6.39 | ± | 0.11 |  | 5.73 | ± | 0.36 |  |
| PC (18:1-18:1) | 14.78 | ± | 0.22 |  | 15.69 | ± | 0.49 |  | 16.51 | ± | 0.47 | * | 15.43 | ± | 0.42 |  |
| PC (16:0-18:2) | 9.87 | ± | 0.39 |  | 9.48 | ± | 0.22 |  | 9.17 | ± | 0.41 |  | 10.40 | ± | 0.37 |  |
| PC (16:1-18:2) | 2.87 | ± | 0.21 |  | 2.58 | ± | 0.22 |  | 2.62 | ± | 0.21 |  | 2.51 | ± | 0.21 |  |
| PC (18:0-18:2) | 11.24 | ± | 0.29 |  | 13.14 | ± | 0.80 | * | 11.10 | ± | 0.29 |  | 12.73 | ± | 0.24 |  |
| PC (18:1-18:2) | 5.96 | ± | 0.11 |  | 6.00 | ± | 0.29 |  | 5.79 | ± | 0.46 |  | 6.13 | ± | 0.48 |  |
| PC (18:2-18:2) | 0.59 | ± | 0.05 |  | 0.60 | ± | 0.10 |  | 0.47 | ± | 0.03 |  | 0.69 | ± | 0.07 |  |
| PC (16:0-20:4) | 1.66 | ± | 0.16 |  | 1.75 | ± | 0.20 |  | 1.65 | ± | 0.19 |  | 1.86 | ± | 0.29 |  |
| PC (16:1-20:4) | 0.41 | ± | 0.05 |  | 0.43 | ± | 0.04 |  | 0.40 | ± | 0.05 |  | 0.38 | ± | 0.03 |  |
| PC (18:0-20:4) | 1.45 | ± | 0.09 |  | 1.60 | ± | 0.14 |  | 1.37 | ± | 0.11 |  | 1.66 | ± | 0.08 |  |
| PC (18:1-20:4) | 0.97 | ± | 0.10 |  | 1.13 | ± | 0.15 |  | 1.13 | ± | 0.07 |  | 1.21 | ± | 0.15 |  |
| PC (18:2-20:4) | 0.15 | ± | 0.02 |  | 0.12 | ± | 0.03 |  | 0.16 | ± | 0.00 |  | 0.14 | ± | 0.03 |  |
| PC (16:0-20:5) | 0.54 | ± | 0.07 |  | 0.59 | ± | 0.04 |  | 0.55 | ± | 0.03 |  | 0.59 | ± | 0.06 |  |
| PC (16:1-20:5) | 0.12 | ± | 0.01 |  | 0.10 | ± | 0.02 |  | 0.16 | ± | 0.02 |  | 0.10 | ± | 0.02 |  |
| PC (18:0-20:5) | 0.26 | ± | 0.04 |  | 0.37 | ± | 0.05 |  | 0.26 | ± | 0.04 |  | 0.33 | ± | 0.06 |  |
| PC (18:1-20:5) | 0.29 | ± | 0.04 |  | 0.28 | ± | 0.03 |  | 0.34 | ± | 0.03 |  | 0.35 | ± | 0.05 |  |
| PC (18:2-20:5) | 0.02 | ± | 0.01 |  | 0.02 | ± | 0.01 |  | 0.01 | ± | 0.00 |  | 0.01 | ± | 0.00 |  |
| PC (16:0-22:6) | 0.29 | ± | 0.04 |  | 0.25 | ± | 0.04 |  | 0.22 | ± | 0.05 |  | 0.26 | ± | 0.04 |  |
| PC (16:1-22:6) | 0.10 | ± | 0.01 |  | 0.09 | ± | 0.00 |  | 0.10 | ± | 0.03 |  | 0.07 | ± | 0.01 |  |
| PC (18:0-22:6) | 0.18 | ± | 0.03 |  | 0.20 | ± | 0.01 |  | 0.16 | ± | 0.02 |  | 0.21 | ± | 0.03 |  |
| PC (18:1-22:6) | 0.20 | ± | 0.03 |  | 0.22 | ± | 0.03 |  | 0.17 | ± | 0.00 |  | 0.18 | ± | 0.01 |  |
| PC (18:2-22:6) | 0.07 | ± | 0.00 |  | 0.07 | ± | 0.02 |  | 0.07 | ± | 0.02 |  | 0.07 | ± | 0.01 |  |
| 16:0-PC | 40.49 | ± | 0.18 |  | 40.05 | ± | 0.58 |  | 38.47 | ± | 0.14 | * | 40.55 | ± | 0.26 |  |
| 18:0-PC | 19.25 | ± | 0.28 |  | 21.44 | ± | 0.82 | * | 19.81 | ± | 0.20 |  | 20.90 | ± | 0.14 |  |

PC, phosphatidylcholine.

Values are represented as means ± SEM (n = 3). ***P* < 0.01. **P* < 0.05 (vs. siGFP).

Tandem mass spectrometry (MS/MS) analyses were performed usingmultiple reaction monitoring (MRM) in negative ionization mode.

**Supplemental Table S10. Phosphatidylethanolamine profiles of C2C12 myotubes knocked down for lysophosphatidylcholine acyltransferase (LPCAT)1**

|  | % of total PE | | | | | | | | | | | | | | | |
| --- | --- | --- | --- | --- | --- | --- | --- | --- | --- | --- | --- | --- | --- | --- | --- | --- |
| Species | siGFP | | | | siLPCAT1 #1 | | | | siLPCAT1 #2 | | | | siLPCAT1 #3 | | | |
| PE (16:0-16:0) | 0.24 | ± | 0.04 |  | 0.30 | ± | 0.05 |  | 0.40 | ± | 0.07 |  | 0.31 | ± | 0.03 |  |
| PE (16:0-18:0) | 0.59 | ± | 0.08 |  | 0.62 | ± | 0.05 |  | 0.78 | ± | 0.13 |  | 0.54 | ± | 0.01 |  |
| PE (18:0-18:0) | 0.38 | ± | 0.03 |  | 0.34 | ± | 0.02 |  | 0.33 | ± | 0.04 |  | 0.37 | ± | 0.02 |  |
| PE (16:0-16:1) | 0.23 | ± | 0.09 |  | 0.35 | ± | 0.05 |  | 0.33 | ± | 0.08 |  | 0.21 | ± | 0.04 |  |
| PE (16:0-18:1) | 2.89 | ± | 0.17 |  | 2.82 | ± | 0.41 |  | 3.03 | ± | 0.21 |  | 2.20 | ± | 0.12 |  |
| PE (16:1-16:1) | 0.80 | ± | 0.09 |  | 0.60 | ± | 0.14 |  | 0.76 | ± | 0.04 |  | 0.54 | ± | 0.03 |  |
| PE (16:1-18:0) | 2.81 | ± | 0.12 |  | 2.28 | ± | 0.38 |  | 3.20 | ± | 0.20 |  | 2.75 | ± | 0.22 |  |
| PE (16:1-18:1) | 0.89 | ± | 0.04 |  | 0.50 | ± | 0.23 |  | 1.07 | ± | 0.12 |  | 0.84 | ± | 0.13 |  |
| PE (18:0-18:1) | 9.95 | ± | 0.52 |  | 10.29 | ± | 0.57 |  | 10.34 | ± | 0.63 |  | 8.88 | ± | 0.56 |  |
| PE (18:1-18:1) | 62.17 | ± | 0.50 |  | 63.55 | ± | 2.30 |  | 60.52 | ± | 1.64 |  | 65.32 | ± | 1.67 |  |
| PE (16:0-18:2) | 0.85 | ± | 0.02 |  | 0.78 | ± | 0.06 |  | 0.85 | ± | 0.05 |  | 0.79 | ± | 0.08 |  |
| PE (16:1-18:2) | 0.29 | ± | 0.02 |  | 0.23 | ± | 0.02 |  | 0.28 | ± | 0.02 |  | 0.26 | ± | 0.02 |  |
| PE (18:0-18:2) | 7.32 | ± | 0.31 |  | 7.15 | ± | 0.23 |  | 6.88 | ± | 0.43 |  | 7.10 | ± | 0.35 |  |
| PE (18:1-18:2) | 1.09 | ± | 0.10 |  | 1.02 | ± | 0.08 |  | 1.07 | ± | 0.07 |  | 0.97 | ± | 0.12 |  |
| PE (18:2-18:2) | 0.04 | ± | 0.01 |  | 0.05 | ± | 0.03 |  | 0.02 | ± | 0.01 |  | 0.04 | ± | 0.02 |  |
| PE (16:0-20:4) | 0.51 | ± | 0.05 |  | 0.48 | ± | 0.10 |  | 0.71 | ± | 0.05 |  | 0.59 | ± | 0.15 |  |
| PE (16:1-20:4) | 0.18 | ± | 0.03 |  | 0.23 | ± | 0.03 |  | 0.26 | ± | 0.00 |  | 0.14 | ± | 0.04 |  |
| PE (18:0-20:4) | 5.40 | ± | 0.29 |  | 4.82 | ± | 0.04 |  | 5.34 | ± | 0.04 |  | 4.89 | ± | 0.14 |  |
| PE (18:1-20:4) | 0.68 | ± | 0.04 |  | 0.80 | ± | 0.06 |  | 0.80 | ± | 0.02 |  | 0.75 | ± | 0.04 |  |
| PE (18:2-20:4) | 0.03 | ± | 0.01 |  | 0.03 | ± | 0.01 |  | 0.02 | ± | 0.01 |  | 0.02 | ± | 0.00 |  |
| PE (16:0-20:5) | 0.08 | ± | 0.03 |  | 0.07 | ± | 0.01 |  | 0.11 | ± | 0.04 |  | 0.08 | ± | 0.01 |  |
| PE (16:1-20:5) | 0.02 | ± | 0.01 |  | 0.02 | ± | 0.01 |  | 0.03 | ± | 0.02 |  | 0.01 | ± | 0.00 |  |
| PE (18:0-20:5) | 0.16 | ± | 0.01 |  | 0.21 | ± | 0.01 |  | 0.22 | ± | 0.02 |  | 0.19 | ± | 0.04 |  |
| PE (18:1-20:5) | 0.06 | ± | 0.02 |  | 0.08 | ± | 0.02 |  | 0.09 | ± | 0.01 |  | 0.06 | ± | 0.02 |  |
| PE (18:2-20:5) | 0.01 | ± | 0.01 |  | 0.00 | ± | 0.00 |  | 0.00 | ± | 0.00 |  | 0.00 | ± | 0.00 |  |
| PE (16:0-22:6) | 0.28 | ± | 0.06 |  | 0.39 | ± | 0.04 |  | 0.37 | ± | 0.02 |  | 0.26 | ± | 0.01 |  |
| PE (16:1-22:6) | 0.35 | ± | 0.04 |  | 0.27 | ± | 0.08 |  | 0.39 | ± | 0.04 |  | 0.31 | ± | 0.07 |  |
| PE (18:0-22:6) | 1.21 | ± | 0.11 |  | 1.16 | ± | 0.09 |  | 1.17 | ± | 0.04 |  | 1.09 | ± | 0.06 |  |
| PE (18:1-22:6) | 0.42 | ± | 0.03 |  | 0.51 | ± | 0.02 |  | 0.57 | ± | 0.04 | * | 0.42 | ± | 0.04 |  |
| PE (18:2-22:6) | 0.08 | ± | 0.01 |  | 0.05 | ± | 0.01 |  | 0.04 | ± | 0.01 |  | 0.06 | ± | 0.02 |  |
| 16:0-PE | 5.67 | ± | 0.24 |  | 5.82 | ± | 0.64 |  | 6.59 | ± | 0.52 |  | 4.99 | ± | 0.34 |  |
| 18:0-PE | 24.41 | ± | 0.64 |  | 23.97 | ± | 0.88 |  | 24.29 | ± | 1.07 |  | 22.52 | ± | 0.82 |  |

PE, phosphatidylethanolamine.

Values are represented as means ± SEM (n = 3). **P* < 0.05 (vs. siGFP).

Tandem mass spectrometry (MS/MS) analyses were performed using multiple reaction monitoring (MRM) in negative ionization mode.

**Supplemental Table S11. Phosphatidylcholine profiles of C2C12 myotubes knocked down for lysophosphatidylglycerol acyltransferase (LPGAT)1**

|  | % of total PC | | | | | | | | | | | | | | | |
| --- | --- | --- | --- | --- | --- | --- | --- | --- | --- | --- | --- | --- | --- | --- | --- | --- |
| Species | siGFP | | | | siLPGAT1 #1 | | | | siLPGAT1 #2 | | | | siLPGAT1 #3 | | | |
| PC (16:0-16:0) | 5.78 | ± | 0.25 |  | 7.74 | ± | 0.35 | ** | 8.10 | ± | 0.29 | ** | 6.76 | ± | 0.27 |  |
| PC (16:0-18:0) | 1.00 | ± | 0.16 |  | 0.71 | ± | 0.09 |  | 0.72 | ± | 0.10 |  | 0.99 | ± | 0.04 |  |
| PC (18:0-18:0) | 0.46 | ± | 0.18 |  | 0.10 | ± | 0.04 |  | 0.07 | ± | 0.02 | * | 0.14 | ± | 0.04 |  |
| PC (16:0-16:1) | 9.58 | ± | 0.16 |  | 10.28 | ± | 0.45 |  | 9.72 | ± | 0.35 |  | 9.95 | ± | 0.28 |  |
| PC (16:0-18:1) | 9.91 | ± | 0.59 |  | 10.91 | ± | 0.53 |  | 12.37 | ± | 0.73 | * | 11.10 | ± | 0.43 |  |
| PC (16:1-16:1) | 14.32 | ± | 0.67 |  | 14.39 | ± | 0.48 |  | 12.42 | ± | 0.74 |  | 13.37 | ± | 0.42 |  |
| PC (16:1-18:0) | 2.38 | ± | 0.20 |  | 1.00 | ± | 0.07 | *** | 0.80 | ± | 0.10 | *** | 1.33 | ± | 0.07 | ** |
| PC (16:1-18:1) | 7.45 | ± | 0.25 |  | 7.18 | ± | 0.18 |  | 7.31 | ± | 0.09 |  | 6.29 | ± | 0.07 | ** |
| PC (18:0-18:1) | 5.07 | ± | 0.44 |  | 2.88 | ± | 0.14 | ** | 1.99 | ± | 0.11 | *** | 3.21 | ± | 0.27 | ** |
| PC (18:1-18:1) | 17.55 | ± | 0.25 |  | 18.27 | ± | 0.17 |  | 19.12 | ± | 0.48 | * | 17.97 | ± | 0.21 |  |
| PC (16:0-18:2) | 6.39 | ± | 0.16 |  | 6.82 | ± | 0.39 |  | 7.46 | ± | 0.21 | * | 7.53 | ± | 0.21 | * |
| PC (16:1-18:2) | 3.78 | ± | 0.46 |  | 5.39 | ± | 0.43 |  | 5.59 | ± | 0.74 |  | 5.44 | ± | 0.23 |  |
| PC (18:0-18:2) | 6.81 | ± | 0.19 |  | 3.96 | ± | 0.13 | *** | 3.28 | ± | 0.19 | *** | 4.71 | ± | 0.29 | *** |
| PC (18:1-18:2) | 6.01 | ± | 0.32 |  | 6.57 | ± | 0.21 |  | 6.79 | ± | 0.17 |  | 6.84 | ± | 0.12 |  |
| PC (18:2-18:2) | 0.77 | ± | 0.05 |  | 0.74 | ± | 0.13 |  | 0.98 | ± | 0.15 |  | 1.03 | ± | 0.09 |  |
| PC (16:0-20:4) | 0.54 | ± | 0.10 |  | 0.64 | ± | 0.09 |  | 0.70 | ± | 0.07 |  | 0.76 | ± | 0.02 |  |
| PC (16:1-20:4) | 0.25 | ± | 0.03 |  | 0.48 | ± | 0.04 |  | 0.49 | ± | 0.11 | * | 0.48 | ± | 0.01 | * |
| PC (18:0-20:4) | 0.43 | ± | 0.02 |  | 0.24 | ± | 0.03 | ** | 0.16 | ± | 0.03 | *** | 0.32 | ± | 0.02 | * |
| PC (18:1-20:4) | 0.54 | ± | 0.09 |  | 0.58 | ± | 0.03 |  | 0.60 | ± | 0.11 |  | 0.56 | ± | 0.04 |  |
| PC (18:2-20:4) | 0.05 | ± | 0.00 |  | 0.09 | ± | 0.03 |  | 0.16 | ± | 0.03 | * | 0.12 | ± | 0.00 | * |
| PC (16:0-20:5) | 0.23 | ± | 0.03 |  | 0.31 | ± | 0.03 |  | 0.30 | ± | 0.09 |  | 0.32 | ± | 0.05 |  |
| PC (16:1-20:5) | 0.12 | ± | 0.01 |  | 0.15 | ± | 0.04 |  | 0.16 | ± | 0.05 |  | 0.13 | ± | 0.01 |  |
| PC (18:0-20:5) | 0.12 | ± | 0.03 |  | 0.07 | ± | 0.02 |  | 0.05 | ± | 0.01 |  | 0.06 | ± | 0.01 |  |
| PC (18:1-20:5) | 0.18 | ± | 0.04 |  | 0.27 | ± | 0.06 |  | 0.28 | ± | 0.08 |  | 0.27 | ± | 0.02 |  |
| PC (18:2-20:5) | 0.02 | ± | 0.00 |  | 0.02 | ± | 0.01 |  | 0.01 | ± | 0.01 |  | 0.04 | ± | 0.01 |  |
| PC (16:0-22:6) | 0.09 | ± | 0.01 |  | 0.07 | ± | 0.01 |  | 0.13 | ± | 0.02 |  | 0.11 | ± | 0.01 |  |
| PC (16:1-22:6) | 0.06 | ± | 0.01 |  | 0.05 | ± | 0.01 |  | 0.08 | ± | 0.02 |  | 0.08 | ± | 0.01 |  |
| PC (18:0-22:6) | 0.04 | ± | 0.01 |  | 0.01 | ± | 0.01 |  | 0.01 | ± | 0.00 |  | 0.02 | ± | 0.01 |  |
| PC (18:1-22:6) | 0.06 | ± | 0.01 |  | 0.06 | ± | 0.02 |  | 0.11 | ± | 0.01 |  | 0.08 | ± | 0.01 |  |
| PC (18:2-22:6) | 0.04 | ± | 0.01 |  | 0.03 | ± | 0.00 |  | 0.04 | ± | 0.01 |  | 0.03 | ± | 0.01 |  |
| 16:0-PC | 33.52 | ± | 0.67 |  | 37.48 | ± | 0.91 | * | 39.51 | ± | 1.33 | ** | 37.51 | ± | 0.50 | * |
| 18:0-PC | 12.92 | ± | 0.41 |  | 7.25 | ± | 0.04 | *** | 5.56 | ± | 0.27 | *** | 8.46 | ± | 0.51 | *** |

PC, phosphatidylcholine.

Values are represented as means ± SEM (n = 3). ****P* < 0.001. ***P* < 0.01. **P* < 0.05 (vs. siGFP).

Tandem mass spectrometry (MS/MS) analyses were performed using multiple reaction monitoring (MRM) in negative ionization mode.

**Supplemental Table S12. Phosphatidylethanolamine profiles of C2C12 myotubes knocked down for lysophosphatidylglycerol acyltransferase (LPGAT)1**

|  | % of total PE | | | | | | | | | | | | | | | |
| --- | --- | --- | --- | --- | --- | --- | --- | --- | --- | --- | --- | --- | --- | --- | --- | --- |
| Species | siGFP | | | | siLPGAT1 #1 | | | | siLPGAT1 #2 | | | | siLPGAT1 #3 | | | |
| PE (16:0-16:0) | 0.41 | ± | 0.05 |  | 0.39 | ± | 0.02 |  | 0.46 | ± | 0.04 |  | 0.47 | ± | 0.02 |  |
| PE (16:0-18:0) | 0.43 | ± | 0.11 |  | 0.51 | ± | 0.08 |  | 0.44 | ± | 0.03 |  | 0.56 | ± | 0.04 |  |
| PE (18:0-18:0) | 0.40 | ± | 0.05 |  | 0.26 | ± | 0.03 |  | 0.17 | ± | 0.03 | ** | 0.20 | ± | 0.03 | * |
| PE (16:0-16:1) | 0.72 | ± | 0.10 |  | 0.71 | ± | 0.07 |  | 0.99 | ± | 0.05 |  | 0.64 | ± | 0.04 |  |
| PE (16:0-18:1) | 3.35 | ± | 0.27 |  | 4.72 | ± | 0.08 | ** | 5.02 | ± | 0.19 | *** | 4.41 | ± | 0.12 | * |
| PE (16:1-16:1) | 2.78 | ± | 0.09 |  | 4.44 | ± | 0.35 | * | 3.63 | ± | 0.45 |  | 3.46 | ± | 0.19 |  |
| PE (16:1-18:0) | 5.53 | ± | 0.40 |  | 3.36 | ± | 0.10 | *** | 2.70 | ± | 0.14 | *** | 3.43 | ± | 0.20 | *** |
| PE (16:1-18:1) | 2.33 | ± | 0.14 |  | 2.57 | ± | 0.04 |  | 3.33 | ± | 0.15 | * | 2.31 | ± | 0.12 |  |
| PE (18:0-18:1) | 9.73 | ± | 0.06 |  | 8.07 | ± | 0.18 | ** | 7.22 | ± | 0.30 | *** | 8.34 | ± | 0.19 | ** |
| PE (18:1-18:1) | 53.21 | ± | 0.99 |  | 56.57 | ± | 0.34 | * | 57.24 | ± | 0.80 | * | 56.21 | ± | 0.73 |  |
| PE (16:0-18:2) | 1.09 | ± | 0.05 |  | 1.34 | ± | 0.13 |  | 1.44 | ± | 0.05 |  | 1.21 | ± | 0.12 |  |
| PE (16:1-18:2) | 0.78 | ± | 0.01 |  | 1.45 | ± | 0.07 | ** | 1.48 | ± | 0.08 | *** | 1.37 | ± | 0.11 | ** |
| PE (18:0-18:2) | 9.64 | ± | 0.08 |  | 5.79 | ± | 0.23 | *** | 5.93 | ± | 0.17 | *** | 7.00 | ± | 0.42 | *** |
| PE (18:1-18:2) | 2.12 | ± | 0.13 |  | 2.57 | ± | 0.13 | * | 2.62 | ± | 0.02 | * | 2.37 | ± | 0.10 |  |
| PE (18:2-18:2) | 0.04 | ± | 0.03 |  | 0.09 | ± | 0.04 |  | 0.10 | ± | 0.03 |  | 0.09 | ± | 0.03 |  |
| PE (16:0-20:4) | 0.51 | ± | 0.04 |  | 0.56 | ± | 0.04 |  | 0.56 | ± | 0.05 |  | 0.63 | ± | 0.07 |  |
| PE (16:1-20:4) | 0.28 | ± | 0.04 |  | 0.64 | ± | 0.06 | ** | 0.77 | ± | 0.05 | *** | 0.48 | ± | 0.02 | * |
| PE (18:0-20:4) | 3.69 | ± | 0.19 |  | 2.63 | ± | 0.12 | * | 2.48 | ± | 0.18 | ** | 3.26 | ± | 0.27 |  |
| PE (18:1-20:4) | 0.87 | ± | 0.14 |  | 1.01 | ± | 0.07 |  | 1.22 | ± | 0.13 |  | 1.09 | ± | 0.04 |  |
| PE (18:2-20:4) | 0.02 | ± | 0.02 |  | 0.03 | ± | 0.01 |  | 0.02 | ± | 0.00 |  | 0.05 | ± | 0.02 |  |
| PE (16:0-20:5) | 0.13 | ± | 0.04 |  | 0.11 | ± | 0.02 |  | 0.07 | ± | 0.03 |  | 0.10 | ± | 0.02 |  |
| PE (16:1-20:5) | 0.06 | ± | 0.02 |  | 0.13 | ± | 0.04 |  | 0.12 | ± | 0.01 |  | 0.12 | ± | 0.02 |  |
| PE (18:0-20:5) | 0.11 | ± | 0.02 |  | 0.10 | ± | 0.03 |  | 0.09 | ± | 0.02 |  | 0.20 | ± | 0.04 |  |
| PE (18:1-20:5) | 0.14 | ± | 0.02 |  | 0.16 | ± | 0.01 |  | 0.14 | ± | 0.02 |  | 0.20 | ± | 0.02 |  |
| PE (18:2-20:5) | 0.01 | ± | 0.01 |  | 0.02 | ± | 0.01 |  | 0.01 | ± | 0.00 |  | 0.00 | ± | 0.00 |  |
| PE (16:0-22:6) | 0.23 | ± | 0.02 |  | 0.15 | ± | 0.01 |  | 0.19 | ± | 0.02 |  | 0.17 | ± | 0.02 |  |
| PE (16:1-22:6) | 0.29 | ± | 0.05 |  | 0.68 | ± | 0.10 | * | 0.68 | ± | 0.06 | * | 0.73 | ± | 0.11 | * |
| PE (18:0-22:6) | 0.72 | ± | 0.04 |  | 0.45 | ± | 0.04 | ** | 0.41 | ± | 0.03 | ** | 0.40 | ± | 0.06 | ** |
| PE (18:1-22:6) | 0.34 | ± | 0.02 |  | 0.46 | ± | 0.06 |  | 0.46 | ± | 0.03 |  | 0.46 | ± | 0.05 |  |
| PE (18:2-22:6) | 0.04 | ± | 0.02 |  | 0.04 | ± | 0.01 |  | 0.03 | ± | 0.01 |  | 0.01 | ± | 0.00 |  |
| 16:0-PE | 6.87 | ± | 0.52 |  | 8.49 | ± | 0.23 | * | 9.17 | ± | 0.20 | ** | 8.19 | ± | 0.36 |  |
| 18:0-PE | 24.29 | ± | 0.11 |  | 17.30 | ± | 0.16 | *** | 16.30 | ± | 0.54 | *** | 19.40 | ± | 0.74 | *** |

PE, phosphatidylethanolamine.

Values are represented as means ± SEM (n = 3). ****P* < 0.001. ***P* < 0.01. **P* < 0.05 (vs. siGFP).

Tandem mass spectrometry (MS/MS) analyses were performed using multiple reaction monitoring (MRM) in negative ionization mode.

**Supplemental Table S13. Phosphatidylcholine profiles of C2C12 myotubes knocked down for DDHD domain-containing (DDHD)1**

|  | % of total PC | | | | | | | | | | | |
| --- | --- | --- | --- | --- | --- | --- | --- | --- | --- | --- | --- | --- |
| Species | siGFP | | | | siDDHD1 #1 | | | | siDDHD1 #1 + #3 | | | |
| PC (16:0-16:0) | 5.46 | ± | 0.15 |  | 6.08 | ± | 0.13 |  | 6.36 | ± | 0.48 |  |
| PC (16:0-18:0) | 1.19 | ± | 0.14 |  | 1.33 | ± | 0.11 |  | 1.15 | ± | 0.09 |  |
| PC (18:0-18:0) | 0.27 | ± | 0.06 |  | 0.25 | ± | 0.05 |  | 0.24 | ± | 0.03 |  |
| PC (16:0-16:1) | 9.21 | ± | 0.37 |  | 9.90 | ± | 0.38 |  | 10.37 | ± | 0.40 |  |
| PC (16:0-18:1) | 12.27 | ± | 0.19 |  | 13.13 | ± | 0.40 |  | 12.53 | ± | 0.10 |  |
| PC (16:1-16:1) | 7.25 | ± | 0.17 |  | 6.52 | ± | 0.17 |  | 8.35 | ± | 0.31 | * |
| PC (16:1-18:0) | 2.11 | ± | 0.29 |  | 1.86 | ± | 0.09 |  | 2.05 | ± | 0.13 |  |
| PC (16:1-18:1) | 4.38 | ± | 0.12 |  | 4.59 | ± | 0.17 |  | 5.46 | ± | 0.36 |  |
| PC (18:0-18:1) | 5.85 | ± | 0.17 |  | 4.64 | ± | 0.49 |  | 4.96 | ± | 0.20 |  |
| PC (18:1-18:1) | 14.78 | ± | 0.22 |  | 16.35 | ± | 0.16 | ** | 16.56 | ± | 0.29 | ** |
| PC (16:0-18:2) | 9.87 | ± | 0.39 |  | 9.24 | ± | 0.23 |  | 8.49 | ± | 0.40 |  |
| PC (16:1-18:2) | 2.87 | ± | 0.21 |  | 2.46 | ± | 0.19 |  | 2.98 | ± | 0.25 |  |
| PC (18:0-18:2) | 11.24 | ± | 0.29 |  | 10.21 | ± | 0.25 |  | 8.76 | ± | 0.27 | ** |
| PC (18:1-18:2) | 5.96 | ± | 0.11 |  | 6.23 | ± | 0.27 |  | 5.83 | ± | 0.38 |  |
| PC (18:2-18:2) | 0.59 | ± | 0.05 |  | 0.60 | ± | 0.04 |  | 0.71 | ± | 0.07 |  |
| PC (16:0-20:4) | 1.66 | ± | 0.16 |  | 1.64 | ± | 0.16 |  | 1.27 | ± | 0.15 |  |
| PC (16:1-20:4) | 0.41 | ± | 0.05 |  | 0.45 | ± | 0.03 |  | 0.46 | ± | 0.00 |  |
| PC (18:0-20:4) | 1.45 | ± | 0.09 |  | 1.38 | ± | 0.15 |  | 0.97 | ± | 0.02 | * |
| PC (18:1-20:4) | 0.97 | ± | 0.10 |  | 1.08 | ± | 0.15 |  | 0.91 | ± | 0.10 |  |
| PC (18:2-20:4) | 0.15 | ± | 0.02 |  | 0.15 | ± | 0.01 |  | 0.13 | ± | 0.02 |  |
| PC (16:0-20:5) | 0.54 | ± | 0.07 |  | 0.52 | ± | 0.04 |  | 0.35 | ± | 0.03 |  |
| PC (16:1-20:5) | 0.12 | ± | 0.01 |  | 0.11 | ± | 0.01 |  | 0.14 | ± | 0.03 |  |
| PC (18:0-20:5) | 0.26 | ± | 0.04 |  | 0.23 | ± | 0.02 |  | 0.16 | ± | 0.01 |  |
| PC (18:1-20:5) | 0.29 | ± | 0.04 |  | 0.31 | ± | 0.02 |  | 0.25 | ± | 0.02 |  |
| PC (18:2-20:5) | 0.02 | ± | 0.01 |  | 0.03 | ± | 0.01 |  | 0.02 | ± | 0.00 |  |
| PC (16:0-22:6) | 0.29 | ± | 0.04 |  | 0.22 | ± | 0.02 |  | 0.16 | ± | 0.03 |  |
| PC (16:1-22:6) | 0.10 | ± | 0.01 |  | 0.10 | ± | 0.02 |  | 0.08 | ± | 0.02 |  |
| PC (18:0-22:6) | 0.18 | ± | 0.03 |  | 0.17 | ± | 0.01 |  | 0.09 | ± | 0.02 |  |
| PC (18:1-22:6) | 0.20 | ± | 0.03 |  | 0.15 | ± | 0.01 |  | 0.13 | ± | 0.01 | * |
| PC (18:2-22:6) | 0.07 | ± | 0.00 |  | 0.05 | ± | 0.01 |  | 0.05 | ± | 0.00 | * |
| 16:0-PC | 40.49 | ± | 0.18 |  | 42.06 | ± | 0.62 |  | 40.70 | ± | 0.58 |  |
| 18:0-PC | 19.25 | ± | 0.28 |  | 16.89 | ± | 0.06 | ** | 15.19 | ± | 0.46 | *** |

PC, phosphatidylcholine.

Values are represented as means ± SEM (n = 3). ****P* < 0.001. ***P* < 0.01. **P* < 0.05 (vs. siGFP).

Tandem mass spectrometry (MS/MS) analyses were performed using multiple reaction monitoring (MRM) in negative ionization mode.

**Supplemental Table S14. Phosphatidylethanolamine profiles of C2C12 myotubes knocked down for DDHD domain-containing (DDHD)1**

|  | % of total PE | | | | | | | | | | | |
| --- | --- | --- | --- | --- | --- | --- | --- | --- | --- | --- | --- | --- |
| Species | siGFP | | | | siDDHD1 #1 | | | | siDDHD1 #1 + #3 | | | |
| PE (16:0-16:0) | 0.24 | ± | 0.04 |  | 0.27 | ± | 0.02 |  | 0.31 | ± | 0.02 |  |
| PE (16:0-18:0) | 0.59 | ± | 0.08 |  | 0.43 | ± | 0.08 |  | 0.62 | ± | 0.03 |  |
| PE (18:0-18:0) | 0.38 | ± | 0.03 |  | 0.34 | ± | 0.01 |  | 0.34 | ± | 0.01 |  |
| PE (16:0-16:1) | 0.23 | ± | 0.09 |  | 0.39 | ± | 0.04 |  | 0.55 | ± | 0.14 |  |
| PE (16:0-18:1) | 2.89 | ± | 0.17 |  | 2.83 | ± | 0.25 |  | 2.93 | ± | 0.03 |  |
| PE (16:1-16:1) | 0.80 | ± | 0.09 |  | 0.60 | ± | 0.05 |  | 0.76 | ± | 0.07 |  |
| PE (16:1-18:0) | 2.81 | ± | 0.12 |  | 2.63 | ± | 0.07 |  | 3.16 | ± | 0.09 |  |
| PE (16:1-18:1) | 0.89 | ± | 0.04 |  | 0.98 | ± | 0.01 |  | 1.28 | ± | 0.15 | * |
| PE (18:0-18:1) | 9.95 | ± | 0.52 |  | 8.96 | ± | 0.16 |  | 9.15 | ± | 0.28 |  |
| PE (18:1-18:1) | 62.17 | ± | 0.50 |  | 64.84 | ± | 1.46 |  | 61.96 | ± | 1.00 |  |
| PE (16:0-18:2) | 0.85 | ± | 0.02 |  | 0.65 | ± | 0.05 | * | 0.96 | ± | 0.05 |  |
| PE (16:1-18:2) | 0.29 | ± | 0.02 |  | 0.22 | ± | 0.03 |  | 0.28 | ± | 0.03 |  |
| PE (18:0-18:2) | 7.32 | ± | 0.31 |  | 6.34 | ± | 0.49 |  | 6.98 | ± | 0.29 |  |
| PE (18:1-18:2) | 1.09 | ± | 0.10 |  | 1.01 | ± | 0.01 |  | 1.24 | ± | 0.08 |  |
| PE (18:2-18:2) | 0.04 | ± | 0.01 |  | 0.04 | ± | 0.00 |  | 0.03 | ± | 0.01 |  |
| PE (16:0-20:4) | 0.51 | ± | 0.05 |  | 0.47 | ± | 0.07 |  | 0.61 | ± | 0.04 |  |
| PE (16:1-20:4) | 0.18 | ± | 0.03 |  | 0.11 | ± | 0.01 |  | 0.23 | ± | 0.02 |  |
| PE (18:0-20:4) | 5.40 | ± | 0.29 |  | 5.45 | ± | 0.17 |  | 5.13 | ± | 0.30 |  |
| PE (18:1-20:4) | 0.68 | ± | 0.04 |  | 0.74 | ± | 0.04 |  | 0.89 | ± | 0.02 | * |
| PE (18:2-20:4) | 0.03 | ± | 0.01 |  | 0.04 | ± | 0.01 |  | 0.02 | ± | 0.01 |  |
| PE (16:0-20:5) | 0.08 | ± | 0.03 |  | 0.09 | ± | 0.02 |  | 0.07 | ± | 0.01 |  |
| PE (16:1-20:5) | 0.02 | ± | 0.01 |  | 0.00 | ± | 0.00 |  | 0.04 | ± | 0.02 |  |
| PE (18:0-20:5) | 0.16 | ± | 0.01 |  | 0.20 | ± | 0.03 |  | 0.17 | ± | 0.01 |  |
| PE (18:1-20:5) | 0.06 | ± | 0.02 |  | 0.08 | ± | 0.01 |  | 0.07 | ± | 0.03 |  |
| PE (18:2-20:5) | 0.01 | ± | 0.01 |  | 0.00 | ± | 0.00 |  | 0.01 | ± | 0.00 |  |
| PE (16:0-22:6) | 0.28 | ± | 0.06 |  | 0.26 | ± | 0.02 |  | 0.33 | ± | 0.07 |  |
| PE (16:1-22:6) | 0.35 | ± | 0.04 |  | 0.29 | ± | 0.04 |  | 0.30 | ± | 0.02 |  |
| PE (18:0-22:6) | 1.21 | ± | 0.11 |  | 1.16 | ± | 0.09 |  | 1.05 | ± | 0.03 |  |
| PE (18:1-22:6) | 0.42 | ± | 0.03 |  | 0.50 | ± | 0.02 |  | 0.44 | ± | 0.05 |  |
| PE (18:2-22:6) | 0.08 | ± | 0.01 |  | 0.08 | ± | 0.01 |  | 0.08 | ± | 0.01 |  |
| 16:0-PE | 5.67 | ± | 0.24 |  | 5.40 | ± | 0.43 |  | 6.38 | ± | 0.24 |  |
| 18:0-PE | 24.41 | ± | 0.64 |  | 22.45 | ± | 0.89 |  | 22.82 | ± | 0.54 |  |

PE, phosphatidylethanolamine.

Values are represented as means ± SEM (n = 3). **P* < 0.05 (vs. siGFP).

Tandem mass spectrometry (MS/MS) analyses were performed using multiple reaction monitoring (MRM) in negative ionization mode.

**Supplemental Table S15. Phosphatidylcholine profiles in the lysophosphatidylcholine acyltransferase (*LPCAT*)1-deficient (KO) extensor digitorum longus (EDL) and soleus (SOL) muscles**

|  |  |  | % of total PC | | | | | | | | | | | | | | | |
| --- | --- | --- | --- | --- | --- | --- | --- | --- | --- | --- | --- | --- | --- | --- | --- | --- | --- | --- |
| FA composition | Retention time (min) | m/z | WT-EDL | | | | *LPCAT1* KO-EDL | | | | WT-SOL | | | | *LPCAT*1 KO-SOL | | | |
| 14:0-16:0 | 9.90 | 706.56 | 0.18 | ± | 0.01 | ^A^ | 0.15 | ± | 0.02 | ^A^ | 0.06 | ± | 0.01 | ^B^ | 0.03 | ± | 0.01 | ^B^ |
| 16:0-16:1 | 10.29 | 732.58 | 1.19 | ± | 0.07 | ^A^ | 1.01 | ± | 0.08 | ^A^ | 0.27 | ± | 0.01 | ^B^ | 0.16 | ± | 0.02 | ^B^ |
| 16:0-16:0 | 12.01 | 734.60 | 1.80 | ± | 0.02 |  | 1.63 | ± | 0.06 |  | 2.39 | ± | 0.36 |  | 1.74 | ± | 0.35 |  |
| 16:0-18:2 | 10.71 | 758.60 | 10.59 | ± | 0.57 | ^A^ | 9.12 | ± | 0.34 | ^A^ | 5.49 | ± | 0.27 | ^B^ | 5.38 | ± | 0.21 | ^B^ |
| 16:0-18:1 | 12.33 | 760.60 | 8.95 | ± | 0.21 |  | 8.76 | ± | 0.21 |  | 7.67 | ± | 0.57 |  | 8.02 | ± | 0.46 |  |
| 14:0-22:6 | 8.41 | 778.55 | 0.17 | ± | 0.01 |  | 0.19 | ± | 0.01 |  | 0.14 | ± | 0.02 |  | 0.15 | ± | 0.01 |  |
| 16:0-20:4 | 10.54 | 782.60 | 8.76 | ± | 0.49 | ^A^ | 9.32 | ± | 0.24 | ^A^ | 1.47 | ± | 0.36 | ^B^ | 1.66 | ± | 0.31 | ^B^ |
| 36:3 | 11.03 | 784.63 | 1.25 | ± | 0.06 | ^A^ | 1.18 | ± | 0.06 | ^A^ | 0.77 | ± | 0.03 | ^B^ | 0.78 | ± | 0.02 | ^B^ |
| 18:0-18:2 | 13.02 | 786.65 | 0.93 | ± | 0.04 | ^B^ | 0.83 | ± | 0.06 | ^B^ | 1.75 | ± | 0.05 | ^A^ | 1.80 | ± | 0.03 | ^A^ |
| 18:0-18:1 | 14.96 | 788.67 | 0.08 | ± | 0.03 | ^B^ | 0.08 | ± | 0.02 | ^B^ | 0.54 | ± | 0.07 | ^A^ | 0.63 | ± | 0.06 | ^A^ |
| 36:0 | 11.03 | 790.60 | 0.19 | ± | 0.02 | ^B^ | 0.21 | ± | 0.02 | ^B^ | 0.51 | ± | 0.01 | ^A^ | 0.52 | ± | 0.01 | ^A^ |
| 16:1-22:6 | 8.69 | 804.59 | 0.69 | ± | 0.02 | ^A^ | 0.64 | ± | 0.03 | ^A^ | 0.45 | ± | 0.02 | ^B^ | 0.49 | ± | 0.02 | ^B^ |
| 16:0-22:6 | 10.07 | 806.59 | 57.07 | ± | 1.00 | ^A^ | 58.90 | ± | 0.90 | ^A^ | 45.19 | ± | 1.28 | ^B^ | 47.21 | ± | 0.69 | ^B^ |
| 18:0-20:4 | 12.81 | 810.62 | 0.41 | ± | 0.03 | ^B^ | 0.48 | ± | 0.04 | ^AB^ | 0.74 | ± | 0.09 | ^A^ | 0.74 | ± | 0.08 | ^A^ |
| 39:6 | 11.13 | 820.63 | 0.06 | ± | 0.02 | ^B^ | 0.06 | ± | 0.02 | ^B^ | 0.35 | ± | 0.02 | ^A^ | 0.32 | ± | 0.02 | ^A^ |
| 18:2-22:6 | 9.03 | 830.59 | 1.38 | ± | 0.21 | ^B^ | 1.19 | ± | 0.07 | ^B^ | 3.44 | ± | 0.15 | ^A^ | 3.63 | ± | 0.06 | ^A^ |
| 18:1-22:6 | 10.37 | 832.60 | 1.47 | ± | 0.05 | ^C^ | 1.46 | ± | 0.07 | ^C^ | 3.81 | ± | 0.10 | ^B^ | 4.18 | ± | 0.08 | ^A^ |
| 18:0-22:6 | 12.27 | 834.60 | 4.81 | ± | 0.57 | ^B^ | 4.80 | ± | 0.68 | ^B^ | 24.96 | ± | 0.48 | ^A^ | 22.57 | ± | 0.81 | ^A^ |
| 16:0-PC | | | 88.37 | ± | 0.80 | ^A^ | 88.73 | ± | 0.89 | ^A^ | 62.48 | ± | 0.48 | ^B^ | 64.17 | ± | 0.86 | ^B^ |
| 18:0-PC | | | 6.14 | ± | 0.59 | ^B^ | 6.11 | ± | 0.76 | ^B^ | 27.46 | ± | 0.53 | ^A^ | 25.11 | ± | 0.86 | ^A^ |

FA, fatty acid; KO, knockout; PC, phosphatidylcholine; WT, wild-type.

Values are represented as means ± SEM (n = 5). Means without a common letter differ significantly (*P* < 0.05).

Precursor ion scan mode tandem mass spectrometry (MS/MS) was performed in the positive ionization mode.

**Supplemental Table S16. Phosphatidylethanolamine profiles in the lysophosphatidylcholine acyltransferase (*LPCAT*)1-deficient (KO) extensor digitorum longus (EDL) and soleus (SOL) muscles**

|  |  |  | % of total PE | | | | | | | | | | | | | | | |
| --- | --- | --- | --- | --- | --- | --- | --- | --- | --- | --- | --- | --- | --- | --- | --- | --- | --- | --- |
| FA composition | Retention time (min) | m/z | WT-EDL | | | | *LPCAT1* KO-EDL | | | | WT-SOL | | | | *LPCAT1* KO-SOL | | | |
| 36:2 | 13.27 | 744.54 | 0.81 | ± | 0.11 |  | 0.98 | ± | 0.24 |  | 0.63 | ± | 0.18 |  | 1.07 | ± | 0.33 |  |
| 16:0-22:6 | 10.38 | 764.51 | 22.57 | ± | 0.75 | ^A^ | 16.74 | ± | 2.03 | ^B^ | 10.21 | ± | 1.28 | ^C^ | 8.30 | ± | 0.21 | ^C^ |
| 38:5 | 10.59 | 766.45 | 0.64 | ± | 0.12 |  | 0.77 | ± | 0.27 |  | 0.08 | ± | 0.07 |  | 0.08 | ± | 0.07 |  |
| 18:0-20:4 | 13.08 | 768.58 | 0.57 | ± | 0.32 |  | 0.88 | ± | 0.20 |  | 0.96 | ± | 0.27 |  | 0.73 | ± | 0.42 |  |
| 18:2-22:6 | 9.28 | 788.53 | 4.41 | ± | 0.31 | ^AB^ | 4.64 | ± | 0.22 | ^A^ | 3.24 | ± | 0.20 | ^B^ | 4.08 | ± | 0.29 | ^AB^ |
| 18:1-22:6 | 10.68 | 790.52 | 9.14 | ± | 0.22 |  | 8.75 | ± | 0.51 |  | 8.39 | ± | 0.34 |  | 8.87 | ± | 0.26 |  |
| 18:0-22:6 | 12.56 | 792.55 | 57.16 | ± | 0.73 | ^B^ | 62.61 | ± | 2.15 | ^B^ | 74.05 | ± | 0.82 | ^A^ | 74.23 | ± | 0.44 | ^A^ |
| 40:5 | 12.58 | 794.55 | 2.67 | ± | 0.16 |  | 2.65 | ± | 0.25 |  | 2.44 | ± | 0.23 |  | 2.64 | ± | 0.33 |  |
| 44:11 | 9.17 | 838.51 | 2.02 | ± | 0.17 | ^A^ | 1.98 | ± | 0.23 | ^A^ | 0.00 | ± | 0.00 | ^B^ | 0.00 | ± | 0.00 | ^B^ |
| 16:0-PE | | | 22.57 | ± | 0.75 | ^A^ | 16.74 | ± | 2.03 | ^B^ | 10.21 | ± | 1.28 | ^C^ | 8.30 | ± | 0.21 | ^C^ |
| 18:0-PE | | | 57.73 | ± | 0.88 | ^C^ | 63.49 | ± | 2.05 | ^B^ | 75.02 | ± | 0.78 | ^A^ | 74.96 | ± | 0.35 | ^A^ |

FA, fatty acid; KO, knockout; PC, phosphatidylethanolamine; WT, wild-type.

Values are represented as means ± SEM (n = 5). Means without a common letter differ significantly (*P* < 0.05).

Neutral loss scan mode tandem mass spectrometry (MS/MS) was performed in the positive ionization mode.

**Supplemental Table S17. Phosphatidylcholine profiles in the lysophosphatidylglycerol acyltransferase (*LPGAT*)1-deficient (KO) extensor digitorum longus (EDL) and soleus (SOL) muscles**

|  |  |  | % of total PC | | | | | | | | | | | | | | | |
| --- | --- | --- | --- | --- | --- | --- | --- | --- | --- | --- | --- | --- | --- | --- | --- | --- | --- | --- |
| FA composition | Retention time (min) | m/z | WT-EDL | | | | *LPGAT1* KO-EDL | | | | WT-SOL | | | | *LPGAT1* KO-SOL | | | |
| 14:0-16:0 | 9.87 | 706.57 | 0.13 | ± | 0.01 | ^A^ | 0.15 | ± | 0.01 | ^A^ | 0.06 | ± | 0.00 | ^B^ | 0.08 | ± | 0.01 | ^B^ |
| 16:0-16:1 | 10.26 | 732.61 | 0.84 | ± | 0.02 | ^A^ | 0.68 | ± | 0.02 | ^A^ | 0.27 | ± | 0.02 | ^B^ | 0.24 | ± | 0.06 | ^B^ |
| 16:0-16:0 | 11.95 | 734.62 | 2.51 | ± | 0.25 | ^B^ | 3.40 | ± | 0.34 | ^AB^ | 2.47 | ± | 0.43 | ^B^ | 4.49 | ± | 0.19 | ^A^ |
| 16:0-18:2 | 10.67 | 758.61 | 9.87 | ± | 1.61 |  | 13.64 | ± | 2.58 |  | 6.68 | ± | 0.94 |  | 10.81 | ± | 1.63 |  |
| 16:0-18:1 | 12.24 | 760.63 | 4.53 | ± | 0.33 |  | 5.04 | ± | 0.49 |  | 3.97 | ± | 0.79 |  | 3.15 | ± | 0.28 |  |
| 14:0-22:6 | 8.40 | 778.58 | 0.20 | ± | 0.01 | ^AB^ | 0.16 | ± | 0.02 | ^B^ | 0.16 | ± | 0.00 | ^B^ | 0.25 | ± | 0.01 | ^A^ |
| 16:0-20:4 | 10.47 | 782.60 | 13.79 | ± | 0.64 | ^AB^ | 17.69 | ± | 2.53 | ^A^ | 3.23 | ± | 0.06 | ^C^ | 9.45 | ± | 0.55 | ^B^ |
| 36:3 | 11.11 | 784.63 | 0.13 | ± | 0.12 |  | 0.35 | ± | 0.05 |  | 0.08 | ± | 0.07 |  | 0.28 | ± | 0.18 |  |
| 18:0-18:2 | 12.90 | 786.64 | 1.10 | ± | 0.23 | ^B^ | 0.46 | ± | 0.03 | ^B^ | 2.13 | ± | 0.13 | ^A^ | 0.48 | ± | 0.01 | ^B^ |
| 18:0-18:1 | 14.82 | 788.66 | 0.11 | ± | 0.00 | ^B^ | 0.02 | ± | 0.01 | ^B^ | 0.26 | ± | 0.04 | ^A^ | 0.06 | ± | 0.03 | ^B^ |
| 36:0 | 10.97 | 790.62 | 0.18 | ± | 0.01 | ^B^ | 0.16 | ± | 0.01 | ^B^ | 0.58 | ± | 0.09 | ^AB^ | 0.90 | ± | 0.20 | ^A^ |
| 16:1-22:6 | 8.68 | 804.61 | 0.85 | ± | 0.20 |  | 0.53 | ± | 0.04 |  | 0.37 | ± | 0.03 |  | 0.56 | ± | 0.02 |  |
| 16:0-22:6 | 10.03 | 806.61 | 59.41 | ± | 2.57 |  | 54.60 | ± | 5.59 |  | 45.61 | ± | 1.20 |  | 55.09 | ± | 0.97 |  |
| 18:0-20:4 | 12.74 | 810.66 | 0.50 | ± | 0.04 | ^A^ | 0.02 | ± | 0.01 | ^B^ | 0.76 | ± | 0.11 | ^A^ | 0.03 | ± | 0.01 | ^B^ |
| 39:6 | 11.09 | 820.63 | 0.09 | ± | 0.01 | ^B^ | 0.02 | ± | 0.00 | ^C^ | 0.23 | ± | 0.01 | ^A^ | 0.06 | ± | 0.01 | ^BC^ |
| 18:2-22:6 | 9.01 | 830.61 | 1.69 | ± | 0.08 | ^B^ | 2.49 | ± | 0.30 | ^B^ | 3.23 | ± | 0.34 | ^B^ | 12.53 | ± | 0.90 | ^A^ |
| 18:1-22:6 | 10.34 | 832.62 | 0.65 | ± | 0.05 | ^C^ | 0.56 | ± | 0.02 | ^C^ | 0.98 | ± | 0.05 | ^B^ | 1.28 | ± | 0.07 | ^A^ |
| 18:0-22:6 | 12.19 | 834.64 | 3.41 | ± | 0.31 | ^B^ | 0.02 | ± | 0.01 | ^B^ | 28.94 | ± | 1.23 | ^A^ | 0.27 | ± | 0.05 | ^B^ |
| 16:0-PC | | | 90.95 | ± | 0.19 | ^A^ | 95.06 | ± | 0.36 | ^A^ | 62.23 | ± | 1.33 | ^C^ | 83.23 | ± | 1.03 | ^B^ |
| 18:0-PC | | | 5.11 | ± | 0.29 | ^B^ | 0.52 | ± | 0.04 | ^C^ | 32.08 | ± | 1.00 | ^A^ | 0.84 | ± | 0.02 | ^C^ |

FA, fatty acid; KO, knockout; PC, phosphatidylcholine; WT, wild-type.

Values are represented as means ± SEM (n = 3–4). Means without a common letter differ significantly (*P* < 0.05).

Precursor ion scan mode tandem mass spectrometry (MS/MS) was performed in the positive ionization mode.

**Supplemental Table S18. Phosphatidylethanolamine profiles in the lysophosphatidylglycerol acyltransferase (*LPGAT*)1-deficient (KO) extensor digitorum longus (EDL) and soleus (SOL) muscles**

|  |  |  | % of total PE | | | | | | | | | | | | | | | |
| --- | --- | --- | --- | --- | --- | --- | --- | --- | --- | --- | --- | --- | --- | --- | --- | --- | --- | --- |
| FA composition | Retention time (min) | m/z | WT-EDL | | | | *LPGAT1* KO-EDL | | | | WT-SOL | | | | *LPGAT1* KO-SOL | | | |
| 36.3  36:2 | 11.62  13.61 | 742.45  744.53 | 0.39  1.13 | ±  ± | 0.06  0.19 | ^BC^  ^AB^ | 1.73  0.31 | ±  ± | 0.24  0.03 | ^AB^  ^B^ | 0.29  1.57 | ±  ± | 0.05  0.35 | ^C^  ^A^ | 2.01  1.05 | ±  ± | 0.72  0.18 | ^A^  ^AB^ |
| 16:1-22:6  16:0-22:6 | 9.09  10.65 | 762.53  764.51 | 0.33  25.04 | ± | 0.09  4.46 | ^BC^  ^BC^ | 0.80  36.77 | ± | 0.09  5.40 | ^A^  ^A^ | 0.11  10.04 | ± | 0.04  0.79 | ^C^  ^B^ | 0.59  25.34 | ± | 0.04  4.33 | ^AB^  ^AB^ |
| 38:5 | 11.40 | 767.55 | 0.04 | ± | 0.01 | ^B^ | 0.22 | ± | 0.06 | ^AB^ | 0.01 | ± | 0.01 | ^B^ | 0.43 | ± | 0.19 | ^A^ |
| 18:0-20:4 | 13.46 | 768.55 | 1.21 | ± | 0.12 | ^A^ | 0.27 | ± | 0.04 | ^B^ | 1.29 | ± | 0.22 | ^A^ | 1.51 | ± | 0.29 | ^A^ |
| 17:0-22:6  18:2-22:6 | 11.72  9.51 | 778.52  788.50 | 0.26  5.99 | ± | 0.04  0.93 | ^B^  ^B^ | 0.34  13.39 | ± | 0.06  0.57 | ^AB^  ^A^ | 0.43  3.77 | ± | 0.07  0.57 | ^AB^  ^B^ | 0.60  12.29 | ± | 0.09  1.04 | ^A^  ^A^ |
| 18:1-22:6 | 10.98 | 790.51 | 12.39 | ± | 0.93 | ^B^ | 36.73 | ± | 4.55 | ^A^ | 10.07 | ± | 0.97 | ^B^ | 43.11 | ± | 5.47 | ^A^ |
| 18:0-22:6 | 12.91 | 792.55 | 51.31 | ± | 5.73 | ^B^ | 5.69 | ± | 0.07 | ^C^ | 72.36 | ± | 1.48 | ^A^ | 12.97 | ± | 2.45 | ^C^ |
| 44:11 | 9.41 | 838.58 | 1.92 | ± | 0.29 | ^B^ | 5.69 | ± | 0.55 | ^A^ | 0.07 | ± | 0.02 | ^C^ | 0.10 | ± | 0.07 | ^C^ |
| 16:0-PE | | | 25.04 | ± | 4.46 | ^AB^ | 36.77 | ± | 5.40 | ^A^ | 10.04 | ± | 0.79 | ^B^ | 25.34 | ± | 4.33 | ^AB^ |
| 18:0-PE | | | 52.52 | ± | 5.76 | ^B^ | 4.01 | ± | 0.48 | ^C^ | 73.64 | ± | 1.51 | ^A^ | 14.47 | ± | 2.66 | ^C^ |

FA, fatty acid; KO, knockout; PE, phosphatidylethanolamine; WT, wild-type.

Values are represented as means ± SEM (n = 4–5). Means without a common letter differ significantly (*P* < 0.05).

Neutral loss scan mode tandem mass spectrometry (MS/MS) was performed in the positive ionization mode.

**Supplemental Table S19. Phosphatidylcholine profiles in the DDHD domain-containing (*DDHD*)1-deficient (KO) extensor digitorum longus (EDL) and soleus (SOL) muscles**

|  |  |  | % of total PC | | | | | | | | | | | | | | | |
| --- | --- | --- | --- | --- | --- | --- | --- | --- | --- | --- | --- | --- | --- | --- | --- | --- | --- | --- |
| FA composition | Retention time (min) | m/z | WT-EDL | | | | *DDHD1* KO-EDL | | | | WT-SOL | | | | *DDHD1* KO-SOL | | | |
| 14:0-16:0 | 10.05 | 706.57 | 0.14 | ± | 0.01 | ^A^ | 0.17 | ± | 0.01 | ^A^ | 0.05 | ± | 0.01 | ^B^ | 0.06 | ± | 0.00 | ^B^ |
| 16:0-16:1 | 11.37 | 732.61 | 0.79 | ± | 0.04 | ^A^ | 0.86 | ± | 0.09 | ^A^ | 0.25 | ± | 0.03 | ^B^ | 0.22 | ± | 0.01 | ^B^ |
| 16:0-16:0 | 12.25 | 734.59 | 3.19 | ± | 0.41 |  | 2.60 | ± | 0.34 |  | 2.55 | ± | 0.16 |  | 3.64 | ± | 0.45 |  |
| 16:0-18:2 | 10.88 | 758.56 | 8.28 | ± | 0.90 | ^A^ | 7.23 | ± | 0.77 | ^A^ | 4.11 | ± | 0.27 | ^B^ | 3.57 | ± | 0.19 | ^B^ |
| 16:0-18:1 | 12.55 | 760.59 | 4.83 | ± | 1.14 |  | 5.41 | ± | 0.36 |  | 5.84 | ± | 0.49 |  | 5.18 | ± | 0.20 |  |
| 14:0-22:6 | 8.50 | 778.55 | 0.18 | ± | 0.01 |  | 0.19 | ± | 0.02 |  | 0.14 | ± | 0.01 |  | 0.14 | ± | 0.02 |  |
| 16:0-20:4 | 10.67 | 782.59 | 12.79 | ± | 0.51 | ^A^ | 11.76 | ± | 1.44 | ^A^ | 2.88 | ± | 0.27 | ^B^ | 2.63 | ± | 0.26 | ^B^ |
| 18:0-18:2 | 13.28 | 786.62 | 0.80 | ± | 0.02 | ^B^ | 0.83 | ± | 0.07 | ^B^ | 2.27 | ± | 0.14 | ^A^ | 1.80 | ± | 0.21 | ^A^ |
| 18:0-18:1 | 15.24 | 788.66 | 0.11 | ± | 0.02 | ^B^ | 0.13 | ± | 0.01 | ^B^ | 0.34 | ± | 0.04 | ^A^ | 0.30 | ± | 0.03 | ^A^ |
| 36:0 | 11.22 | 790.58 | 0.15 | ± | 0.01 | ^B^ | 0.13 | ± | 0.01 | ^B^ | 0.59 | ± | 0.04 | ^A^ | 0.49 | ± | 0.07 | ^A^ |
| 16:1-22:6 | 8.80 | 804.56 | 0.54 | ± | 0.02 | ^A^ | 0.53 | ± | 0.05 | ^A^ | 0.30 | ± | 0.01 | ^B^ | 0.38 | ± | 0.05 | ^AB^ |
| 16:0-22:6 | 10.20 | 806.59 | 60.21 | ± | 1.03 | ^A^ | 60.12 | ± | 2.18 | ^A^ | 41.58 | ± | 3.28 | ^B^ | 43.98 | ± | 3.56 | ^B^ |
| 18:0-20:4 | 13.03 | 810.61 | 0.62 | ± | 0.02 | ^AB^ | 0.50 | ± | 0.05 | ^B^ | 1.07 | ± | 0.21 | ^A^ | 0.63 | ± | 0.05 | ^AB^ |
| 39:6 | 11.33 | 820.62 | 0.06 | ± | 0.01 | ^B^ | 0.07 | ± | 0.01 | ^B^ | 0.22 | ± | 0.01 | ^A^ | 0.16 | ± | 0.02 | ^A^ |
| 18:2-22:6 | 9.15 | 830.56 | 1.53 | ± | 0.17 | ^C^ | 1.65 | ± | 0.08 | ^BC^ | 2.42 | ± | 0.20 | ^AB^ | 2.98 | ± | 0.21 | ^A^ |
| 18:1-22:6 | 10.51 | 832.58 | 0.96 | ± | 0.07 | ^B^ | 0.93 | ± | 0.07 | ^B^ | 1.19 | ± | 0.36 | ^AB^ | 2.89 | ± | 0.73 | ^A^ |
| 18:0-22:6 | 12.46 | 834.59 | 4.81 | ± | 0.32 | ^B^ | 6.89 | ± | 1.13 | ^B^ | 34.19 | ± | 2.63 | ^A^ | 30.94 | ± | 2.16 | ^A^ |
| 16:0-PC | | | 90.10 | ± | 0.42 | ^A^ | 87.98 | ± | 1.16 | ^A^ | 57.21 | ± | 2.96 | ^B^ | 59.22 | ± | 2.73 | ^B^ |
| 18:0-PC | | | 6.23 | ± | 0.33 | ^B^ | 8.22 | ± | 1.20 | ^B^ | 37.53 | ± | 2.84 | ^A^ | 33.38 | ± | 2.30 | ^A^ |

FA, fatty acid; KO, knockout; PC, phosphatidylcholine; WT, wild-type.

Values are represented as means ± SEM (n = 5). Means without a common letter differ significantly (*P* < 0.05).

Precursor ion scan mode tandem mass spectrometry (MS/MS) was performed in the positive ionization mode.

**Supplemental Table S20. Phosphatidylethanolamine profiles in the DDHD domain-containing (*DDHD*)1-deficient (KO) extensor digitorum longus (EDL) and soleus (SOL) muscles**

|  |  |  | % of total PE | | | | | | | | | | | | | | | |
| --- | --- | --- | --- | --- | --- | --- | --- | --- | --- | --- | --- | --- | --- | --- | --- | --- | --- | --- |
| FA composition | Retention time (min) | m/z | WT-EDL | | | | *DDHD1* KO-EDL | | | | WT-SOL | | | | *DDHD1* KO-SOL | | | |
| 36:3 | 11.35 | 742.69 | 0.11 | ± | 0.03 | ^B^ | 0.15 | ± | 0.04 | ^AB^ | 0.15 | ± | 0.04 | ^AB^ | 0.31 | ± | 0.04 | ^A^ |
| 36:2 | 13.36 | 744.57 | 1.15 | ± | 0.10 |  | 1.09 | ± | 0.14 |  | 1.20 | ± | 0.26 |  | 1.67 | ± | 0.11 |  |
| 16:1-22:6 | 8.91 | 762.54 | 0.36 | ± | 0.05 | ^A^ | 0.27 | ± | 0.07 | ^AB^ | 0.07 | ± | 0.02 | ^B^ | 0.13 | ± | 0.01 | ^AB^ |
| 16:0-22:6 | 10.41 | 764.53 | 22.03 | ± | 3.21 | ^A^ | 19.72 | ± | 3.04 | ^AB^ | 11.49 | ± | 0.77 | ^B^ | 13.25 | ± | 0.76 | ^AB^ |
| 18:0-20:4 | 13.16 | 768.59 | 1.29 | ± | 0.11 | ^AB^ | 1.04 | ± | 0.14 | ^B^ | 1.88 | ± | 0.19 | ^A^ | 1.56 | ± | 0.18 | ^AB^ |
| 17:0-22:6 | 11.46 | 778.61 | 0.28 | ± | 0.04 | ^AB^ | 0.18 | ± | 0.05 | ^B^ | 0.48 | ± | 0.03 | ^A^ | 0.41 | ± | 0.07 | ^A^ |
| 18:2-22:6 | 9.30 | 788.53 | 6.27 | ± | 0.70 | ^A^ | 6.40 | ± | 0.75 | ^A^ | 3.73 | ± | 0.57 | ^AB^ | 3.40 | ± | 0.33 | ^B^ |
| 18:1-22:6 | 10.72 | 790.61 | 7.90 | ± | 0.75 |  | 4.92 | ± | 1.93 |  | 5.24 | ± | 2.02 |  | 6.80 | ± | 2.11 |  |
| 18:0-22:6 | 12.62 | 792.56 | 60.60 | ± | 3.57 | ^B^ | 66.23 | ± | 1.39 | ^AB^ | 75.76 | ± | 3.27 | ^A^ | 72.47 | ± | 2.71 | ^AB^ |
| 16:0-PE | | | 22.03 | ± | 3.21 | ^A^ | 19.72 | ± | 3.04 | ^AB^ | 11.49 | ± | 0.77 | ^B^ | 13.25 | ± | 0.76 | ^AB^ |
| 18:0-PE | | | 61.89 | ± | 3.52 | ^B^ | 67.27 | ± | 1.43 | ^AB^ | 77.64 | ± | 3.18 | ^A^ | 74.03 | ± | 2.70 | ^AB^ |

FA, fatty acid; KO, knockout; PE, phosphatidylethanolamine; WT, wild-type.

Values are represented as means ± SEM (n = 5). Means without a common letter differ significantly (*P* < 0.05).

Neutral loss scan mode tandem mass spectrometry (MS/MS) was performed in the positive ionization mode.

|  | % of total PC | | | | | | | | | | | | | | | |
| --- | --- | --- | --- | --- | --- | --- | --- | --- | --- | --- | --- | --- | --- | --- | --- | --- |
| Species | *LPGAT1*^flox/flox^-EDL | | | | *LPGAT1* cKO-EDL | | | | *LPGAT1*^flox/flox^-SOL | | | | *LPGAT1* cKO-SOL | | | |
| PC (16:0-16:0) | 18.39 | ± | 1.92 | ^BC^ | 24.45 | ± | 1.43 | ^AB^ | 13.90 | ± | 1.31 | ^C^ | 26.44 | ± | 1.86 | ^A^ |
| PC (16:0-18:0) | 0.16 | ± | 0.06 |  | 0.24 | ± | 0.09 |  | 0.23 | ± | 0.06 |  | 0.16 | ± | 0.06 |  |
| PC (18:0-18:0) | 0.03 | ± | 0.01 | ^B^ | 0.02 | ± | 0.01 | ^B^ | 0.09 | ± | 0.02 | ^A^ | 0.02 | ± | 0.01 | ^B^ |
| PC (16:0-16:1) | 4.64 | ± | 0.37 | ^A^ | 3.73 | ± | 0.17 | ^A^ | 2.60 | ± | 0.26 | ^B^ | 1.42 | ± | 0.23 | ^C^ |
| PC (16:0-18:1) | 12.61 | ± | 0.97 | ^B^ | 16.26 | ± | 0.94 | ^A^ | 6.73 | ± | 0.50 | ^C^ | 9.40 | ± | 0.55 | ^C^ |
| PC (16:1-16:1) | 0.81 | ± | 0.16 | ^A^ | 0.66 | ± | 0.07 | ^A^ | 0.24 | ± | 0.05 | ^B^ | 0.15 | ± | 0.03 | ^B^ |
| PC (16:1-18:0) | 0.06 | ± | 0.03 |  | 0.18 | ± | 0.05 |  | 0.12 | ± | 0.03 |  | 0.08 | ± | 0.02 |  |
| PC (16:1-18:1) | 0.35 | ± | 0.12 |  | 0.24 | ± | 0.08 |  | 0.11 | ± | 0.05 |  | 0.08 | ± | 0.05 |  |
| PC (18:0-18:1) | 0.30 | ± | 0.06 | ^B^ | 0.17 | ± | 0.05 | ^B^ | 0.74 | ± | 0.15 | ^A^ | 0.22 | ± | 0.05 | ^B^ |
| PC (18:1-18:1) | 2.17 | ± | 0.16 | ^B^ | 3.10 | ± | 0.22 | ^A^ | 1.88 | ± | 0.20 | ^B^ | 2.35 | ± | 0.16 | ^B^ |
| PC (16:0-18:2) | 16.11 | ± | 0.73 | ^AB^ | 18.10 | ± | 0.57 | ^A^ | 14.51 | ± | 0.96 | ^B^ | 17.36 | ± | 0.79 | ^AB^ |
| PC (16:1-18:2) | 0.96 | ± | 0.11 |  | 1.32 | ± | 0.17 |  | 1.23 | ± | 0.17 |  | 0.98 | ± | 0.11 |  |
| PC (18:0-18:2) | 4.72 | ± | 0.50 | ^BC^ | 3.77 | ± | 0.36 | ^C^ | 9.11 | ± | 0.84 | ^A^ | 6.13 | ± | 0.45 | ^B^ |
| PC (18:1-18:2) | 2.60 | ± | 0.18 | ^BC^ | 2.46 | ± | 0.10 | ^C^ | 3.90 | ± | 0.27 | ^A^ | 3.30 | ± | 0.15 | ^AB^ |
| PC (18:2-18:2) | 1.66 | ± | 0.14 | ^B^ | 2.47 | ± | 0.21 | ^B^ | 2.77 | ± | 0.31 | ^B^ | 4.83 | ± | 0.67 | ^A^ |
| PC (16:0-20:4) | 7.78 | ± | 0.94 | ^A^ | 6.83 | ± | 0.87 | ^AB^ | 3.53 | ± | 0.30 | ^C^ | 4.67 | ± | 0.56 | ^BC^ |
| PC (16:1-20:4) | 0.33 | ± | 0.08 |  | 0.38 | ± | 0.12 |  | 0.15 | ± | 0.03 |  | 0.24 | ± | 0.02 |  |
| PC (18:0-20:4) | 2.81 | ± | 0.22 | ^A^ | 1.41 | ± | 0.08 | ^B^ | 3.01 | ± | 0.21 | ^A^ | 1.85 | ± | 0.17 | ^B^ |
| PC (18:1-20:4) | 0.87 | ± | 0.08 |  | 0.54 | ± | 0.10 |  | 0.72 | ± | 0.10 |  | 0.59 | ± | 0.07 |  |
| PC (18:2-20:4) | 0.83 | ± | 0.07 | ^B^ | 0.97 | ± | 0.11 | ^B^ | 0.64 | ± | 0.05 | ^B^ | 1.72 | ± | 0.13 | ^A^ |
| PC (16:0-20:5) | 0.61 | ± | 0.09 | ^C^ | 0.57 | ± | 0.01 | ^C^ | 1.15 | ± | 0.04 | ^B^ | 1.53 | ± | 0.10 | ^A^ |
| PC (16:1-20:5) | 0.00 | ± | 0.00 |  | 0.01 | ± | 0.01 |  | 0.00 | ± | 0.00 |  | 0.01 | ± | 0.00 |  |
| PC (18:0-20:5) | 0.08 | ± | 0.02 | ^B^ | 0.02 | ± | 0.01 | ^B^ | 0.67 | ± | 0.09 | ^A^ | 0.14 | ± | 0.05 | ^B^ |
| PC (18:1-20:5) | 0.03 | ± | 0.01 | ^BC^ | 0.01 | ± | 0.00 | ^C^ | 0.13 | ± | 0.04 | ^AB^ | 0.15 | ± | 0.03 | ^A^ |
| PC (18:2-20:5) | 0.00 | ± | 0.00 | ^B^ | 0.01 | ± | 0.01 | ^B^ | 0.05 | ± | 0.01 | ^B^ | 0.25 | ± | 0.06 | ^A^ |
| PC (16:0-22:6) | 13.44 | ± | 2.58 |  | 8.81 | ± | 0.91 |  | 13.46 | ± | 1.47 |  | 9.29 | ± | 0.70 |  |
| PC (16:1-22:6) | 1.15 | ± | 0.11 |  | 0.96 | ± | 0.08 |  | 1.22 | ± | 0.17 |  | 0.79 | ± | 0.07 |  |
| PC (18:0-22:6) | 4.23 | ± | 0.26 | ^B^ | 0.66 | ± | 0.13 | ^C^ | 12.59 | ± | 0.87 | ^A^ | 1.23 | ± | 0.14 | ^C^ |
| PC (18:1-22:6) | 0.80 | ± | 0.23 | ^AB^ | 0.33 | ± | 0.08 | ^B^ | 1.29 | ± | 0.18 | ^A^ | 0.79 | ± | 0.13 | ^AB^ |
| PC (18:2-22:6) | 1.46 | ± | 0.08 | ^B^ | 1.32 | ± | 0.07 | ^B^ | 3.24 | ± | 0.26 | ^A^ | 3.81 | ± | 0.21 | ^A^ |
| 16:0-PC | 73.75 | ± | 0.96 | ^B^ | 78.99 | ± | 1.03 | ^A^ | 56.11 | ± | 0.54 | ^D^ | 70.28 | ± | 0.88 | ^C^ |
| 18:0-PC | 12.16 | ± | 0.75 | ^B^ | 6.04 | ± | 0.35 | ^D^ | 26.20 | ± | 0.67 | ^A^ | 9.59 | ± | 0.45 | ^C^ |

**Supplemental Table S21. Phosphatidylcholine profiles in the extensor digitorum longus (EDL) and soleus (SOL) from conditional lysophosphatidylglycerol acyltransferase 1-deficient (*LPGAT1* cKO) mice**

KO, knockout; PC, phosphatidylcholine.

Values are represented as means ± SEM (n = 6). Means without a common letter differ significantly (*P* < 0.05).

Tandem mass spectrometry (MS/MS) analyses were performed using multiple reaction monitoring (MRM) in negative ionization mode.

**Supplemental Table S22. Phosphatidylethanolamine profiles in the extensor digitorum longus (EDL) and soleus (SOL) from conditional lysophosphatidylglycerol acyltransferase 1-deficient (*LPGAT1* cKO) mice**

|  | % of total PE | | | | | | | | | | | | | | | |
| --- | --- | --- | --- | --- | --- | --- | --- | --- | --- | --- | --- | --- | --- | --- | --- | --- |
| Species | *LPGAT1*^flox/flox^-EDL | | | | *LPGAT1* cKO-EDL | | | | *LPGAT1*^flox/flox^-SOL | | | | *LPGAT1* cKO-SOL | | | |
| PE (16:0-16:0) | 0.38 | ± | 0.12 | ^B^ | 0.79 | ± | 0.11 | ^A^ | 0.15 | ± | 0.03 | ^B^ | 0.90 | ± | 0.04 | ^A^ |
| PE (16:0-18:0) | 0.22 | ± | 0.06 | ^B^ | 0.83 | ± | 0.17 | ^A^ | 0.33 | ± | 0.10 | ^B^ | 0.40 | ± | 0.07 | ^AB^ |
| PE (18:0-18:0) | 0.03 | ± | 0.01 |  | 0.06 | ± | 0.03 |  | 0.08 | ± | 0.02 |  | 0.04 | ± | 0.02 |  |
| PE (16:0-16:1) | 0.11 | ± | 0.05 |  | 0.10 | ± | 0.04 |  | 0.00 | ± | 0.00 |  | 0.06 | ± | 0.02 |  |
| PE (16:0-18:1) | 1.18 | ± | 0.23 | ^BC^ | 2.70 | ± | 0.31 | ^A^ | 0.77 | ± | 0.09 | ^C^ | 1.84 | ± | 0.23 | ^AB^ |
| PE (16:1-16:1) | 0.10 | ± | 0.03 |  | 0.16 | ± | 0.06 |  | 0.05 | ± | 0.02 |  | 0.06 | ± | 0.02 |  |
| PE (16:1-18:0) | 0.39 | ± | 0.04 | ^A^ | 0.13 | ± | 0.08 | ^B^ | 0.12 | ± | 0.04 | ^B^ | 0.10 | ± | 0.05 | ^B^ |
| PE (16:1-18:1) | 0.22 | ± | 0.12 |  | 0.14 | ± | 0.07 |  | 0.15 | ± | 0.04 |  | 0.09 | ± | 0.03 |  |
| PE (18:0-18:1) | 0.79 | ± | 0.17 |  | 0.76 | ± | 0.16 |  | 1.04 | ± | 0.20 |  | 0.82 | ± | 0.10 |  |
| PE (18:1-18:1) | 3.65 | ± | 0.33 | ^AB^ | 5.02 | ± | 0.29 | ^A^ | 3.05 | ± | 0.49 | ^B^ | 3.09 | ± | 0.26 | ^B^ |
| PE (16:0-18:2) | 3.26 | ± | 0.18 | ^BC^ | 5.87 | ± | 0.32 | ^A^ | 2.94 | ± | 0.26 | ^C^ | 4.05 | ± | 0.20 | ^B^ |
| PE (16:1-18:2) | 0.21 | ± | 0.04 | ^B^ | 0.69 | ± | 0.10 | ^A^ | 0.38 | ± | 0.11 | ^AB^ | 0.57 | ± | 0.10 | ^AB^ |
| PE (18:0-18:2) | 9.14 | ± | 0.71 | ^B^ | 4.02 | ± | 0.18 | ^C^ | 12.14 | ± | 0.93 | ^AB^ | 7.21 | ± | 0.30 | ^B^ |
| PE (18:1-18:2) | 2.12 | ± | 0.19 | ^B^ | 5.29 | ± | 0.21 | ^A^ | 2.65 | ± | 0.29 | ^B^ | 4.74 | ± | 0.39 | ^A^ |
| PE (18:2-18:2) | 0.63 | ± | 0.04 | ^B^ | 1.40 | ± | 0.17 | ^A^ | 0.76 | ± | 0.14 | ^B^ | 1.56 | ± | 0.21 | ^A^ |
| PE (16:0-20:4) | 2.14 | ± | 0.18 | ^B^ | 5.13 | ± | 0.30 | ^A^ | 1.50 | ± | 0.11 | ^B^ | 4.79 | ± | 0.31 | ^A^ |
| PE (16:1-20:4) | 0.18 | ± | 0.05 | ^C^ | 0.80 | ± | 0.05 | ^A^ | 0.15 | ± | 0.02 | ^C^ | 0.60 | ± | 0.05 | ^B^ |
| PE (18:0-20:4) | 12.17 | ± | 0.70 | ^BC^ | 9.49 | ± | 0.56 | ^C^ | 15.06 | ± | 0.81 | ^B^ | 19.42 | ± | 1.17 | ^A^ |
| PE (18:1-20:4) | 1.93 | ± | 0.11 | ^B^ | 5.25 | ± | 0.21 | ^A^ | 1.68 | ± | 0.15 | ^B^ | 5.77 | ± | 0.30 | ^A^ |
| PE (18:2-20:4) | 0.35 | ± | 0.06 | ^C^ | 1.20 | ± | 0.10 | ^B^ | 0.19 | ± | 0.04 | ^C^ | 1.58 | ± | 0.06 | ^A^ |
| PE (16:0-20:5) | 0.10 | ± | 0.04 | ^B^ | 0.28 | ± | 0.03 | ^B^ | 0.15 | ± | 0.03 | ^B^ | 0.71 | ± | 0.08 | ^A^ |
| PE (16:1-20:5) | 0.00 | ± | 0.00 |  | 0.00 | ± | 0.00 |  | 0.00 | ± | 0.00 |  | 0.04 | ± | 0.03 |  |
| PE (18:0-20:5) | 0.35 | ± | 0.06 | ^C^ | 0.17 | ± | 0.04 | ^C^ | 0.69 | ± | 0.05 | ^B^ | 1.32 | ± | 0.09 | ^A^ |
| PE (18:1-20:5) | 0.03 | ± | 0.02 | ^B^ | 0.10 | ± | 0.03 | ^B^ | 0.06 | ± | 0.01 | ^B^ | 0.33 | ± | 0.03 | ^A^ |
| PE (18:2-20:5) | 0.01 | ± | 0.01 | ^B^ | 0.12 | ± | 0.03 | ^B^ | 0.12 | ± | 0.02 | ^B^ | 0.47 | ± | 0.10 | ^A^ |
| PE (16:0-22:6) | 11.70 | ± | 1.09 | ^A^ | 10.84 | ± | 0.72 | ^A^ | 7.51 | ± | 0.62 | ^B^ | 5.41 | ± | 0.37 | ^B^ |
| PE (16:1-22:6) | 2.42 | ± | 0.41 | ^B^ | 5.87 | ± | 0.52 | ^A^ | 1.49 | ± | 0.24 | ^B^ | 2.56 | ± | 0.35 | ^B^ |
| PE (18:0-22:6) | 30.95 | ± | 1.76 | ^A^ | 8.60 | ± | 0.36 | ^B^ | 30.29 | ± | 1.51 | ^A^ | 12.63 | ± | 0.29 | ^B^ |
| PE (18:1-22:6) | 7.00 | ± | 0.40 | ^B^ | 9.36 | ± | 0.68 | ^A^ | 7.53 | ± | 0.29 | ^B^ | 7.47 | ± | 0.25 | ^B^ |
| PE (18:2-22:6) | 8.25 | ± | 0.89 | ^B^ | 14.86 | ± | 1.28 | ^A^ | 8.97 | ± | 0.85 | ^B^ | 11.38 | ± | 1.18 | ^AB^ |
| 16:0-PE | 19.08 | ± | 1.16 | ^B^ | 26.53 | ± | 1.17 | ^A^ | 13.36 | ± | 0.41 | ^C^ | 18.16 | ± | 0.37 | ^B^ |
| 18:0-PE | 53.43 | ± | 1.26 | ^B^ | 23.09 | ± | 1.01 | ^D^ | 59.30 | ± | 1.45 | ^A^ | 41.44 | ± | 1.40 | ^C^ |

KO, knockout; PE, phosphatidylethanolamine.

Values are represented as means ± SEM (n = 6). Means without a common letter differ significantly (*P* < 0.05).

Tandem mass spectrometry (MS/MS) analyses were performed using multiple reaction monitoring (MRM) in negative ionization mode.

**Supplemental Table S23. Phosphatidylserine profiles in the extensor digitorum longus (EDL) and soleus (SOL) from conditional lysophosphatidylglycerol acyltransferase 1-deficient (*LPGAT1* cKO) mice**

|  | % of total PS | | | | | | | | | | | | | | | |
| --- | --- | --- | --- | --- | --- | --- | --- | --- | --- | --- | --- | --- | --- | --- | --- | --- |
| Species | *LPGAT1*^flox/flox^-EDL | | | | *LPGAT1* cKO-EDL | | | | *LPGAT1*^flox/flox^-SOL | | | | *LPGAT1* cKO-SOL | | | |
| PS (16:0-16:0) | 0.00 | ± | 0.00 |  | 0.01 | ± | 0.01 |  | 0.00 | ± | 0.00 |  | 0.01 | ± | 0.01 |  |
| PS (16:0-18:0) | 0.00 | ± | 0.00 |  | 0.09 | ± | 0.07 |  | 0.04 | ± | 0.03 |  | 0.05 | ± | 0.04 |  |
| PS (18:0-18:0) | 0.84 | ± | 0.33 |  | 0.98 | ± | 0.18 |  | 0.74 | ± | 0.23 |  | 1.22 | ± | 0.30 |  |
| PS (16:0-16:1) | 0.03 | ± | 0.03 |  | 0.01 | ± | 0.00 |  | 0.02 | ± | 0.02 |  | 0.00 | ± | 0.00 |  |
| PS (16:0-18:1) | 0.13 | ± | 0.06 |  | 0.18 | ± | 0.06 |  | 0.23 | ± | 0.08 |  | 0.21 | ± | 0.06 |  |
| PS (16:1-16:1) | 0.00 | ± | 0.00 |  | 0.00 | ± | 0.00 |  | 0.00 | ± | 0.00 |  | 0.01 | ± | 0.01 |  |
| PS (16:1-18:0) | 0.14 | ± | 0.05 |  | 0.20 | ± | 0.05 |  | 0.16 | ± | 0.06 |  | 0.18 | ± | 0.05 |  |
| PS (16:1-18:1) | 0.07 | ± | 0.03 | ^C^ | 0.40 | ± | 0.11 | ^B^ | 0.13 | ± | 0.04 | ^C^ | 0.78 | ± | 0.06 | ^A^ |
| PS (18:0-18:1) | 14.52 | ± | 1.43 |  | 10.80 | ± | 1.79 |  | 10.82 | ± | 0.81 |  | 13.91 | ± | 1.58 |  |
| PS (18:1-18:1) | 3.13 | ± | 0.65 | ^B^ | 1.62 | ± | 0.52 | ^B^ | 6.67 | ± | 0.62 | ^A^ | 5.48 | ± | 0.56 | ^A^ |
| PS (16:0-18:2) | 0.02 | ± | 0.01 |  | 0.57 | ± | 0.09 |  | 0.17 | ± | 0.06 |  | 0.61 | ± | 0.13 |  |
| PS (16:1-18:2) | 0.00 | ± | 0.00 |  | 0.00 | ± | 0.00 |  | 0.00 | ± | 0.00 |  | 0.01 | ± | 0.01 |  |
| PS (18:0-18:2) | 3.81 | ± | 0.48 | ^B^ | 1.62 | ± | 0.21 | ^C^ | 6.32 | ± | 0.61 | ^A^ | 3.61 | ± | 0.75 | ^BC^ |
| PS (18:1-18:2) | 0.17 | ± | 0.10 |  | 0.32 | ± | 0.09 |  | 0.52 | ± | 0.08 |  | 0.40 | ± | 0.10 |  |
| PS (18:2-18:2) | 0.00 | ± | 0.00 | ^B^ | 0.12 | ± | 0.03 | ^AB^ | 0.03 | ± | 0.01 | ^B^ | 0.26 | ± | 0.08 | ^A^ |
| PS (16:0-20:4) | 0.03 | ± | 0.02 | ^C^ | 0.16 | ± | 0.05 | ^AB^ | 0.07 | ± | 0.02 | ^BC^ | 0.19 | ± | 0.03 | ^A^ |
| PS (16:1-20:4) | 0.00 | ± | 0.00 | ^B^ | 0.02 | ± | 0.01 | ^AB^ | 0.00 | ± | 0.00 | ^B^ | 0.04 | ± | 0.02 | ^A^ |
| PS (18:0-20:4) | 0.74 | ± | 0.19 |  | 0.31 | ± | 0.14 |  | 1.29 | ± | 0.42 |  | 0.57 | ± | 0.23 |  |
| PS (18:1-20:4) | 0.25 | ± | 0.07 |  | 0.59 | ± | 0.43 |  | 0.36 | ± | 0.14 |  | 0.37 | ± | 0.15 |  |
| PS (18:2-20:4) | 6.83 | ± | 1.78 |  | 9.95 | ± | 4.50 |  | 3.56 | ± | 0.91 |  | 7.08 | ± | 3.23 |  |
| PS (16:0-20:5) | 0.00 | ± | 0.00 |  | 0.04 | ± | 0.01 |  | 0.00 | ± | 0.00 |  | 0.07 | ± | 0.04 |  |
| PS (16:1-20:5) | 0.00 | ± | 0.00 |  | 0.03 | ± | 0.02 |  | 0.00 | ± | 0.00 |  | 0.00 | ± | 0.00 |  |
| PS (18:0-20:5) | 0.66 | ± | 0.20 | ^C^ | 3.38 | ± | 0.43 | ^A^ | 0.35 | ± | 0.10 | ^C^ | 2.22 | ± | 0.31 | ^B^ |
| PS (18:1-20:5) | 7.37 | ± | 0.96 | ^C^ | 21.81 | ± | 1.76 | ^A^ | 3.96 | ± | 0.67 | ^C^ | 13.92 | ± | 1.41 | ^B^ |
| PS (18:2-20:5) | 0.05 | ± | 0.01 | ^B^ | 0.38 | ± | 0.08 | ^A^ | 0.11 | ± | 0.04 | ^B^ | 0.19 | ± | 0.05 | ^AB^ |
| PS (16:0-22:6) | 7.76 | ± | 1.07 | ^B^ | 22.46 | ± | 2.34 | ^A^ | 4.23 | ± | 0.57 | ^C^ | 13.21 | ± | 0.96 | ^B^ |
| PS (16:1-22:6) | 0.07 | ± | 0.03 | ^B^ | 0.25 | ± | 0.04 | ^A^ | 0.04 | ± | 0.02 | ^B^ | 0.16 | ± | 0.05 | ^AB^ |
| PS (18:0-22:6) | 43.98 | ± | 3.96 | ^A^ | 7.42 | ± | 0.81 | ^C^ | 53.17 | ± | 2.08 | ^A^ | 25.50 | ± | 1.36 | ^B^ |
| PS (18:1-22:6) | 6.48 | ± | 0.48 | ^B^ | 11.47 | ± | 0.79 | ^A^ | 5.58 | ± | 1.22 | ^B^ | 5.80 | ± | 1.94 | ^B^ |
| PS (18:2-22:6) | 2.74 | ± | 0.33 | ^BC^ | 4.77 | ± | 0.48 | ^A^ | 1.30 | ± | 0.34 | ^C^ | 3.89 | ± | 0.58 | ^AB^ |
| 16:0-PS | 7.96 | ± | 1.06 | ^C^ | 23.52 | ± | 2.25 | ^A^ | 4.76 | ± | 0.52 | ^C^ | 14.35 | ± | 0.94 | ^B^ |
| 18:0-PS | 64.56 | ± | 4.68 | ^A^ | 24.51 | ± | 2.54 | ^C^ | 72.69 | ± | 2.54 | ^A^ | 47.03 | ± | 3.34 | ^B^ |

KO, knockout; PS, phosphatidylserine.

Values are represented as means ± SEM (n = 6). Means without a common letter differ significantly (*P* < 0.05).

Tandem mass spectrometry (MS/MS) analyses were performed using multiple reaction monitoring (MRM) in negative ionization mode.

**Supplemental Table S24. Phosphatidylinositol profiles in the extensor digitorum longus (EDL) and soleus (SOL) from conditional lysophosphatidylglycerol acyltransferase 1-deficient (*LPGAT1* cKO) mice**

|  | % of total PI | | | | | | | | | | | | | | | |
| --- | --- | --- | --- | --- | --- | --- | --- | --- | --- | --- | --- | --- | --- | --- | --- | --- |
| Species | *LPGAT1*^flox/flox^-EDL | | | | *LPGAT1* cKO-EDL | | | | *LPGAT1*^flox/flox^-SOL | | | | *LPGAT1* cKO-SOL | | | |
| PI (16:0-16:0) | 0.29 | ± | 0.05 |  | 0.39 | ± | 0.06 |  | 0.58 | ± | 0.15 |  | 0.53 | ± | 0.16 |  |
| PI (16:0-18:0) | 2.61 | ± | 0.36 | ^B^ | 3.02 | ± | 0.13 | ^B^ | 2.93 | ± | 0.35 | ^B^ | 5.70 | ± | 0.47 | ^A^ |
| PI (18:0-18:0) | 0.01 | ± | 0.01 |  | 0.05 | ± | 0.03 |  | 0.00 | ± | 0.00 |  | 0.14 | ± | 0.10 |  |
| PI (16:0-16:1) | 0.15 | ± | 0.05 |  | 0.30 | ± | 0.14 |  | 0.15 | ± | 0.06 |  | 0.11 | ± | 0.03 |  |
| PI (16:0-18:1) | 1.28 | ± | 0.32 |  | 1.03 | ± | 0.06 |  | 1.22 | ± | 0.15 |  | 1.16 | ± | 0.17 |  |
| PI (16:1-16:1) | 0.09 | ± | 0.05 |  | 0.01 | ± | 0.01 |  | 0.01 | ± | 0.01 |  | 0.03 | ± | 0.02 |  |
| PI (16:1-18:0) | 1.35 | ± | 0.12 |  | 1.17 | ± | 0.20 |  | 1.30 | ± | 0.25 |  | 1.29 | ± | 0.15 |  |
| PI (16:1-18:1) | 3.45 | ± | 0.36 |  | 3.15 | ± | 0.22 |  | 3.03 | ± | 0.44 |  | 3.08 | ± | 0.41 |  |
| PI (18:0-18:1) | 0.84 | ± | 0.13 | ^B^ | 1.94 | ± | 0.12 | ^A^ | 0.58 | ± | 0.13 | ^B^ | 2.38 | ± | 0.17 | ^A^ |
| PI (18:1-18:1) | 2.54 | ± | 0.15 | ^C^ | 2.00 | ± | 0.22 | ^C^ | 10.36 | ± | 0.87 | ^B^ | 12.63 | ± | 0.64 | ^A^ |
| PI (16:0-18:2) | 3.18 | ± | 0.62 |  | 3.73 | ± | 0.40 |  | 2.50 | ± | 0.57 |  | 2.28 | ± | 0.36 |  |
| PI (16:1-18:2) | 0.15 | ± | 0.03 |  | 0.13 | ± | 0.03 |  | 0.06 | ± | 0.02 |  | 0.15 | ± | 0.05 |  |
| PI (18:0-18:2) | 1.93 | ± | 0.29 | ^C^ | 2.40 | ± | 0.20 | ^C^ | 9.90 | ± | 0.41 | ^B^ | 14.05 | ± | 0.74 | ^A^ |
| PI (18:1-18:2) | 0.76 | ± | 0.27 |  | 1.20 | ± | 0.17 |  | 1.73 | ± | 0.26 |  | 1.28 | ± | 0.23 |  |
| PI (18:2-18:2) | 2.28 | ± | 0.40 |  | 2.39 | ± | 0.21 |  | 1.99 | ± | 0.27 |  | 1.67 | ± | 0.11 |  |
| PI (16:0-20:4) | 2.51 | ± | 0.42 |  | 2.81 | ± | 0.20 |  | 2.12 | ± | 0.23 |  | 1.86 | ± | 0.19 |  |
| PI (16:1-20:4) | 0.05 | ± | 0.02 | ^B^ | 0.17 | ± | 0.04 | ^AB^ | 0.44 | ± | 0.11 | ^A^ | 0.34 | ± | 0.12 | ^AB^ |
| PI (18:0-20:4) | 24.80 | ± | 1.83 | ^A^ | 18.81 | ± | 0.74 | ^B^ | 20.60 | ± | 0.87 | ^AB^ | 19.25 | ± | 1.23 | ^B^ |
| PI (18:1-20:4) | 5.38 | ± | 0.54 |  | 4.51 | ± | 0.93 |  | 5.69 | ± | 0.71 |  | 2.93 | ± | 0.57 |  |
| PI (18:2-20:4) | 5.18 | ± | 1.05 | ^AB^ | 6.45 | ± | 0.46 | ^A^ | 3.39 | ± | 0.49 | ^BC^ | 2.02 | ± | 0.55 | ^C^ |
| PI (16:0-20:5) | 0.07 | ± | 0.05 |  | 0.15 | ± | 0.09 |  | 0.28 | ± | 0.03 |  | 0.25 | ± | 0.05 |  |
| PI (16:1-20:5) | 0.00 | ± | 0.00 |  | 0.00 | ± | 0.00 |  | 0.00 | ± | 0.00 |  | 0.00 | ± | 0.00 |  |
| PI (18:0-20:5) | 1.85 | ± | 0.54 |  | 2.27 | ± | 0.24 |  | 1.98 | ± | 0.35 |  | 1.27 | ± | 0.22 |  |
| PI (18:1-20:5) | 6.06 | ± | 0.79 | ^A^ | 7.69 | ± | 0.54 | ^A^ | 3.01 | ± | 0.41 | ^B^ | 2.14 | ± | 0.17 | ^B^ |
| PI (18:2-20:5) | 0.03 | ± | 0.03 |  | 0.05 | ± | 0.04 |  | 0.07 | ± | 0.03 |  | 0.01 | ± | 0.01 |  |
| PI (16:0-22:6) | 5.14 | ± | 0.72 | ^A^ | 7.21 | ± | 0.54 | ^A^ | 2.99 | ± | 0.36 | ^B^ | 2.10 | ± | 0.40 | ^B^ |
| PI (16:1-22:6) | 0.01 | ± | 0.01 |  | 0.06 | ± | 0.04 |  | 0.04 | ± | 0.02 |  | 0.04 | ± | 0.04 |  |
| PI (18:0-22:6) | 25.82 | ± | 2.33 |  | 22.97 | ± | 1.19 |  | 21.33 | ± | 1.49 |  | 19.85 | ± | 1.08 |  |
| PI (18:1-22:6) | 1.81 | ± | 0.39 | ^B^ | 3.18 | ± | 0.49 | ^A^ | 1.54 | ± | 0.10 | ^B^ | 1.29 | ± | 0.23 | ^B^ |
| PI (18:2-22:6) | 0.38 | ± | 0.09 | ^AB^ | 0.75 | ± | 0.12 | ^A^ | 0.17 | ± | 0.10 | ^B^ | 0.16 | ± | 0.07 | ^B^ |
| 16:0-PI | 15.22 | ± | 1.56 | ^AB^ | 18.64 | ± | 1.03 | ^A^ | 12.76 | ± | 1.17 | ^B^ | 14.00 | ± | 0.81 | ^AB^ |
| 18:0-PI | 55.26 | ± | 3.55 |  | 48.44 | ± | 1.86 |  | 54.40 | ± | 2.17 |  | 56.94 | ± | 2.60 |  |

KO, knockout; PI, phosphatidylinositol.

Values are represented as means ± SEM (n = 6). Means without a common letter differ significantly (*P* < 0.05).

Tandem mass spectrometry (MS/MS) analyses were performed using multiple reaction monitoring (MRM) in negative ionization mode.**Supplemental Table S25. Phosphatidylglycerol profiles in the extensor digitorum longus (EDL) and soleus (SOL) from conditional lysophosphatidylglycerol acyltransferase 1-deficient (*LPGAT1* cKO) mice**

|  | % of total PG | | | | | | | | | | | | | | | |
| --- | --- | --- | --- | --- | --- | --- | --- | --- | --- | --- | --- | --- | --- | --- | --- | --- |
| Species | *LPGAT1*^flox/flox^-EDL | | | | *LPGAT1* cKO-EDL | | | | *LPGAT1*^flox/flox^-SOL | | | | *LPGAT1* cKO-SOL | | | |
| PG (16:0-16:0) | 1.92 | ± | 0.48 | ^A^ | 1.70 | ± | 0.41 | ^AB^ | 0.52 | ± | 0.15 | ^B^ | 0.51 | ± | 0.11 | ^B^ |
| PG (16:0-18:0) | 11.43 | ± | 1.90 |  | 10.77 | ± | 1.11 |  | 8.04 | ± | 1.05 |  | 11.78 | ± | 1.20 |  |
| PG (18:0-18:0) | 0.43 | ± | 0.20 |  | 0.36 | ± | 0.11 |  | 0.13 | ± | 0.04 |  | 0.44 | ± | 0.20 |  |
| PG (16:0-16:1) | 1.79 | ± | 0.32 | ^A^ | 1.59 | ± | 0.33 | ^A^ | 0.36 | ± | 0.20 | ^B^ | 0.31 | ± | 0.11 | ^B^ |
| PG (16:0-18:1) | 23.15 | ± | 1.93 | ^AB^ | 19.06 | ± | 1.28 | ^B^ | 26.68 | ± | 0.61 | ^A^ | 25.30 | ± | 1.33 | ^A^ |
| PG (16:1-16:1) | 0.19 | ± | 0.06 | ^AB^ | 0.32 | ± | 0.08 | ^A^ | 0.04 | ± | 0.02 | ^B^ | 0.01 | ± | 0.01 | ^B^ |
| PG (16:1-18:0) | 19.69 | ± | 2.63 | ^B^ | 20.04 | ± | 0.98 | ^B^ | 26.90 | ± | 0.92 | ^A^ | 30.67 | ± | 0.89 | ^A^ |
| PG (16:1-18:1) | 9.36 | ± | 1.18 | ^A^ | 9.58 | ± | 0.99 | ^A^ | 8.28 | ± | 1.27 | ^A^ | 2.86 | ± | 0.28 | ^B^ |
| PG (18:0-18:1) | 1.20 | ± | 0.28 |  | 1.90 | ± | 0.36 |  | 0.63 | ± | 0.22 |  | 1.86 | ± | 0.60 |  |
| PG (18:1-18:1) | 4.61 | ± | 0.40 |  | 4.09 | ± | 0.34 |  | 4.71 | ± | 0.44 |  | 3.76 | ± | 0.51 |  |
| PG (16:0-18:2) | 10.10 | ± | 1.22 | ^A^ | 11.84 | ± | 1.17 | ^A^ | 8.18 | ± | 1.55 | ^AB^ | 4.74 | ± | 0.55 | ^B^ |
| PG (16:1-18:2) | 0.76 | ± | 0.19 | ^AB^ | 1.37 | ± | 0.27 | ^A^ | 0.20 | ± | 0.08 | ^B^ | 0.30 | ± | 0.16 | ^B^ |
| PG (18:0-18:2) | 4.25 | ± | 1.13 |  | 4.75 | ± | 0.29 |  | 5.61 | ± | 0.48 |  | 5.41 | ± | 0.74 |  |
| PG (18:1-18:2) | 2.26 | ± | 0.41 |  | 2.95 | ± | 0.49 |  | 2.16 | ± | 0.53 |  | 2.79 | ± | 0.45 |  |
| PG (18:2-18:2) | 0.46 | ± | 0.20 |  | 0.68 | ± | 0.25 |  | 0.34 | ± | 0.14 |  | 0.44 | ± | 0.15 |  |
| PG (16:0-20:4) | 0.54 | ± | 0.21 |  | 0.67 | ± | 0.14 |  | 0.29 | ± | 0.10 |  | 0.79 | ± | 0.23 |  |
| PG (16:1-20:4) | 0.18 | ± | 0.07 |  | 0.20 | ± | 0.04 |  | 0.06 | ± | 0.04 |  | 0.20 | ± | 0.06 |  |
| PG (18:0-20:4) | 0.11 | ± | 0.05 |  | 0.07 | ± | 0.05 |  | 0.03 | ± | 0.02 |  | 0.14 | ± | 0.07 |  |
| PG (18:1-20:4) | 0.74 | ± | 0.13 |  | 0.89 | ± | 0.30 |  | 0.26 | ± | 0.09 |  | 0.44 | ± | 0.23 |  |
| PG (18:2-20:4) | 0.83 | ± | 0.21 | ^B^ | 2.21 | ± | 0.29 | ^A^ | 1.46 | ± | 0.30 | ^AB^ | 1.37 | ± | 0.29 | ^AB^ |
| PG (16:0-20:5) | 0.09 | ± | 0.04 |  | 0.11 | ± | 0.03 |  | 0.22 | ± | 0.09 |  | 0.36 | ± | 0.14 |  |
| PG (16:1-20:5) | 0.05 | ± | 0.05 |  | 0.04 | ± | 0.03 |  | 0.12 | ± | 0.07 |  | 0.06 | ± | 0.06 |  |
| PG (18:0-20:5) | 0.47 | ± | 0.13 |  | 0.72 | ± | 0.21 |  | 0.34 | ± | 0.07 |  | 0.30 | ± | 0.10 |  |
| PG (18:1-20:5) | 1.08 | ± | 0.30 |  | 1.13 | ± | 0.31 |  | 1.52 | ± | 0.37 |  | 1.55 | ± | 0.32 |  |
| PG (18:2-20:5) | 0.72 | ± | 0.24 |  | 0.50 | ± | 0.17 |  | 0.47 | ± | 0.20 |  | 0.12 | ± | 0.06 |  |
| PG (16:0-22:6) | 2.37 | ± | 0.43 |  | 1.36 | ± | 0.39 |  | 1.15 | ± | 0.20 |  | 1.35 | ± | 0.42 |  |
| PG (16:1-22:6) | 0.25 | ± | 0.17 |  | 0.46 | ± | 0.19 |  | 0.17 | ± | 0.03 |  | 0.69 | ± | 0.41 |  |
| PG (18:0-22:6) | 0.58 | ± | 0.20 |  | 0.23 | ± | 0.10 |  | 0.41 | ± | 0.11 |  | 0.21 | ± | 0.08 |  |
| PG (18:1-22:6) | 0.18 | ± | 0.08 |  | 0.04 | ± | 0.03 |  | 0.14 | ± | 0.09 |  | 0.16 | ± | 0.09 |  |
| PG (18:2-22:6) | 0.21 | ± | 0.10 | ^B^ | 0.38 | ± | 0.16 | ^AB^ | 0.56 | ± | 0.19 | ^AB^ | 1.08 | ± | 0.31 | ^A^ |
| 16:0-PG | 51.39 | ± | 3.21 |  | 47.10 | ± | 1.01 |  | 45.45 | ± | 0.74 |  | 45.15 | ± | 1.94 |  |
| 18:0-PG | 7.04 | ± | 1.20 |  | 8.03 | ± | 0.53 |  | 7.16 | ± | 0.64 |  | 8.36 | ± | 0.89 |  |
| 18:1-PG | 42.58 | ± | 1.60 | ^AB^ | 39.64 | ± | 1.78 | ^AB^ | 44.38 | ± | 0.66 | ^A^ | 38.71 | ± | 1.36 | ^B^ |
| 18:2-PG | 19.59 | ± | 0.98 | ^B^ | 24.67 | ± | 1.40 | ^A^ | 18.99 | ± | 1.49 | ^B^ | 16.23 | ± | 1.15 | ^B^ |

KP, knockout; PG, phosphatidylglycerol.

Values are represented as means ± SEM (n = 6). Means without a common letter differ significantly (*P* < 0.05).

Tandem mass spectrometry (MS/MS) analyses were performed using multiple reaction monitoring (MRM) in negative ionization mode.

**Supplemental Table S26. Conditions for precursor ion scanning and neutral loss scanning of individual classes of phospholipids in the positive** **ionization mode**

|  | Scanning mode | *m/z* range | CE |
| --- | --- | --- | --- |
| PC | Pre: *m/z* 184 (phosphoryl choline) | 600 - 1000 | -35 V |
| PE | NL: *m/z* 141 (phosphoryl ethanolamine) | 550 - 950 | -35 V |

CE, collision energy; NL, neutral loss scan; PC, phosphatidylcholine; PE, phosphatidylethanolamine; Pre, precursor ion scan.

**Supplemental Table S27. Multiple reaction monitoring (MRM) transitions used for liquid chromatography-tandem mass spectrometry (LC-MS/MS) analyses of PC and PE species** **containing palmitate-*d*_31_ and stearate-*d*_35_**

| Species | Q1 *m/z* | Q3 *m/z* |
| --- | --- | --- |
| PC (16:0-*d*_31_-22:6) | 881.6 | 286.3 |
| PC (18:0-*d*_35_-22:6) | 913.6 | 318.3 |
| PE (18:0-*d*_35_-22:6) | 825.5 | 318.3 |

At MS1, [M+HCOO]^-^ ions for PC and [M-H]^-^ ions for other PEs were selected. At MS3, the m/z values of 286.3 and 318.3 for those containing palmitate-*d*_31_ or stearate-*d*_35_.

**Supplemental Table S28. Mass spectrometry (MS) condition for multiple reaction monitoring (MRM) and product ion scanning**

|  | Scanning mode | Polarity | *m/z* range | CE |
| --- | --- | --- | --- | --- |
| PC (16:0-22:6) | MRM | Positive | 806.50 > 184.0 | -35 V |
|  | Product ion scan | Negative | 850.50 > 50.0 - 900.0 | -35 V |
| PC (18:0-22:6) | MRM | Positive | 834.50 > 184.0 | -35 V |
|  | Product ion scan | Negative | 878.50 > 50.0 - 900.0 | -35 V |

CE, collision energy; PC, phosphatidylcholine.

**Supplemental Table S29. Nucleotide sequences of primers for quantitative reverse transcription-polymerase chain reaction (qRT-PCR)**

|  | Forward | Reverse |
| --- | --- | --- |
| 36B4 | 5′-GGCCCTGCACTCTCGCTTTC-3′ | 5′-TGCCAGGACGCGCTTGT-3′ |
| 18S rRNA | 5′-GGGAGCCTGAGAAACGGC-3′ | 5′-GGGTCGGGAGTGGGTAATTTT-3′ |
| GPAT1 | 5′-AGCAAGTCCTGCGCTATCAT-3′ | 5′-CTCGTGTGGGTGATTGTGAC-3′ |
| GPAT2 | 5′- AAGAAAGAGGTACAGCGTATCC-3′ | 5′-GTGGAGAGCCCTCCTGCACAG-3′ |
| GPAT3 | 5′-GTACATGCCTCCCATGACTAG-3′ | 5′-GATCCGTTGCCCACGATCATC-3′ |
| GPAT4 | 5′-GTGGCAGGACAAGGTCAGAGCTACA-3′ | 5′-TCCCTCCTGACTCACCAGTTCTTCC-3′ |
| LPAAT1 | 5′-ACCAGAATGGAGCTGTGGCC-3′ | 5′-CGCTCCCCCAGGCTTCTTCA-3′ |
| LPAAT2 | 5′-AAGCGTGAGCTAATGTTCACAGG-3′ | 5′-TTTTTAAAGGGCAACAGGTCCC-3′ |
| LPAAT3 | 5′-TGTTCTCAGTGAAGGACCGT-3′ | 5′-CTTAAGCTCTTGGTTGCCAT-3′ |
| LPAAT4 | 5′-CAAGATCAATGCCAGACTCTGCT-3′ | 5′-AAACTTGTGATTGAGGACCACGA-3′ |
| AGPAT5 | 5′-AGAGGATGCTGCTGTCCCT-3′ | 5′-AACAAACCACAGGCAGCC-3′ |
| LPCAT1 | 5′-GTGCACGAGCTGCGACT-3′ | 5′-GCTGCTCTGGCTCCTTATCA-3′ |
| LPGAT1 | 5′-ACACAGTGATGGAATGGGGG-3′ | 5′-GAGCGACAACCGGTCCTTTA-3′ |
| LPGAT1 Exon 3 | 5′-ACACAGTGATGGAATGGGGG-3′ | 5′-CACATCTCCAGTTGCCTGGT-3′ |
| LCLAT1 | 5′-TGGATGTTCCTGTGGAACTGTCT-3′ | 5′-GGTTCATGGATGGCACAAAAATA-3′ |
| LPCAT2 | 5′-GTCCAGCAGACTACGATCAGTG-3′ | 5′-CTTATTGGATGGGTCAGCTTTTC-3′ |
| LPEAT2 | 5′-AGAGGGTTAAGTTCTGCCTCCT-3′ | 5′-CATACAGTCTTCCTCCATCCTGTAA-3′ |
| LPCAT3 | 5′-TCAGGATACCTGATTTGCTTCCA-3′ | 5′-GGATGGTCTGTTGCACCAAGTAG-3′ |
| LPCAT4 | 5′-TTCGGTTTCAGAGGATACGACAA-3′ | 5′-AATGTCTGGATTGTCGGACTGAA-3′ |
| LPEAT1 | 5′-CTGAAATGTGTGTGCTATGAGCG-3′ | 5′-TGGAAGAGAGGAAGTGGTGTCTG-3′ |
| LPIAT1 | 5′-ATACTGGAACATGACCGTGCAGT-3′ | 5′-TAGGTAGTAACCAGGGTGGAGGC-3′ |
| DDHD1 | 5′-ACCCAAAAACTGGGCTTTTC-3′ | 5′-CAGTGTGGCTTCTTCTACATAACC-3′ |
| DDHD2 | 5′-CCAGCCAATTGCAGGGTCTG-3′ | 5′-CGGTTCCCCACAGTGAATATC-3′ |
| p125 | 5′-GGATCCGATCTGAAGCAGGG-3′ | 5′-TCTTTTCTGCTTCAACTACTTGCT-3′ |

**Supplemental Table S30. Small interfering RNA (siRNA) sequences for the knockdown of target genes**

|  | siRNA sequences | |
| --- | --- | --- |
| Target genes | Guide (Anti sense) | Passenger (Sense) |
| GFP (control) | CUUGAAGAAGUCGUGCUGCdTdT | GCAGCACGACUUCUUCAAGdTdT |
| GPAT3 #1 | UACUGAAAGUUGACAUUGGdTdT | CCAAUGUCAACUUUCAGUAdTdT |
| GPAT3 #2 | UGAAUGAUCCCCAUCAAUCdCdA | GAUUGAUGGGGAUCAUUCAdTdT |
| GPAT3 #3 | AAAAAAAUGAACAAUAAACdAdA | GUUUAUUGUUCAUUUUUUUdTdT |
| LPCAT1 #1 | UUAUCUGAGGCCACUUUCCdAdT | GGAAAGUGGCCUCAGAUAAdTdT |
| LPCAT1 #2 | UCCUAUUUGUACAAGUUCCdTdT | GGAACUUGUACAAAUAGGAdTdT |
| LPCAT1 #3 | UCUUUUUCAAGAUUUUCUGdGdT | CAGAAAAUCUUGAAAAAGAdTdT |
| LPGAT1 #1 | AAUUACAUAGCAGAUGUAGdGdA | CUACAUCUGCUAUGUAAUUdTdT |
| LPGAT1 #2 | AAUUCCAAAGUUUGUAUACdTdT | GUAUACAAACUUUGGAAUUdTdT |
| LPGAT1 #3 | UCAAAAUAAUGUUUGUUGCdCdC | GCAACAAACAUUAUUUUGAdTdT |
| DDHD1 #1 | UAGAAAAGCCCAGUUUUUGdGdG | CAAAAACUGGGCUUUUCUAdTdT |
| DDHD1 #2 | UGUUUUUGAUGAUUCUUCCdTdT | GGAAGAAUCAUCAAAAACAdTdT |
| DDHD1 #3 | UCGUGUUUGUACAUGAAUGdTdT | CAUUCAUGUACAAACACGAdTdT |

**Supplemental Table S31. Multiple reaction monitoring (MRM) transitions used for liquid chromatography-tandem mass spectrometry (LC-MS/MS) analyses for phosphatidylcholine species**

| Species | Q1 *m/z* | Q3 *m/z* |
| --- | --- | --- |
| PC (16:0-16:0) | 778.55 | 255.3 |
| PC (16:0-18:0) | 806.6 | 283.3 |
| PC (18:0-18:0) | 834.65 | 283.3 |
| PC (16:0-16:1) | 776.55 | 253.2 |
| PC (16:0-18:1) | 804.6 | 281.3 |
| PC (16:1-16:1) | 774.55 | 253.2 |
| PC (16:1-18:0) | 804.6 | 253.2 |
| PC (16:1-18:1) | 802.55 | 281.3 |
| PC (18:0-18:1) | 832.6 | 281.3 |
| PC (18:1-18:1) | 830.6 | 281.3 |
| PC (16:0-18:2) | 802.6 | 279.3 |
| PC (16:1-18:2) | 800.6 | 279.3 |
| PC (18:0-18:2) | 830.6 | 279.3 |
| PC (18:1-18:2) | 828.6 | 279.3 |
| PC (18:2-18:2) | 826.6 | 279.3 |
| PC (16:0-20:4) | 826.6 | 303.3 |
| PC (16:1-20:4) | 824.6 | 303.3 |
| PC (18:0-20:4) | 854.6 | 303.3 |
| PC (18:1-20:4) | 852.6 | 303.3 |
| PC (18:2-20:4) | 850.6 | 303.3 |
| PC (16:0-20:5) | 800.6 | 277.2 |
| PC (16:1-20:5) | 798.5 | 277.2 |
| PC (18:0-20:5) | 828.6 | 277.2 |
| PC (18:1-20:5) | 826.6 | 277.2 |
| PC (18:2-20:5) | 824.6 | 277.2 |
| PC (16:0-22:6) | 852.6 | 329.2 |
| PC (16:1-22:6) | 850.6 | 329.2 |
| PC (18:0-22:6) | 880.6 | 329.2 |
| PC (18:1-22:6) | 878.6 | 329.2 |
| PC (18:2-22:6) | 876.6 | 329.2 |
| PC (17:0-17:0) | 806.6 | 269.2 |

PC, phosphatidylcholine.

**Supplemental Table S32. Multiple reaction monitoring (MRM) transitions used for liquid chromatography-tandem mass spectrometry (LC-MS/MS) analyses for phosphatidylethanolamine species**

| Species | Q1 *m/z* | Q3 *m/z* |
| --- | --- | --- |
| PE (16:0-16:0) | 690.5 | 255.3 |
| PE (16:0-18:0) | 718.55 | 283.3 |
| PE (18:0-18:0) | 746.6 | 283.3 |
| PE (16:0-16:1) | 688.5 | 253.2 |
| PE (16:0-18:1) | 716.55 | 281.3 |
| PE (16:1-16:1) | 686.5 | 253.2 |
| PE (16:1-18:0) | 716.55 | 253.2 |
| PE (16:1-18:1) | 714.5 | 281.3 |
| PE (18:0-18:1) | 744.55 | 281.3 |
| PE (18:1-18:1) | 742.55 | 281.3 |
| PE (16:0-18:2) | 714.5 | 279.3 |
| PE (16:1-18:2) | 712.5 | 279.3 |
| PE (18:0-18:2) | 742.5 | 279.3 |
| PE (18:1-18:2) | 740.5 | 279.3 |
| PE (18:2-18:2) | 738.5 | 279.3 |
| PE (16:0-20:4) | 738.5 | 303.3 |
| PE (16:1-20:4) | 736.5 | 303.3 |
| PE (18:0-20:4) | 766.5 | 303.3 |
| PE (18:1-20:4) | 764.5 | 303.3 |
| PE (18:2-20:4) | 762.5 | 303.3 |
| PE (16:0-20:5) | 712.5 | 277.2 |
| PE (16:1-20:5) | 710.5 | 277.2 |
| PE (18:0-20:5) | 740.5 | 277.2 |
| PE (18:1-20:5) | 738.5 | 277.2 |
| PE (18:2-20:5) | 736.5 | 277.2 |
| PE (16:0-22:6) | 764.5 | 329.2 |
| PE (16:1-22:6) | 762.5 | 329.2 |
| PE (18:0-22:6) | 792.6 | 329.2 |
| PE (18:1-22:6) | 790.5 | 329.2 |
| PE (18:2-22:6) | 788.5 | 329.2 |

PE, phosphatidylethanolamine.

**Supplemental Table S33. Multiple reaction monitoring (MRM)transitions used for liquid chromatography-tandem mass spectrometry (LC-MS/MS) analyses for phosphatidylserine species**

| Species | Q1 *m/z* | Q3 *m/z* |
| --- | --- | --- |
| PS (16:0-16:0) | 736.5 | 551.5 |
| PS (16:0-18:0) | 746.6 | 579.5 |
| PS (18:0-18:0) | 792.6 | 607.6 |
| PS (16:0-16:1) | 734.5 | 549.5 |
| PS (16:0-18:1) | 762.6 | 577.5 |
| PS (16:1-16:1) | 732.5 | 547.5 |
| PS (16:1-18:0) | 762.6 | 577.5 |
| PS (16:1-18:1) | 760.5 | 575.5 |
| PS (18:0-18:1) | 790.6 | 605.6 |
| PS (18:1-18:1) | 788.6 | 603.5 |
| PS (16:0-18:2) | 760.5 | 575.5 |
| PS (16:1-18:2) | 758.5 | 573.5 |
| PS (18:0-18:2) | 788.5 | 603.5 |
| PS (18:1-18:2) | 786.5 | 601.5 |
| PS (18:2-18:2) | 784.5 | 599.5 |
| PS (16:0-20:4) | 784.5 | 599.5 |
| PS (16:1-20:4) | 782.5 | 597.5 |
| PS (18:0-20:4) | 812.5 | 627.5 |
| PS (18:1-20:4) | 810.5 | 625.5 |
| PS (18:2-20:4) | 808.5 | 623.5 |
| PS (16:0-20:5) | 782.5 | 597.5 |
| PS (16:1-20:5) | 780.5 | 595.5 |
| PS (18:0-20:5) | 810.5 | 625.5 |
| PS (18:1-20:5) | 808.5 | 623.5 |
| PS (18:2-20:5) | 806.5 | 621.5 |
| PS (16:0-22:6) | 808.5 | 623.5 |
| PS (16:1-22:6) | 806.5 | 621.5 |
| PS (18:0-22:6) | 836.5 | 651.5 |
| PS (18:1-22:6) | 834.5 | 649.5 |
| PS (18:2-22:6) | 832.5 | 647.5 |

PS, phosphatidylserine. **Supplemental Table S34. Multiple reaction monitoring (MRM)transitions used for liquid chromatography-tandem mass spectrometry (LC-MS/MS) analyses for phosphatidylinositol species**

| Species | Q1 *m/z* | Q3 *m/z* |
| --- | --- | --- |
| PI (16:0-16:0) | 828.6 | 551.6 |
| PI (16:0-18:0) | 856.7 | 579.7 |
| PI (18:0-18:0) | 884.7 | 607.7 |
| PI (16:0-16:1) | 826.6 | 549.6 |
| PI (16:0-18:1) | 854.7 | 577.7 |
| PI (16:1-16:1) | 824.6 | 547.6 |
| PI (16:1-18:0) | 854.7 | 577.7 |
| PI (16:1-18:1) | 852.6 | 575.6 |
| PI (18:0-18:1) | 882.7 | 605.7 |
| PI (18:1-18:1) | 880.7 | 603.7 |
| PI (16:0-18:2) | 852.6 | 575.6 |
| PI (16:1-18:2) | 850.6 | 573.6 |
| PI (18:0-18:2) | 880.7 | 603.7 |
| PI (18:1-18:2) | 878.7 | 601.7 |
| PI (18:2-18:2) | 876.6 | 599.6 |
| PI (16:0-20:4) | 876.6 | 599.6 |
| PI (16:1-20:4) | 874.6 | 597.6 |
| PI (18:0-20:4) | 904.7 | 627.7 |
| PI (18:1-20:4) | 902.7 | 625.7 |
| PI (18:2-20:4) | 900.6 | 623.6 |
| PI (16:0-20:5) | 874.6 | 597.6 |
| PI (16:1-20:5) | 872.6 | 595.6 |
| PI (18:0-20:5) | 902.7 | 625.7 |
| PI (18:1-20:5) | 900.6 | 623.6 |
| PI (18:2-20:5) | 898.6 | 621.6 |
| PI (16:0-22:6) | 900.6 | 623.6 |
| PI (16:1-22:6) | 898.6 | 621.6 |
| PI (18:0-22:6) | 928.7 | 651.7 |
| PI (18:1-22:6) | 926.7 | 649.7 |
| PI (18:2-22:6) | 924.6 | 647.6 |

PI, phosphatidylinositol.

**Supplemental Table S35. Multiple reaction monitoring (MRM)transitions used for liquid chromatography-tandem mass spectrometry (LC-MS/MS) analyses for phosphatidylglycerol species**

| Species | Q1 *m/z* | Q3 *m/z* |
| --- | --- | --- |
| PG (16:0-16:0) | 740.5 | 551.5 |
| PG (16:0-18:0) | 768.6 | 579.7 |
| PG (18:0-18:0) | 796.6 | 607.6 |
| PG (16:0-16:1) | 738.5 | 549.5 |
| PG (16:0-18:1) | 766.6 | 577.6 |
| PG (16:1-16:1) | 736.5 | 547.5 |
| PG (16:1-18:0) | 766.6 | 577.6 |
| PG (16:1-18:1) | 764.6 | 575.5 |
| PG (18:0-18:1) | 794.6 | 605.6 |
| PG (18:1-18:1) | 792.6 | 603.6 |
| PG (16:0-18:2) | 764.5 | 575.4 |
| PG (16:1-18:2) | 762.5 | 573.5 |
| PG (18:0-18:2) | 792.6 | 603.5 |
| PG (18:1-18:2) | 790.5 | 601.4 |
| PG (18:2-18:2) | 788.5 | 599.4 |
| PG (16:0-20:4) | 788.5 | 599.4 |
| PG (16:1-20:4) | 786.5 | 597.4 |
| PG (18:0-20:4) | 816.6 | 627.5 |
| PG (18:1-20:4) | 814.5 | 625.5 |
| PG (18:2-20:4) | 812.5 | 623.4 |
| PG (16:0-20:5) | 786.5 | 597.4 |
| PG (16:1-20:5) | 784.5 | 595.4 |
| PG (18:0-20:5) | 814.5 | 625.4 |
| PG (18:1-20:5) | 812.5 | 623.4 |
| PG (18:2-20:5) | 810.5 | 621.4 |
| PG (16:0-22:6) | 812.5 | 623.4 |
| PG (16:1-22:6) | 810.5 | 621.4 |
| PG (18:0-22:6) | 840.6 | 651.5 |
| PG (18:1-22:6) | 838.5 | 649.4 |
| PG (18:2-22:6) | 832.5 | 647.5 |

PG, phosphatidylglycerol.

**Supplemental Table S36. Multiple reaction monitoring (MRM) transitions used for liquid chromatography-tandem mass spectrometry (LC-MS/MS) analyses for tetralinoleoyl-cardiolipin**

| Species | Q1 *m/z* | Q3 *m/z* |
| --- | --- | --- |
| CL (18:2)_4_ | 1448 | 279.2 |
| CL (14:0)_4_ | 1240 | 227.2 |

CL (18:2)_4_, tetralinoleoyl-cardiolipin

CL (14:0)_4_, tetramyristoyl-cardiolipin
