# Supplementary material for: LPGAT1/LPLAT7 regulates acyl chain profiles at the sn-1 position of phospholipids in murine skeletal muscles
Source: J Biol Chem. 2023 May 20;299(7):104848. doi: 10.1016/j.jbc.2023.104848 (PMC10285227; doi:10.1016/j.jbc.2023.104848)
Supplement: Supplemental Figures [file mmc2.pdf]

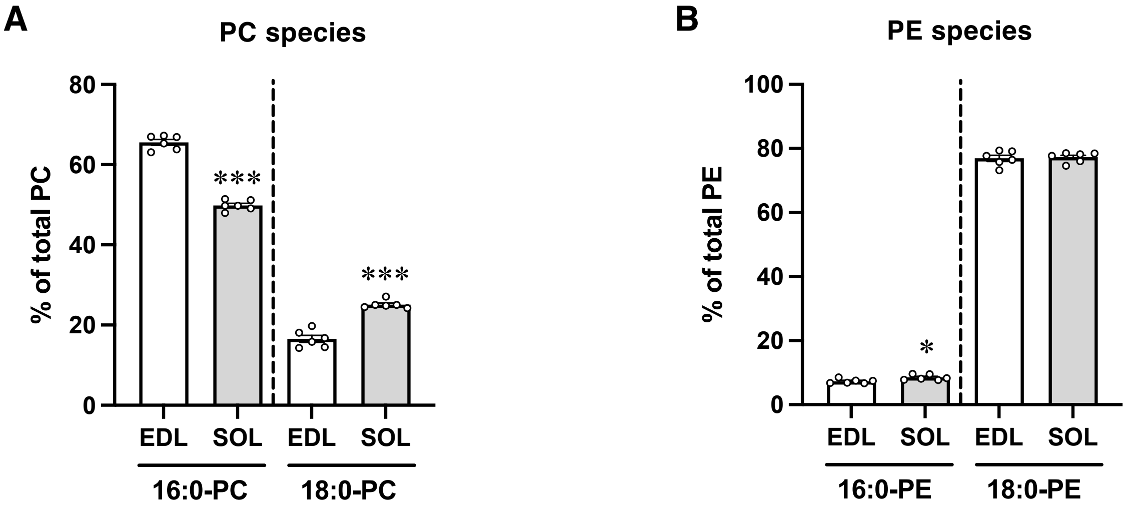

Supplemental Figure S1. Differences in the acyl chain profiles of phospholipids in rat skeletal muscles

(A), (B) Amounts of 16:0 and 18:0-PC (A) and PE (B) in the extensor digitorum longus (EDL) and soleus (SOL) muscles of Sprague-Dawley rats. Values are represented as the mean ± SEM (n = 6). ****P* < 0.001; **P* < 0.05 (vs. EDL).

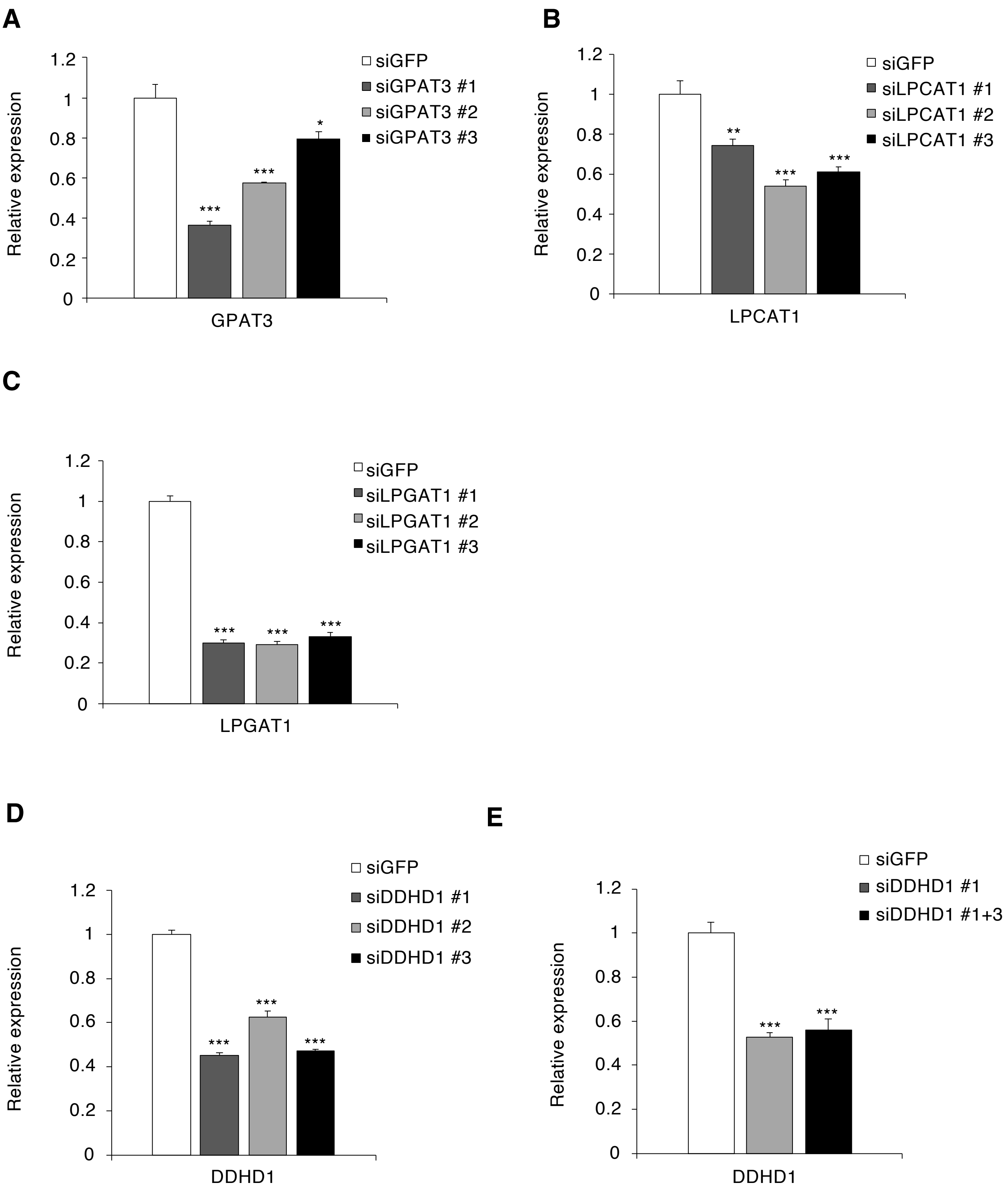

Supplemental Figure S2. Knockdown efficiencies of small interfering glycerol-3-phosphate acyltransferase 3 (siGPAT3), small interfering lysophosphatidylcholine acyltransferase 1 (siLPCAT1), small interfering lysophosphatidylglycerol acyltransferase 1 (siLPGAT1), and small interfering DDHD domain-containing protein 1 (siDDHD1) in C2C12 myotubes

Result of quantitative reverse transcription-polymerase chain reaction (qRT-PCR) analysis of GPAT3 (A), LPCAT1 (B), LPGAT1 (C), and DDHD1 (D, E) to confirm the knockdown efficiencies of small interfering RNAs (siRNAs) in C2C12 myotubes. Values are represented as the mean ± SEM (n = 3). ***$P < 0.001$; **$P < 0.01$; *$P < 0.05$ (vs. siGFP).

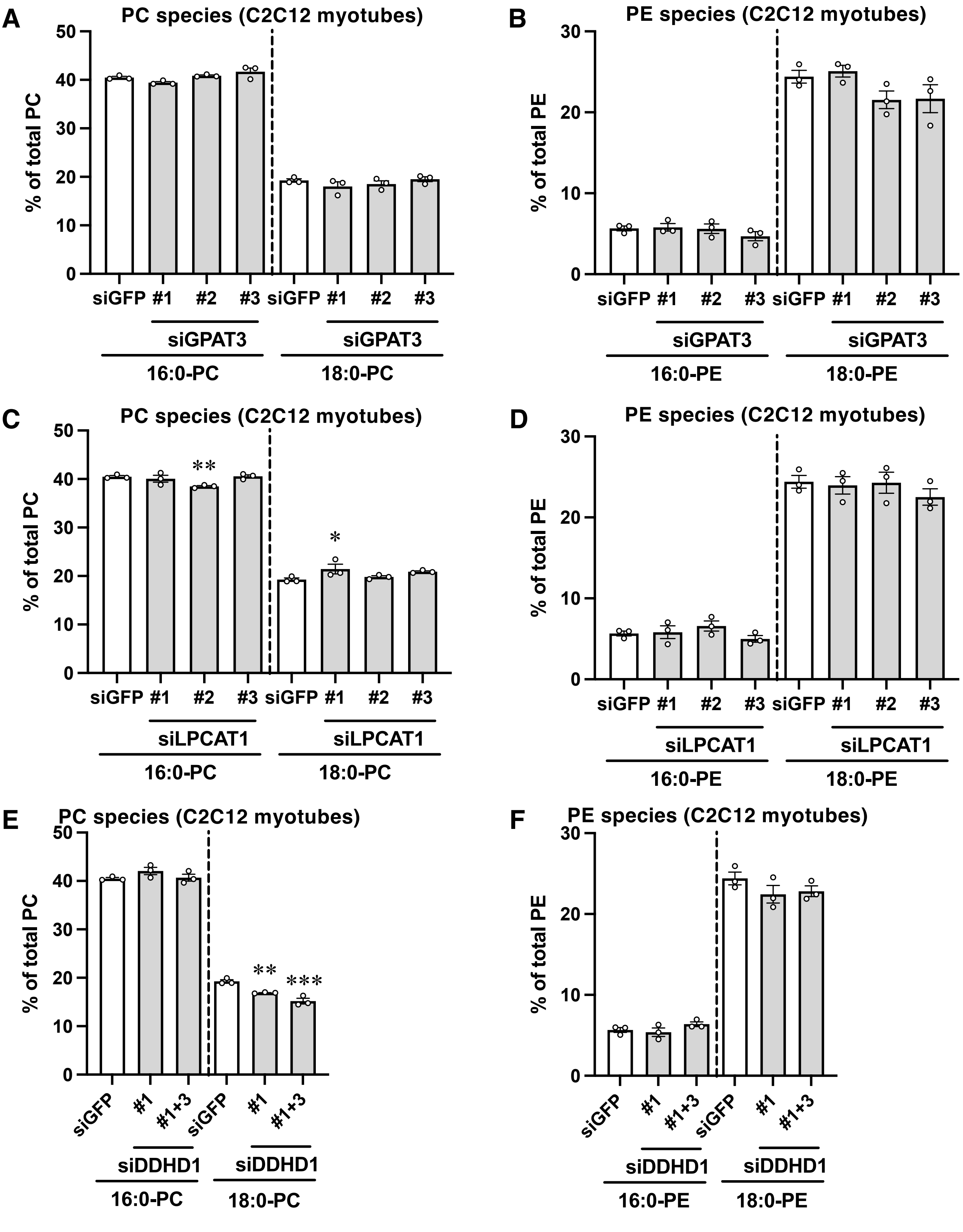

Supplemental Figure S3. Changes in the acyl chain profiles of PC and PE in C2C12 myotubes by knockdown of glycerol-3-phosphate acyltransferase 3 (GPAT3), lysophosphatidylcholine acyltransferase 1 (LPCAT1), and DDHD domain-containing 1 (DDHD1)

Amounts of 16:0 and 18:0-PC (A, C, E) and PE (B, D, F) in siGPAT3 (A, B), siLPCAT1 (C, D), and siDDHD1 (E, F)-transfected C2C12 myotubes. siGFP was used as the control. Tandem mass spectrometry (MS/MS) analyses were performed using multiple reaction monitoring (MRM) in negative ionization mode. Values are represented as the mean ± SEM (n = 3). *$P < 0.05$; **$P < 0.01$; ***$P < 0.001$ (vs. siGFP).

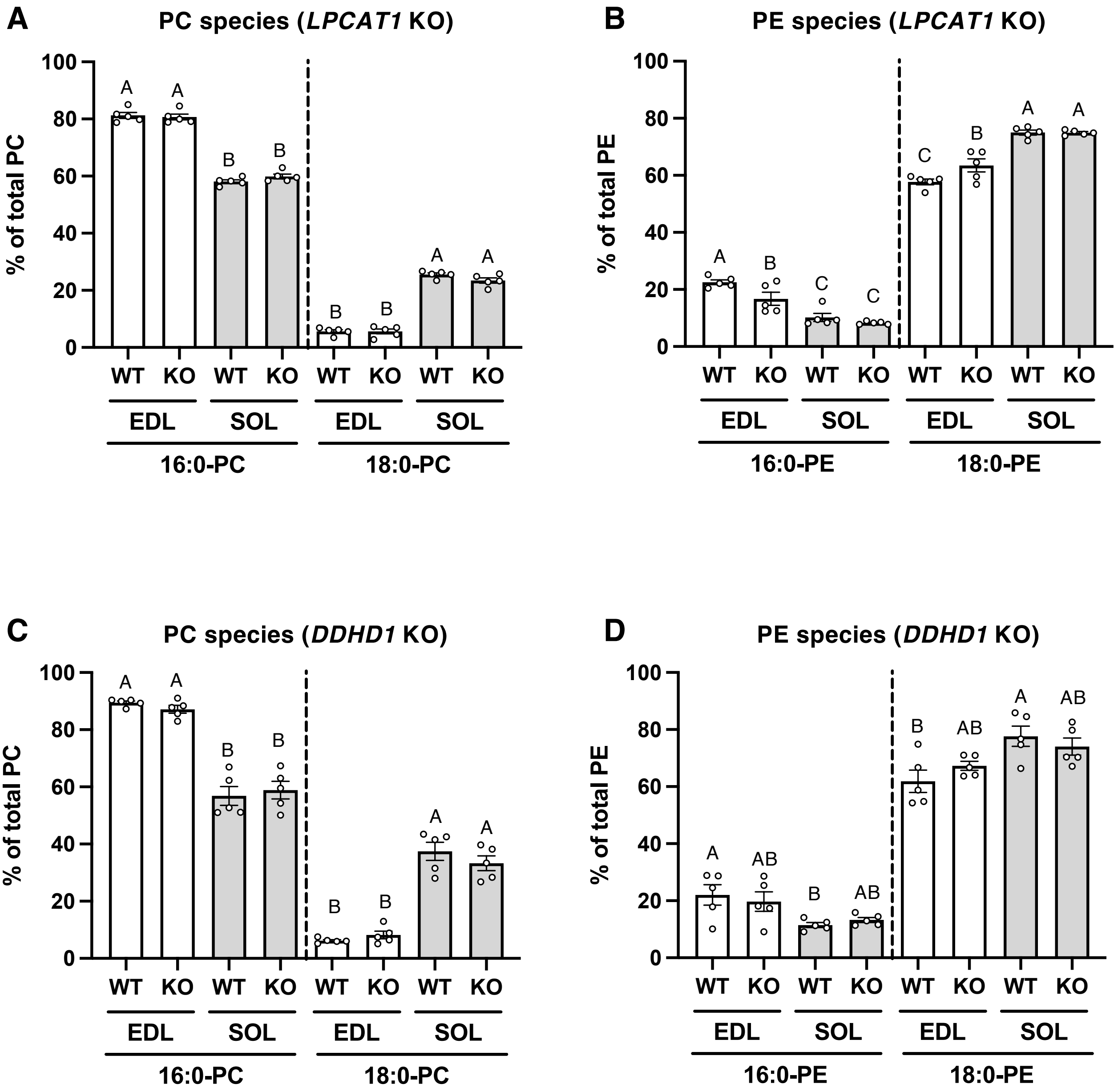

Supplemental Figure S4. Changes in the acyl chain profiles of PC and PE in murine skeletal muscles following global knockout of LPCAT1 and DDHD1
Amounts of 16:0 and 18:0-PC (A, C) and PE (B, D) in the LPCAT1- (A, B) and DDHD1-(C, D) deficient (KO) EDL and SOL muscles. Precursor ion scan or neutral loss scan mode tandem mass spectrometry (MS/MS) was performed in the positive ionization mode to measure PC and PE, respectively. Values are represented as mean ± SEM (n = 3–5). Means without a common letter differ significantly ($p <$ 0.05).

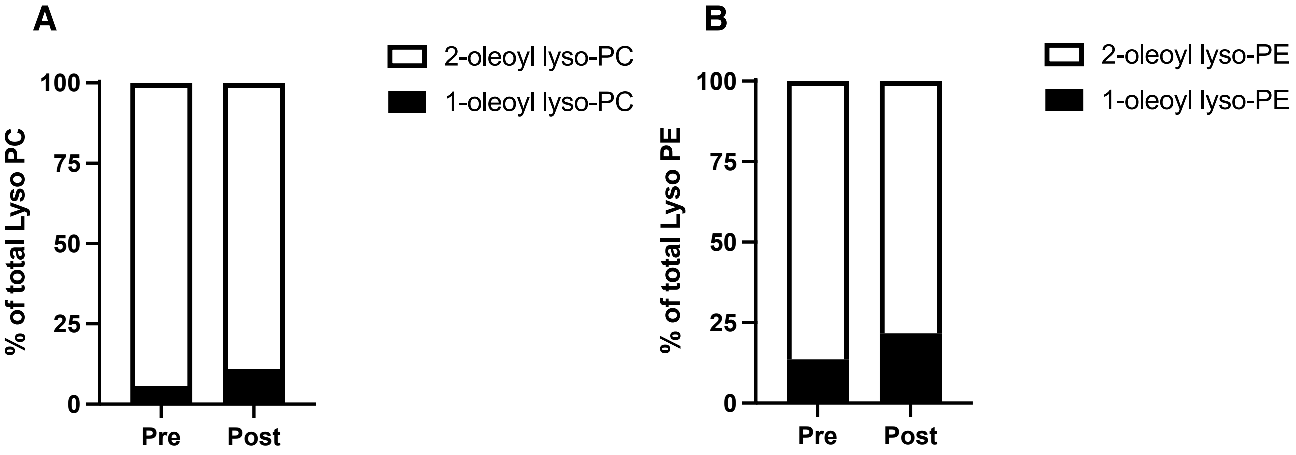

Supplemental Figure S5. *sn*-2-rich lyso-PCs and lyso-PEs used in this study
The ratio of *sn*-1 and *sn*-2 lysophospholipids isomers in *sn*-2-rich preparations, which were prepared from (A) dioleoyl PC and (B) dioleoyl PE. The ratio of these isomers in the reaction buffer was analyzed both before (Pre) and after (Post) the measurement of lyso-PC acyltransferase (LPCAT) or lyso-PE acyltransferase (LPEAT) activities.

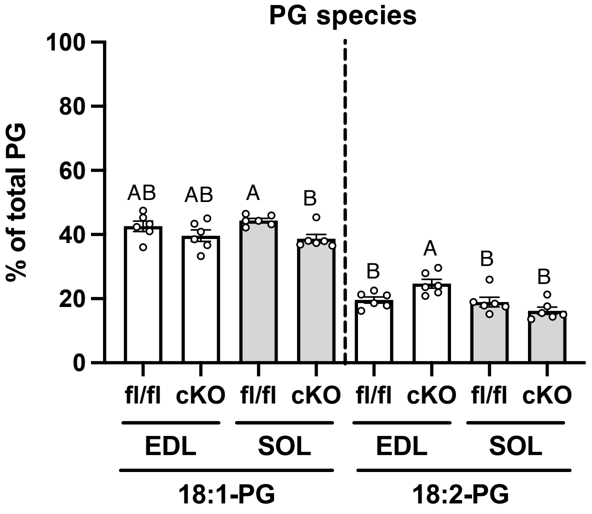

Supplemental Figure S6. Changes of acyl chain profiles in PGs of mice lacking *LPGAT1* in the skeletal muscle

Amounts of 18:1-PG and 18:2-PG in the EDL and SOL muscles of LPGAT1$^{flox/flox}$ mice (fl/fl) and *LPGAT1* cKO mice (cKO). Tandem mass spectrometry (MS/MS) analyses were performed using multiple reaction monitoring (MRM) in negative ionization mode. Values are represented as the mean ± SEM (n = 6). Means without a common letter differ significantly (P < 0.05).
